# Supplementary material for: Is there an association between disease ignorance and self-rated health? The HUNT Study, a cross-sectional survey
Source: BMJ Open. 2014 May 28;4(5):e004962. doi: 10.1136/bmjopen-2014-004962 (PMC4039843; doi:10.1136/bmjopen-2014-004962)
Supplement: Author's manuscript [file bmjopen-2014-004962.draft_revisions.pdf]

Is there an association between disease ignorance and self-rated health? The HUNT Study, a cross-sectional survey

Journal:	*BMJ Open*
Manuscript ID:	bmjopen-2014-004962
Article Type:	Research
Date Submitted by the Author:	29-Jan-2014
Complete List of Authors:	Jørgensen, Pål; Norwegian University of Science and Technology, Department of Public Health and General Practice<br>Langhammer, Arnulf; Norwegian University of Science and Technology, HUNT Research Centre<br>Krokstad, Steinar; Norwegian University of Science and Technology, HUNT Research Centre<br>Forsmo, Siri; Norwegian University of Science and Technology, Department of Public Health and General Practice
**Primary Subject Heading**:	General practice / Family practice
Secondary Subject Heading:	Epidemiology, Public health, Diabetes and endocrinology
Keywords:	Hypertension < CARDIOLOGY, General diabetes < DIABETES & ENDOCRINOLOGY, Thyroid disease < DIABETES & ENDOCRINOLOGY, PRIMARY CARE

SCHOLARONE™
Manuscripts

Disease ignorance and self-rated health, any relationship? The HUNT Study, a cross-sectional survey

Pål Jørgensen, Arnulf Langhammer, Steinar Krokstad, Siri Forsmo

Department of Public Health and General Practice, Norwegian University of Science and Technology, 7489 Trondheim, Norway Pål Jørgensen
PhD Candidate
HUNT Research Centre, Department of Public Health and General Practice, Norwegian University of Science and Technology, 7600 Levanger, Norway Arnulf Langhammer
Professor, Head of HUNT Databank
HUNT Research Centre, Department of Public Health and General Practice, Norwegian University of Science and Technology, Steinar Krokstad
Professor, Head of HUNT Research Center
Department of Public Health and General Practice, Norwegian University of Science and Technology, Siri Forsmo
Professor, Head of Department

Correspondence to: P Jørgensen pal_jorgensen@ntnu.no

Keywords: Self-rated health, awareness, hypothyroidism, diabetes, hypertension.

Word count: 2995

For peer review only

ABSTRACT

Objective To explore if awareness versus unawareness of thyroid dysfunction, diabetes mellitus or hypertension is associated with self-rated health.

Design Large-scale, cross sectional population based study. The association between thyroid function, diabetes mellitus, and blood pressure and self-rated health was explored by multiple logistic regression analysis.

Setting The second survey of the Nord-Trøndelag Health Study, HUNT2, 1995-97.

Participants 33,734 persons aged 40-70 years.

Primary outcome measures Logistic regression was used to estimate odds ratios for good self-rated health as a function of thyroid status, diabetes mellitus status and blood pressure status.

Results Persons aware of their hypothyroidism, diabetes mellitus, or hypertension reported poorer self-rated health than individuals without such conditions. Women with unknown and subclinical hypothyroidism reported better self-rated health than women with normal thyroid status. Both in women and men, unknown and probable diabetes, as well as unknown mild/moderate hypertension was not associated with poorer health. Further, persons with unknown severe hypertension reported better health than normotensive persons.

Conclusions People with undiagnosed, but prevalent hypothyroidism, diabetes mellitus, and hypertension often have good self-rated health, whilst when aware of their diagnoses, they report reduced self-rated health. Use of screening, more sensitive tests, and widened diagnostic criteria might have a negative effect on perceived health in the population.

For peer review only

STRENGTHS AND LIMITATIONS OF THIS STUDY

Strengths

- Sample from a large-scale, general population
- High-prevalent diseases under study; ensuring statistical power in subgroup analyses

Limitations

- Study mainly based on self-reported data
- Cross-sectional study; susceptibility to confounding and impossibility to assume causal relationships

For peer review only

INTRODUCTION

Guidelines for prevention and treatment have been developed for most high prevalent diseases in western countries aiming for reduction of morbidity and mortality by interventions mainly in primary health care (PHC).

In the society there seems to be an increasing conviction of achievable zero-vision regarding risks and diseases. Part of the strategy is to detect risk factors and pre-diseases in even earlier stages. From a secondary or tertiary health care level, this might seem reasonable since intervention on many individuals with specific risk factors presumably can prevent or delay disease or progression of disease. Further, health authorities and hospital clinicians regularly raise concern of the lack of detection of risk factors, of subclinical conditions and of achieving treatment goals.[1-7] Norwegian studies have shown that guidelines are often difficult to implement and adhere to in PHC.[8 9] According to guidelines, most individuals would be defined as at risk and resources needed to handle this appropriately could destabilize the entire health care system.[10-12] An American review pointed out knowledge, attitude,

and behaviour as barriers of physician's adherence to clinical guidelines.[13] In an already complex and busy PHC-setting, one might expect that resources used for disease prevention and case finding have to compete with resources for handling acknowledged disease. Also, physicians might want to avoid increasing disease related burden for patients, in line with the old wisdom; "primum non nocere".[14] Thus the risk and disease zero-visions in society and among politicians are seldom shared by PHC professionals.

When guidelines, mainly based on research from high risk hospital populations, are applied on low risk populations in PHC, more healthy individuals are identified as being at risk or are given diagnoses. Also the widened inclusion criteria for diagnoses in general and use of more sensitive tests contribute to define more individuals at risk or as unhealthy.[15] Possible undesirable outcomes of such strategies remain unclear.

Self-rated health (SRH) is a valid and widely used measure of general health in epidemiologic research.[16-19] It is associated with several clinical conditions often seen in PHC, with recovery [20-23] and is found to predict morbidity, sick leave, and disability pension,[24 25] as well as mortality.[26 27] The majority of studies describing association between labelling of disease per se and SRH have focused on arterial hypertension.[28-31] However, one study has indicated reduced SRH among individuals labelled with thyroid dysfunctions.[32]

The aim of this study was to investigate whether persons' awareness versus unawareness of thyroid dysfunction, diabetes mellitus, or hypertension was associated with their SRH, as reported in a population-based health study in Norway.

For peer review only

METHODS

Study population

The data sample in this study stem from the second wave of the Nord-Trøndelag Health Study (HUNT2) conducted in 1995-97 in the county of Nord-Trøndelag, Norway. All individuals aged 20 years and older, living in the county, were invited (94,194 individuals). In all, 66.7% of men (n=30,860) and 75.5% of women (n=35,280) participated. The survey consisted of both questionnaires and measurements, and is described previously in detail.[33 34] In our study we included answers from the main questionnaire and the baseline measurements for persons aged 40-70 years. The age span was chosen because thyroid stimulating hormone (TSH) was analysed in all women and in 50% of men at this age, of a rather low disease burden in people younger than 40 years, and of a lower attendance rate under and above this age span. A total of 24,950 individuals had TSH measurements and answered thyroid questions, thus were eligible for analysis on thyroid dysfunction, whilst in the analysis of diabetes mellitus and blood pressure, 33,734 individuals were included in all.

Self-rated health

The first question in the main questionnaire in HUNT2, answered before attending the examination stations, was "How is your health at the moment?". The four answer alternatives were dichotomized into poor or good (reference); good covering the answers "good" and "very good", poor covering "poor" and "not so good".

Thyroid function

The participants answered questions on history of hypo- and hyperthyroidism, goitre, other thyroid diseases, and treatment with thyroxin, radio-iodine, surgery, or thyreostatic medication.

For peer review only

Serum TSH and free T4 were analysed at the Hormone laboratory, Aker University Hospital, Norway. The laboratory reference value for TSH, as defined prior to the survey, was 0.2-4.5 mU/L and for free T_4 8.0-20.0 pmol/L. If TSH was <0.2 mU/L or >4.0 mU/L, and/or if the participant reported any thyroid disease, serum free T_4 was also measured.[35]

Individuals reporting no previous thyroid disease and having TSH within reference range were categorized as “no thyroid disease” and chosen as reference category. No previous thyroid disease combined with TSH >4.5 mU/L and free T_4 <8.0 pmol/L was defined as unknown hypothyroidism. No previous thyroid disease combined with TSH >4.5 mU/L and free T_4 8.0 – 20.0 pmol/L was defined as subclinical hypothyroidism. Individuals reporting hypothyroidism and use of thyroxin were classified as having known hypothyroidism, regardless of the TSH and T4 levels. Affirmative answers to other thyroid related questions or measures outside reference range in the remainders were classified as other thyroid dysfunction.

Diabetes mellitus

Diabetes mellitus was assessed through self-report and blood samples. Serum glucose was analysed at Levanger Hospital, Norway. Those reporting no diabetes and having normal glucose levels (<5.5 mmol/L) were classified as “no diabetes” and were chosen as reference category. No self-reported diabetes and non-fasting glucose >11.0 mmol/L was categorized as unknown diabetes, whereas no diabetes and non-fasting glucose 5.5-11.0 mmol/L was categorized as probable diabetes. Self-reported diabetes was classified as known diabetes regardless of the glucose level.

For peer review only

Blood pressure

In the questionnaire participants were asked about the doctor's advice after the latest blood pressure measurement prior to participation in HUNT. The answer categories were: "no follow-up and no medication necessary", "recommended follow-up examination but not to take medicine", "start or continue taking medicine for high blood pressure", or "never measured". At HUNT2, mean systolic and mean diastolic arterial blood pressure (BP) of measurement 2 and 3 was categorized into normal (systolic (s) BP < 140 mmHg and diastolic (d) BP < 90 mmHg), mild hypertension (sBP 140-159 mmHg and dBP <100 mmHg or sBP <160 mmHg and dBP 90-99 mmHg), moderate hypertension (sBP 160-179 mmHg and dBP <109 mmHg or sBP <180 mmHg and dBP 100-109 mmHg), and severe hypertension (sBP>180 mmHg or dBP>110 mmHg). We constructed a new variable to define normotensive (reference), unknown mild- and moderate hypertensive, unknown severe hypertensive, and known hypertensive persons on the basis of self-report and measures.

Statistical analysis

The descriptive analyses of the study population were stratified by gender, and we used chi-square tests to examine any difference in proportions of SRH between the independent variables. Gender-stratified multiple logistic regression were used to estimate odds ratio (OR) with 95% confidence intervals (CI) for good SRH, as a function of thyroid status, diabetes mellitus status and blood pressure status, in separate univariate and multivariate analyses for each condition.

Age, smoking, alcohol consumption, body mass index (BMI), working- and educational status, and self-reported limiting long-term illness or injury are associated with SRH[36] and the diseases under study, but likely not affected by SRH or the diseases. Hence, these

For peer review only

variables were included, a priori, as confounders in the models. Age was categorized into age groups; 40-49 years, 50-59 years, and 60-70 years. Smoking status was categorized into never smoked daily, previous daily smoker, and current daily smoker. Alcohol units (AU) were defined as number of glasses of wine, beer or liquor. Those reporting to be teetotalers or to have alcohol intake less than four times a month or less than seven AU per two weeks were categorized as low consumers, those reporting drinking five to eight times a month or 8-14 AU per two weeks as moderate consumers, and those drinking more often than eight times a month or more than 14 AU as heavy consumers. BMI ($kg/m^{2)}$ was calculated of measured height and weight and categorized according to The World Health Organization's (WHO) definition; underweight (18.5 kg/m^2), normal weight (18.5-24.9 kg/m^2), overweight (25.0-29.9 kg/m^2) and obese (>30.0 kg/m^2). People reporting paid- or self-employed work were classified as working, otherwise as not working. Educational level was categorized into <10 years, 10-12 years and >12 years. We chose affirmative answer to the question "Do you suffer from any long-term illness or injury (at least one year) of a physical or psychological nature that impairs your functioning in your everyday life?" to represent all relevant chronic medical conditions that could confound the results.

To examine whether the association of the three disease statuses with SRH differed by categories of the other independent variables, we used likelihood ratio-tests with p-value for statistical interaction. We tested for multicollinearity between the independent variables, by linear regression.

In an additional analysis, the association between SRH and having had one or more medical consultations during the last year was investigated by logistic regression models, stratified by gender, both in the total study population and after exclusion of persons with diagnoses under study.

For peer review only

All analyses were preformed with IBM SPSS Statistics version 20 for windows.

The study was approved by the Regional Committee for Medical Research. All participants signed written informed consent.

RESULTS

In all age categories, a higher proportion of men than women reported good SRH ($p<0.001$), and in both sexes, the proportion reporting good SRH declined by age ($p<0.002$) (Table 1). The proportion reporting good SRH was lower in overweight, obese, and underweight women, than in normal weight women ($p<0.001$). In men the proportion reporting good SRH declined between the normal weight group to overweight-, obese-, and underweight group ($p<0.001$). In previous and current female smokers, a lower proportion reported good SRH compared to non smokers ($p<0.001$). In men, the proportion reporting good SRH was higher among non smokers than among previous and current smokers ($p<0.001$), whereas there was no difference between previous and current smokers. The proportion reporting good SRH was higher in moderate and heavy alcohol consumers than in low consumers, increased with education and was higher for participants in paid work ($p<0.001$). Underweight men and persons with “any long-term impairment” had the lowest proportion reporting good SRH of all groups.

There was no multicollinearity between the independent variables.

Thyroid function

Thyroid dysfunctions, both known and unknown were more often observed among women than among men ($p<0.001$) (Table 1). Women with known hypothyroidism had lower odds of reporting good SRH (OR 0.49 (95% CI 0.41 to 0.59) compared to women without thyroid disease in the adjusted analyses, but women with unknown or subclinical hypothyroidism had

For peer review only - http://bmjopen.bmj.com/site/about/guidelines.xhtml

84% and 48% higher odds, respectively, of reporting good SRH compared to the odds of women without thyroid disease (Table 2). A corresponding, but non-significant pattern was found among men.

Diabetes mellitus

The prevalence of unknown, probable, and known diabetes was slightly higher in men than in women ($p<0.001$) (Table 1). Women with known diabetes mellitus had lower odds of good SRH than those without diabetes in the adjusted analyses; OR 0.53 (95% CI 0.41 to 0.69), whereas in women with unknown or possible diabetes mellitus, the odds of good SRH were similar to the odds among persons without diabetes, in the adjusted analyses (Table 2). In men, the association of diabetes status with SRH differed by levels of education. Among men without higher education (12 years or less), the odds of good SRH were as in the main-effect model (Table 2). However in men with higher education, the ORs of good SRH were barely significantly lower among men with unknown diabetes (OR 0.29 (95% CI 0.09-1.00)) and among men with possible diabetes (OR 0.79 (95% CI 0.63-1.00)) compared to men without diabetes. Among men with known diabetes in this stratum, the OR of good SRH was 0.31 (95% CI 0.17-0.54) compared to men without diabetes.

Blood pressure

The prevalence of unknown mild and moderate hypertension was higher in men than in women ($p<0.001$). Unknown severe hypertension and known hypertension were equally distributed between women and men (Table 1). Women with known hypertension had lower odds of reporting good SRH than normotensive women, in the adjusted analyses. The figures were similar in men (Table 2). In contrast; compared to normotensive women, those with unknown severe hypertension had 52 % higher odds of reporting good SRH, with similar

figures in men. Persons with unknown mild and moderate hypertension reported good SRH similar to the normotensive ones.

Additional analyses

Women with poor SRH had more than six times the odds of those with good SRH to have had a medical consultation during the last year; OR 6.29 (95% CI 5.47 to 7.22). For men the corresponding OR was 5.53 (95% CI 4.86 to 6.29). After exclusion of persons with diagnosed thyroid disease, diabetes mellitus, known hypertension, and "any long-term disease", the corresponding OR was 3.71 (95% CI 2.90 to 4.73), with similar figures in men.

DISCUSSION

This large population-based study showed that persons with known thyroid dysfunction, diabetes mellitus, and hypertension were less likely to report good SRH than those without such conditions. Less expected, persons with unknown and subclinical hypothyroidism were more likely to report good SRH than those without thyroid disease. Similarly, those with unknown severe hypertension were more likely to report good SRH, compared to persons with normal blood pressure. In general, persons with unknown diabetes and unknown mild/moderate hypertension, reported good health, just like the reference group.

Although both a qualitative and quantitative measure of association, the estimated ORs in our study will, if interpreted as relative risks, overestimate the case, because the prevalence of good SRH is high in all groups.[37]

The main strengths of this study were the numbers of participants, that the diseases we studied were high-prevalent, and that we could assume representativeness to the general population regarding the variables included. It is known that individuals with high burden of symptoms are less likely to attend surveys, but so far there has been little evidence that non-participation

For peer review only

on this basis introduce substantial bias in associational studies.[38] We studied diseases with generally low symptom-burden, and expect selection bias to be negligible.

The main limitation was possibly that we relied on self-reported data on both dependent and independent variables. The validity of self reported measures relevant for this study is questionable. However, if any, there should be a non-differential misclassification, that only should underestimate the associations found.

There is always a possibility of residual confounding in non-randomized study designs, and due to the observational, cross-sectional design we neither can assume a causal relationship.

Bias due to differential detection of disease in the study population could lead to a type 1 error. Hypothyroidism and diabetes mellitus are often associated with vague symptoms such as tiredness and weakness. These symptoms are strongly associated with reduced SRH.[39] It is likely that presenting such symptoms for the GP would result in measurements of TSH, free T4, and serum glucose, thus reveal any related dysfunction. Low self-perceived health increases the probability to visit a GP.[40] The social security covers most costs related to clinical measurements and blood sampling in Norway, thus we expect GPs to have a low threshold to measure thyroid function, blood pressure or glucose levels. People who do not consult their GP will not have diseases with mild or no symptoms diagnosed, and their personality could be characterized by less worrying and more optimistic attitude to health being reflected in better SRH.[41]

On the other hand, the association between known disease and poor SRH could in fact be explained physiologically due to the pathological effect of disease. Lack of corresponding association between ignored, but prevalent disease and poor SRH is not in line with this

For peer review only

hypothesis, rising question of a possible adverse effect of disease labelling. However, confounding by severity of disease cannot be ruled out.

Due to low numbers of persons having unknown hypothyroidism and diabetes mellitus, the results should be interpreted with caution. On the other hand, there were high numbers in the subclinical and probable groups, and analyses of these groups did not show any associations with poor SRH, also rising question of a possible disease labelling effect.

Consistent findings regarding unknown, subclinical, and probable disease, versus known disease, could indicate a potential adverse effect of disease labelling. Although early detection of disease is protective on morbidity and mortality for many diseases, low SRH is also shown to be associated morbidity and mortality.[25-27] This is an important aspect in the debate of presymptomatic case-finding.

The BMJ's Too Much Medicine campaign aims to highlight the threat to human health posed by overdiagnosis and the waste of resources on unnecessary care (http://www.bmj.com/too-much-medicine). American data have shown that the majority of all health care includes preference-sensitive and supply-sensitive services. The extent of these services varies greatly without necessarily leading to better health.[42 43] A great deal of activity in such services is based on identifying subclinical disease. The negative health effect this might have, modulated through reduced SRH, can explain why population mortality is not reduced in areas with high frequency of diagnoses.

Both public and academic debates are often characterised by the conviction that all medical treatment is efficient. Wennberg showed that a relatively small proportion of all medical treatment is indisputably good for health.[44] The concern for unrevealed risk factors and subclinical conditions might lead to unnecessary costly health care interventions, increase

For peer review only

supply-sensitive services without positive health effect and have a negative influence on people's general and self-perceived health causing more harm than good.[15]

Of ethical reasons, the possible causal effect of disease labelling on SRH is impossible to assess in a randomized controlled trial. Our study emphasizes the need for more prospective research to investigate potential health effects of disease labelling and early diagnosis.

CONCLUSION

Our data suggest that early identification of disease may imply a negative effect on SRH and thereby eventually cause more harm than good. On a population level, such efforts might increase the costs in health care and the proportion of supply-sensitive services without any documented positive health effect. More research is needed concerning the possible adverse effects of disease labelling on SRH and mortality.

FOOTNOTES

Copyright The Corresponding Author has the right to grant on behalf of all authors and does grant on behalf of all authors, a worldwide licence to the Publishers and its licensees in perpetuity, in all forms, formats and media (whether known now or created in the future), to i) publish, reproduce, distribute, display and store the Contribution, ii) translate the Contribution into other languages, create adaptations, reprints, include within collections and create summaries, extracts and/or, abstracts of the Contribution, iii) create any other derivative work(s) based on the Contribution, iv) to exploit all subsidiary rights in the Contribution, v) the inclusion of electronic links from the Contribution to third party material where-ever it may be located; and, vi) licence any third party to do any or all of the above.

Competing interests All authors have completed the ICMJE uniform disclosure form at www.icmje.org/coi_disclosure.pdf and declare: no support from any organisation for the submitted work; no financial relationships with any organisations that might have an interest in the submitted work in the previous three years; no other relationships or activities that could appear to have influenced the submitted work.

Contributors All authors meet the four criteria for authorship recommended by the International Committee of Medical Journal Editors. SF, AL, and SK have been active supervisors in study conception, design, conduct, interpretation, and reporting. PJ analyzed the data and drafted the manuscript. Critical revisions were done by all supervisors and all authors approved the final version of the manuscript. SF, AL, SK and PJ are joint guarantors.

For peer review only

Acknowledgements The Nord-Trøndelag Health Study (The HUNT Study) is a collaboration between HUNT Research Centre (Faculty of Medicine, Norwegian University of Science and Technology NTNU), Nord-Trøndelag County Council, Central Norway Health Authority, and the Norwegian Institute of Public Health. The authors would like to thank Bjørn Olav Åsvold for his contribution in planning of the thyroid-part of the study.

Transparency declaration PJ affirms that this manuscript is an honest, accurate, and transparent account of the study being reported; that no important aspects of the study have been omitted; and that there were no discrepancies from the study as planned.

Ethical approval The study was approved by the Regional Committee for Medical Research. All participants signed written informed consent.

Funding No external funding. PJ received a PhD-grant, funded by the Norwegian University of Science and Technology, NTNU.

Data sharing statement No additional data available.

For peer review only

1. Ziemer DC, Miller CD, Rhee MK, et al. Clinical inertia contributes to poor diabetes control in a primary care setting. Diabetes Educ 2005;**31**(4):564-71 doi: 10.1177/0145721705279050[published Online First: Epub Date]|.
2. Borzecki AM, Wong AT, Hickey EC, et al. Hypertension control: how well are we doing? Arch Intern Med 2003;**163**(22):2705-11 doi: 10.1001/archinte.163.22.2705[published Online First: Epub Date]|.
3. Andros V, Egger A, Dua U. Blood pressure goal attainment according to JNC 7 guidelines and utilization of antihypertensive drug therapy in MCO patients with type 1 or type 2 diabetes. J Manag Care Pharm 2006;**12**(4):303-9
4. Liddy C, Singh J, Hogg W, et al. Quality of cardiovascular disease care in Ontario, Canada: missed opportunities for prevention - a cross sectional study. BMC Cardiovasc Disord 2012;**12**:74 doi: 10.1186/1471-2261-12-74[published Online First: Epub Date]|.
5. Huebschmann AG, Mizrahi T, Soenksen A, et al. Reducing clinical inertia in hypertension treatment: a pragmatic randomized controlled trial. J Clin Hypertens (Greenwich) 2012;**14**(5):322-9 doi: 10.1111/j.1751-7176.2012.00607.x[published Online First: Epub Date]|.
6. Alkerwi Aa, Pagny S, Lair M-L, et al. Level of Unawareness and Management of Diabetes, Hypertension, and Dyslipidemia among Adults in Luxembourg: Findings from ORISCAV-LUX Study. PLoS One 2013;**8**(3):e57920 doi: 10.1371/journal.pone.0057920[published Online First: Epub Date]|.
7. Khan N, Chockalingam A, Campbell NR. Lack of control of high blood pressure and treatment recommendations in Canada. Can J Cardiol 2002;**18**(6):657-61
8. Hetlevik I, Holmen J, Kruger O, et al. Fifteen years with clinical guidelines in the treatment of hypertension - still discrepancies between intentions and practice. Scand J Prim Health Care 1997;**15**(3):134-40 doi: Doi 10.3109/02813439709018503[published Online First: Epub Date]|.
9. Hetlevik I, Holmen J, Midthjell K. Treatment of diabetes mellitus - physicians' adherence to clinical guidelines in Norway. Scand J Prim Health Care 1997;**15**(4):193-97 doi: Doi 10.3109/02813439709035027[published Online First: Epub Date]|.
10. Petursson H, Getz L, Sigurdsson JA, et al. Current European guidelines for management of arterial hypertension: Are they adequate for use in primary care? Modelling study based on the Norwegian HUNT 2 population. BMC Fam Pract 2009;**10** doi: Artn 70 Doi 10.1186/1471-2296-10-70[published Online First: Epub Date]|.
11. Petursson H, Getz L, Sigurdsson JA, et al. Can individuals with a significant risk for cardiovascular disease be adequately identified by combination of several risk factors? Modelling study based on the Norwegian HUNT 2 population. J Eval Clin Pract 2009;**15**(1):103-09 doi: DOI 10.1111/j.1365-2753.2008.00962.x[published Online First: Epub Date]|.
12. Getz L, Sigurdsson JA, Hetlevik I, et al. Estimating the high risk group for cardiovascular disease in the Norwegian HUNT 2 population according to the 2003 European guidelines: modelling study. Br Med J 2005;**331**(7516):551-54A doi: DOI 10.1136/bmj.38555.648623.8F[published Online First: Epub Date]|.
13. Cabana MD, Rand CS, Powe NR, et al. Why don't physicians follow clinical practice guidelines?: A framework for improvement. JAMA 1999;**282**(15):1458-65 doi: 10.1001/jama.282.15.1458[published Online First: Epub Date]|.
14. Smith CM. Origin and uses of primum non nocere--above all, do no harm! J Clin Pharmacol 2005;**45**(4):371-7 doi: 10.1177/0091270004273680[published Online First: Epub Date]|.
15. Moynihan R, Doust J, Henry D. Preventing overdiagnosis: how to stop harming the healthy. Br Med J 2012;**344** doi: Artn E3502 Doi 10.1136/Bmj.E3502[published Online First: Epub Date]|.
16. Miilunpalo S, Vuori I, Oja P, et al. Self-rated health status as a health measure: the predictive value of self-reported health status on the use of physician services and on mortality in the

For peer review only

working-age population. J Clin Epidemiol 1997;**50**(5):517-28 doi: S0895-4356(97)00045-0 [pii][published Online First: Epub Date]|.
17. Meurer LN, Layde PM, Guse CE. Self-rated health status: a new vital sign for primary care? WMJ 2001;**100**(7):35-9
18. Bowling A. Just one question: If one question works, why ask several? J Epidemiol Community Health 2005;**59**(5):342-5 doi: 59/5/342 [pii] 10.1136/jech.2004.021204[published Online First: Epub Date]|.
19. Lundberg O, Manderbacka K. Assessing reliability of a measure of self-rated health. Scand J Soc Med 1996;**24**(3):218-24
20. Bopp M, Braun J, Gutzwiller F, et al. Health risk or resource? Gradual and independent association between self-rated health and mortality persists over 30 years. PLoS One 2012;**7**(2):e30795 doi: 10.1371/journal.pone.0030795 PONE-D-11-20499[pii][published Online First: Epub Date]|.
21. Moller L, Kristensen TS, Hollnagel H. Self rated health as a predictor of coronary heart disease in Copenhagen, Denmark. J Epidemiol Community Health 1996;**50**(4):423-8
22. Kaplan GA, Goldberg DE, Everson SA, et al. Perceived health status and morbidity and mortality: Evidence from the Kuopio Ischaemic Heart Disease Risk Factor Study. Int J Epidemiol 1996;**25**(2):259-65 doi: Doi 10.1093/Ije/25.2.259[published Online First: Epub Date]|.
23. Krokstad S, Johnsen R, Westin S. Social determinants of disability pension: a 10-year follow-up of 62 000 people in a Norwegian county population. Int J Epidemiol 2002;**31**(6):1183-91 doi: 10.1093/ije/31.6.1183[published Online First: Epub Date]|.
24. Halford C, Wallman T, Welin L, et al. Effects of self-rated health on sick leave, disability pension, hospital admissions and mortality. A population-based longitudinal study of nearly 15,000 observations among Swedish women and men. BMC Public Health 2012;**12**:1103 doi: 10.1186/1471-2458-12-1103[published Online First: Epub Date]|.
25. Latham K, Peek CW. Self-Rated Health and Morbidity Onset Among Late Midlife US Adults. J Gerontol B-Psychol 2013;**68**(1):107-16 doi: DOI 10.1093/geronb/gbs104[published Online First: Epub Date]|.
26. Benyamini Y, Idler EL. Community studies reporting association between self-rated health and mortality - Additional studies, 1995 to 1998. Res Aging 1999;**21**(3):392-401 doi: Doi 10.1177/0164027599213002[published Online First: Epub Date]|.
27. Nielsen AB, Siersma V, Hiort LC, et al. Self-rated general health among 40-year-old Danes and its association with all-cause mortality at 10-, 20-, and 29 years' follow-up. Scand J Public Health 2008;**36**(1):3-11 doi: 10.1177/1403494807085242[published Online First: Epub Date]|.
28. Barger SD, Muldoon MF. Hypertension labelling was associated with poorer self-rated health in the Third US National Health and Nutrition Examination Survey. J Hum Hypertens 2006;**20**(2):117-23 doi: 10.1038/sj.jhh.1001950[published Online First: Epub Date]|.
29. Bloom JR, Monterossa S. Hypertension Labeling and Sense of Well-Being. Am J Public Health 1981;**71**(11):1228-32 doi: Doi 10.2105/Ajph.71.11.1228[published Online First: Epub Date]|.
30. Moum T, Naess S, Sorensen T, et al. Hypertension Labeling, Life Events and Psychological Well-Being. Psychol Med 1990;**20**(3):635-46
31. Macdonald LA, Sackett DL, Haynes RB, et al. Labelling in hypertension: A review of the behavioural and psychological consequences. J Chronic Dis 1984;**37**(12):933-42 doi: http://dx.doi.org/10.1016/0021-9681(84)90070-5[published Online First: Epub Date]|.
32. Bianchi GP, Zaccheroni V, Solaroli E, et al. Health-related quality of life in patients with thyroid disorders - A study based on Short-Form 36 and Nottingham Health Profile Questionnaires. Qual Life Res 2004;**13**(1):45-54 doi: Doi 10.1023/B:Qure.0000015315.35184.66[published Online First: Epub Date]|.
33. Holmen J, Midthjell K, Krüger Ø, et al. The Nord-Trøndelag Health Study 1995-97 (HUNT 2): objectives, methods, and participation. Norsk Epidemiol 2003 **13** (1):19-32

For peer review only

34. Krokstad S, Langhammer A, Hveem K, et al. Cohort Profile: The HUNT Study, Norway. Int J Epidemiol 2012 doi: dys095 [pii] 10.1093/ije/dys095[published Online First: Epub Date]|.
35. Bjoro T, Holmen J, Kruger O, et al. Prevalence of thyroid disease, thyroid dysfunction and thyroid peroxidase antibodies in a large, unselected population. The Health Study of Nord-Trondelag (HUNT). Eur J Endocrinol 2000;**143**(5):639-47 doi: 1430639 [pii][published Online First: Epub Date]|.
36. Giron P. Determinants of self-rated health in Spain: differences by age groups for adults. Eur J Public Health 2012;**22**(1):36-40 doi: 10.1093/eurpub/ckq133[published Online First: Epub Date]|.
37. Davies HTO, Crombie IK, Tavakoli M. When can odds ratios mislead? Br Med J 1998;**316**(7136):989-91
38. Langhammer A, Krokstad S, Romundstad P, et al. The HUNT study: participation is associated with survival and depends on socioeconomic status, diseases and symptoms. BMC Med Res Methodol 2012;**12**:143 doi: 10.1186/1471-2288-12-143[published Online First: Epub Date]|.
39. Molarius A, Janson S. Self-rated health, chronic diseases, and symptoms among middle-aged and elderly men and women. J Clin Epidemiol 2002;**55**(4):364-70 doi: http://dx.doi.org/10.1016/S0895-4356(01)00491-7[published Online First: Epub Date]|.
40. Hansen AH, Halvorsen PA, Ringberg U, et al. Socio-economic inequalities in health care utilisation in Norway: a population based cross-sectional survey. BMC Health Serv Res 2012;**12**:336 doi: 10.1186/1472-6963-12-336[published Online First: Epub Date]|.
41. Vingilis E, Wade T, Seeley J. Predictors of adolescent health care utilization. J Adolesc 2007;**30**(5):773-800 doi: http://dx.doi.org/10.1016/j.adolescence.2006.10.001[published Online First: Epub Date]|.
42. Welch HG, Sharp SM, Gottlieb DJ, et al. Geographic variation in diagnosis frequency and risk of death among Medicare beneficiaries. JAMA 2011;**305**(11):1113-8 doi: 10.1001/jama.2011.307[published Online First: Epub Date]|.
43. Fisher ES, Wennberg DE, Stukel TA, et al. The implications of regional variations in Medicare spending. Part 2: health outcomes and satisfaction with care. Ann Intern Med 2003;**138**(4):288-98
44. Wennberg JE. *Tracking medicine. A researcher's quest to understand health care.* Oxford: Oxford University Press, 2010.

For peer review only

Table 1. Good self-rated health (SRH good) by sex and characteristics of the study population

**Study population (*n*= 33,734)**	**Women (*n*=17,514)**			**Men (*n*=16,220)**		
	n	%	SRH good (%)	*n*	%	SRH good (%)
Age group						
40-50 years	7058	40.3	77.6	6517	40.2	81.7
51-60 years	5709	32.6	64.4	5328	32.8	72.1
61-70 years	4747	27.1	56.6	4375	27.0	59.6
BMI (kg/m^2)						
<18,5	6821	0.6	58.4	4804	0.2	42.9
18,5-24,9	7026	39.1	72.8	8694	29.7	75.3
25,0-29,9	3489	40.3	68.5	2635	53.8	73.6
>30	104	20.0	56.5	35	16.3	64.8
(0.4% missing)						
Smoking status						
Never smoked daily	6973	40.1	70.3	4849	30.1	80.0
Previous daily smoker	4651	26.7	68.0	6105	37.9	69.8
Daily smoker	5763	33.1	64.3	5172	32.0	69.1
(0.7% missing)						
Alcohol use						
None to low intake	14888	88.7	66.7	11488	73.2	71.0
Moderate intake	1471	8.8	76.8	2839	18.1	78.7
High intake	430	2.6	76.3	1375	8.8	78.3
(3.7% missing)						
Educational level						
< 10 years	8133	48.5	60.5	5711	36.5	63.1
10 – 12 years	5682	33.9	72.8	6615	42.2	76.0
> 12 years	2962	17.7	80.0	3339	21.3	84.7
(3.8% missing)						
Employed						
Yes	11539	67.2	77.1	12309	77.1	79.9
No	5627	32.8	49.0	3665	22.9	49.2
(1.8% missing)						
Any long-term impairment						
No	10348	59.1	85.9	9912	63.0	88.7
Yes	6275	35.8	39.8	5829	37.0	46.0
(4.1% missing)						
Thyroid function						
No thyroid disease	14373	86.2	68.7	7804	94.3	72.1
Unknown hypothyroidism	107	0.6	78.5	16	0.2	68.8
Subclinical hypothyroidism	466	2.8	75.0	150	1.8	66.4
Known hypothyroidism	858	5.1	48.9	124	1.5	58.1
Other thyroid dysfunction	872	5.2	61.4	180	2.2	56.2
(1.3% missing)						
Diabetes mellitus						
No diabetes	11279	64.6	69.4	9196	56.9	74.6
Unknown diabetes	46	0.3	52.2	88	0.5	67.8
Probable diabetes	5728	32.8	65.9	6392	39.6	71.5
Known diabetes	395	2.3	43.1	482	3.0	49.5
(0.4% missing)						
Blood pressure status						
Normotensive	9135	52.6	72.4	6858	42.7	77.5
Unknown mild/moderate hypertension	4473	25.8	67.9	5343	33.3	74.7
Unknown severe hypertension	403	2.4	70.7	349	2.2	73.8
Known hypertension	3327	19.2	54.0	3498	21.8	59.6
(0.9% missing)						

For peer review only - http://bmjopen.bmj.com/site/about/guidelines.xhtml

Table 2. The association between self-rated health and thyroid function, diabetes mellitus and blood pressure. Odds ratio (OR) of good self-rated health and 95% confidence intervals (95% CI), crude and adjusted for age, other long-term illness or injury that impairs function in everyday life, smoking habits, alcohol use, educational level, work status and body mass index. Cases with missing data were excluded from the analyses.

	**Women**	**OR (95% CI)**		**Men**	**OR (95% CI)**	
	n	Crude	Adjusted	*n*	Crude	Adjusted
**Thyroid function**						
No thyroid dysfunction	12476	1.00	1.00	7045	1.00	1.00
Unknown hypothyroidism	92	1.66 (1.05-2.64)	1.84 (1.02-3.33)	14	0.85 (0.30-2.45)	1.28 (0.35-4.65)
Subclinical hypothyroidism	394	1.37 (1.10-1.69)	1.48 (1.13-1.94)	128	0.77 (0.54-1.08)	1.13 (0.72-1.76)
Known hypothyroidism	718	0.44 (0.38-0.50)	0.49 (0.41-0.59)	107	0.54 (0.37-0.77)	0.69 (0.44-1.09)
Other thyroid dysfunction	729	0.72 (0.63-0.83)	0.77 (0.65-0.93)	163	0.50 (0.37-0.67)	0.58 (0.41-0.84)
**Diabetes mellitus**						
No diabetes	9946	1.00	1.00	8464	1.00	1.00
Unknown diabetes	39	0.48 (0.27-0.86)	0.66 (0.32-1.37)	82	0.72 (0.46-1.13)	1.03 (0.59-1.81)
Probable diabetes	4797	0.85 (0.80-0.91)	1.01 (0.92-1.10)	5759	0.86 (0.80-0.92)	0.99 (0.91-1.08)
Known diabetes	318	0.33 (0.27-0.41)	0.53 (0.41-0.69)	395	0.33 (0.28-0.40)	0.55 (0.43-0.70)
**Blood pressure**						
No hypertension	8180	1.00	1.00	6378	1.00	1.00
Unknown mild/moderate hypertension	3787	0.81 (0.75-0.87)	1.10 (0.99-1.22)	4837	0.86 (0.79-0.93)	1.01 (0.92-1.12)
Unknown severe hypertension	325	0.92 (0.74-1.15)	1.52 (1.14-2.02)	308	0.85 (0.77-1.08)	1.48 (1.09-2.02)
Known hypertension	2745	0.45 (0.41-0.49)	0.69 (0.61-0.77)	3118	0.43 (0.39-0.47)	0.64 (0.57-0.72)

For peer review only

What is already known on this topic

Awareness of thyroid dysfunction, diabetes mellitus, and hypertension is associated with reduced self-rated health

Screening, increasingly sensitive diagnostic tests, and widened diagnostic criteria will define more healthy people as sick

What this study adds

Unawareness of hypothyroidism, diabetes mellitus, or hypertension is not associated with reduced self-rated health

For peer review only

STROBE 2007 (v4) Statement—Checklist of items that should be included in reports of *cross-sectional studies*

Section/Topic	Item #	Recommendation	Reported on page #
**Title and abstract**	1	(*a*) Indicate the study's design with a commonly used term in the title or the abstract	2
		(*b*) Provide in the abstract an informative and balanced summary of what was done and what was found	2
**Introduction**			
Background/rationale	2	Explain the scientific background and rationale for the investigation being reported	3-4
Objectives	3	State specific objectives, including any prespecified hypotheses	4
**Methods**			
Study design	4	Present key elements of study design early in the paper	2-3
Setting	5	Describe the setting, locations, and relevant dates, including periods of recruitment, exposure, follow-up, and data collection	5
Participants	6	(*a*) Give the eligibility criteria, and the sources and methods of selection of participants	5
Variables	7	Clearly define all outcomes, exposures, predictors, potential confounders, and effect modifiers. Give diagnostic criteria, if applicable	5-8
Data sources/ measurement	8*	For each variable of interest, give sources of data and details of methods of assessment (measurement). Describe comparability of assessment methods if there is more than one group	5-8
Bias	9	Describe any efforts to address potential sources of bias	7-8
Study size	10	Explain how the study size was arrived at	5
Quantitative variables	11	Explain how quantitative variables were handled in the analyses. If applicable, describe which groupings were chosen and why	5-8
Statistical methods	12	(*a*) Describe all statistical methods, including those used to control for confounding	7-8
		(*b*) Describe any methods used to examine subgroups and interactions	8
		(*c*) Explain how missing data were addressed	Table 2
		(*d*) If applicable, describe analytical methods taking account of sampling strategy	-
		(*e*) Describe any sensitivity analyses	-
**Results**			

For peer review only

Participants	13*	(a) Report numbers of individuals at each stage of study—eg numbers potentially eligible, examined for eligibility, confirmed eligible, included in the study, completing follow-up, and analysed	5
		(b) Give reasons for non-participation at each stage	5
		(c) Consider use of a flow diagram	-
Descriptive data	14*	(a) Give characteristics of study participants (eg demographic, clinical, social) and information on exposures and potential confounders	Table 1
		(b) Indicate number of participants with missing data for each variable of interest	Table 1
Outcome data	15*	Report numbers of outcome events or summary measures	Table 1
Main results	16	(*a*) Give unadjusted estimates and, if applicable, confounder-adjusted estimates and their precision (eg, 95% confidence interval). Make clear which confounders were adjusted for and why they were included	Table 2 Page 9-11
		(*b*) Report category boundaries when continuous variables were categorized	5-7
		(*c*) If relevant, consider translating estimates of relative risk into absolute risk for a meaningful time period	-
Other analyses	17	Report other analyses done—eg analyses of subgroups and interactions, and sensitivity analyses	10-11
**Discussion**			
Key results	18	Summarise key results with reference to study objectives	11
Limitations	19	Discuss limitations of the study, taking into account sources of potential bias or imprecision. Discuss both direction and magnitude of any potential bias	11-13
Interpretation	20	Give a cautious overall interpretation of results considering objectives, limitations, multiplicity of analyses, results from similar studies, and other relevant evidence	13-14
Generalisability	21	Discuss the generalisability (external validity) of the study results	11
**Other information**			
Funding	22	Give the source of funding and the role of the funders for the present study and, if applicable, for the original study on which the present article is based	15

*Give information separately for cases and controls in case-control studies and, if applicable, for exposed and unexposed groups in cohort and cross-sectional studies.

Note: An Explanation and Elaboration article discusses each checklist item and gives methodological background and published examples of transparent reporting. The STROBE checklist is best used in conjunction with this article (freely available on the Web sites of PLoS Medicine at http://www.plosmedicine.org/, Annals of Internal Medicine at http://www.annals.org/, and Epidemiology at http://www.epidem.com/). Information on the STROBE Initiative is available at www.strobe-statement.org.

For peer review only

Is there an association between disease ignorance and self-rated health? The HUNT Study, a cross-sectional survey

Journal:	*BMJ Open*
Manuscript ID:	bmjopen-2014-004962.R1
Article Type:	Research
Date Submitted by the Author:	28-Apr-2014
Complete List of Authors:	Jørgensen, Pål; Norwegian University of Science and Technology, Department of Public Health and General Practice<br>Langhammer, Arnulf; Norwegian University of Science and Technology, HUNT Research Centre<br>Krokstad, Steinar; Norwegian University of Science and Technology, HUNT Research Centre<br>Forsmo, Siri; Norwegian University of Science and Technology, Department of Public Health and General Practice
**Primary Subject Heading**:	General practice / Family practice
Secondary Subject Heading:	Epidemiology, Public health, Diabetes and endocrinology
Keywords:	Hypertension < CARDIOLOGY, General diabetes < DIABETES & ENDOCRINOLOGY, Thyroid disease < DIABETES & ENDOCRINOLOGY, PRIMARY CARE

SCHOLARONE™
Manuscripts

Is there an association between disease ignorance and self-rated health? The HUNT Study, a cross-sectional survey

Pål Jørgensen, Arnulf Langhammer, Steinar Krokstad, Siri Forsmo

Department of Public Health and General Practice, Norwegian University of Science and Technology, 7489 Trondheim, Norway Pål Jørgensen
PhD Candidate
HUNT Research Centre, Department of Public Health and General Practice, Norwegian University of Science and Technology, 7600 Levanger, Norway Arnulf Langhammer
Professor, Head of HUNT Databank
HUNT Research Centre, Department of Public Health and General Practice, Norwegian University of Science and Technology, Steinar Krokstad
Professor, Head of HUNT Research Center
Department of Public Health and General Practice, Norwegian University of Science and Technology, Siri Forsmo
Professor, Head of Department

Correspondence to: P Jørgensen pal_jorgensen@ntnu.no

Keywords: Self-rated health, awareness, hypothyroidism, diabetes, hypertension.

Word count: 3359

For peer review only

ABSTRACT

Objective To explore if awareness versus unawareness of thyroid dysfunction, diabetes mellitus or hypertension is associated with self-rated health.

Design Large-scale, cross sectional population based study. The association between thyroid function, diabetes mellitus, and blood pressure and self-rated health was explored by multiple logistic regression analysis.

Setting The second survey of the Nord-Trøndelag Health Study, HUNT2, 1995-97.

Participants 33,734 persons aged 40-70 years.

Primary outcome measures Logistic regression was used to estimate odds ratios for good self-rated health as a function of thyroid status, diabetes mellitus status and blood pressure status.

Results Persons aware of their hypothyroidism, diabetes mellitus, or hypertension reported poorer self-rated health than individuals without such conditions. Women with unknown and subclinical hypothyroidism reported better self-rated health than women with normal thyroid status. Both in women and men, unknown and probable diabetes, as well as unknown mild/moderate hypertension was not associated with poorer health. Further, persons with unknown severe hypertension reported better health than normotensive persons.

Conclusions People with undiagnosed, but prevalent hypothyroidism, diabetes mellitus, and hypertension often have good self-rated health, whilst when aware of their diagnoses, they report reduced self-rated health. Use of screening, more sensitive tests, and widened diagnostic criteria might have a negative effect on perceived health in the population.

For peer review only - http://bmjopen.bmj.com/site/about/guidelines.xhtml

STRENGTHS AND LIMITATIONS OF THIS STUDY

Strengths

- Sample from a large-scale, general population
- High-prevalent diseases under study; ensuring statistical power in subgroup analyses

Limitations

- Study mainly based on self-reported data
- Cross-sectional study; susceptibility to confounding and impossibility to assume causal relationships

INTRODUCTION

Guidelines for prevention and treatment have been developed for most high prevalent diseases in western countries aiming for reduction of morbidity and mortality by interventions mainly in primary health care (PHC).

In the society there seems to be an increasing conviction of achievable zero-vision regarding risks and diseases. Part of the strategy is to detect risk factors and pre-diseases in even earlier stages. From a secondary or tertiary health care level, this might seem reasonable since intervention on many individuals with specific risk factors presumably can prevent or delay disease or progression of disease. Further, health authorities and hospital clinicians regularly raise concern of the lack of detection of risk factors, of subclinical conditions and of achieving treatment goals.[1-7] Norwegian studies have shown that guidelines are often difficult to implement and adhere to in PHC.[8 9] According to guidelines, most individuals would be defined as at risk and resources needed to handle this appropriately could destabilize the entire health care system.[10-12] An American review pointed out knowledge, attitude,

For peer review only

and behaviour as barriers of physician's adherence to clinical guidelines.[13] In an already complex and busy PHC-setting, one might expect that resources used for disease prevention and case finding have to compete with resources for handling acknowledged disease. Also, physicians might want to avoid increasing disease related burden for patients, in line with the old wisdom; "primum non nocere".[14] Thus the risk and disease zero-visions in society and among politicians are seldom shared by PHC professionals.

When guidelines, mainly based on research from high risk hospital populations, are applied on low risk populations in PHC, more healthy individuals are identified as being at risk or are given diagnoses. Also the widened inclusion criteria for diagnoses in general and use of more sensitive tests contribute to define more individuals at risk or as unhealthy.[15] Possible undesirable outcomes of such strategies remain unclear.

Self-rated health (SRH) is a valid and widely used measure of general health in epidemiologic research.[16-19] It is associated with several clinical conditions often seen in PHC, with recovery [20-23] and is found to predict morbidity, sick leave, and disability pension,[24 25] as well as mortality.[26-28] The majority of studies describing association between labelling of disease per se and SRH have focused on arterial hypertension.[29-32] However, one study has indicated reduced SRH among individuals labelled with thyroid dysfunctions.[33]

The aim of this study was to investigate whether persons' awareness versus unawareness of thyroid dysfunction, diabetes mellitus, or hypertension was associated with their SRH, as reported in a population-based health study in Norway.

For peer review only - http://bmjopen.bmj.com/site/about/guidelines.xhtml

METHODS

Study population

The data sample in this study stem from the second wave of the Nord-Trøndelag Health Study (HUNT2) conducted in 1995-97 in the county of Nord-Trøndelag, Norway. All individuals aged 20 years and older, living in the county, were invited (94,194 individuals). In all, 66.7% of men (n=30,860) and 75.5% of women (n=35,280) participated. The survey consisted of both questionnaires and measurements, and is described previously in detail.[34 35] In our study we included answers from the main questionnaire and the baseline measurements for persons aged 40-70 years. The age span was chosen because thyroid stimulating hormone (TSH) was analysed in all women and in 50% of men at this age, of a rather low disease burden in people younger than 40 years, and of a lower attendance rate under and above this age span. A total of 24,950 individuals had TSH measurements and answered thyroid questions, thus were eligible for analysis on thyroid dysfunction, whilst in the analysis of diabetes mellitus and blood pressure, 33,734 individuals were included in all.

Self-rated health

The first question in the main questionnaire in HUNT2, answered before attending the examination stations, was "How is your health at the moment?", with answer alternatives; "very good", "good", "not so good", and "poor". This short version of SRH measure is shown to be a valid predictor of mortality. [18 28 36 37] We dichotomized the answers into "good" (very good, good) and "poor" (not so good, poor). Dichotomization of multinomial SRH is commonly performed, and has been validated by Manor et. al.[38]

Thyroid function

The participants answered questions on history of hypo- and hyperthyroidism, goitre, other thyroid diseases, and treatment with thyroxin, radio-iodine, surgery, or thyreostatic medication.

Serum TSH and free T4 were analysed at the Hormone laboratory, Aker University Hospital, Norway. The laboratory reference value for TSH, as defined prior to the survey, was 0.2-4.5 mU/L and for free T_4 8.0-20.0 pmol/L. If TSH was <0.2 mU/L or >4.0 mU/L, and/or if the participant reported any thyroid disease, serum free T_4 was also measured.[39]

Individuals reporting no previous thyroid disease and having TSH within reference range were categorized as "no thyroid disease" and chosen as reference category. No previous thyroid disease combined with TSH >4.5 mU/L and free T_4 <8.0 pmol/L was defined as unknown hypothyroidism. No previous thyroid disease combined with TSH >4.5 mU/L and free T_4 8.0 – 20.0 pmol/L was defined as subclinical hypothyroidism. Individuals reporting hypothyroidism and use of thyroxin were classified as having known hypothyroidism, regardless of the TSH and T4 levels. Affirmative answers to other thyroid related questions or measures outside reference range in the remainders were classified as other thyroid dysfunction.

Diabetes mellitus

Diabetes mellitus was assessed through self-report and blood samples. Serum glucose was analysed at Levanger Hospital, Norway. Those reporting no diabetes and having normal glucose levels (<5.5 mmol/L) were classified as "no diabetes" and were chosen as reference category. No self-reported diabetes and non-fasting glucose >11.0 mmol/L was categorized as unknown diabetes, whereas no diabetes and non-fasting glucose 5.5-11.0 mmol/L was

For peer review only

For peer review only - http://bmjopen.bmj.com/site/about/guidelines.xhtml

categorized as probable diabetes. Self-reported diabetes was classified as known diabetes regardless of the glucose level.

Blood pressure

In the questionnaire participants were asked about the doctor's advice after the latest blood pressure measurement prior to participation in HUNT. The answer categories were: "no follow-up and no medication necessary", "recommended follow-up examination but not to take medicine", "start or continue taking medicine for high blood pressure", or "never measured". At HUNT2, mean systolic and mean diastolic arterial blood pressure (BP) of measurement 2 and 3 was categorized into normal (systolic (s) BP < 140 mmHg and diastolic (d) BP < 90 mmHg), mild hypertension (sBP 140-159 mmHg and dBP <100 mmHg or sBP <160 mmHg and dBP 90-99 mmHg), moderate hypertension (sBP 160-179 mmHg and dBP <109 mmHg or sBP <180 mmHg and dBP 100-109 mmHg), and severe hypertension (sBP>180 mmHg or dBP>110 mmHg). We constructed a new variable to define normotensive (reference), unknown mild- and moderate hypertensive, unknown severe hypertensive, and known hypertensive persons on the basis of self-report and measures.

Statistical analysis

The descriptive analyses of the study population were stratified by gender, and we used chi-square tests to examine any difference in proportions of SRH between the independent variables. Gender-stratified multiple logistic regression were used to estimate odds ratio (OR) with 95% confidence intervals (CI) for good SRH, as a function of thyroid status, diabetes mellitus status and blood pressure status, in separate unadjusted, age adjusted, and multi adjusted analyses for each condition.

For peer review only

Age, smoking, alcohol consumption, body mass index (BMI), working- and educational status, and self-reported limiting long-term illness or injury are associated with SRH[40] and the diseases under study, but likely not affected by SRH or the diseases. Hence, these variables were included, a priori, as confounders in the models. Age was categorized into age groups; 40-49 years, 50-59 years, and 60-70 years. Smoking status was categorized into never smoked daily, previous daily smoker, and current daily smoker. Alcohol units (AU) were defined as number of glasses of wine, beer or liquor. Those reporting to be teetotalers or to have alcohol intake less than four times a month or less than seven AU per two weeks were categorized as low consumers, those reporting drinking five to eight times a month or 8-14 AU per two weeks as moderate consumers, and those drinking more often than eight times a month or more than 14 AU as heavy consumers. BMI ($kg/m^{2)}$ was calculated of measured height and weight and categorized according to The World Health Organization's (WHO) definition; underweight (18.5 kg/m^2), normal weight (18.5-24.9 kg/m^2), overweight (25.0-29.9 kg/m^2) and obese (>30.0 kg/m^2). People reporting paid- or self-employed work were classified as working, otherwise as not working. Educational level was categorized into <10 years, 10-12 years and >12 years. We chose affirmative answer to the question "Do you suffer from any long-term illness or injury (at least one year) of a physical or psychological nature that impairs your functioning in your everyday life?" to represent all relevant chronic medical conditions that could confound the results.

To examine whether the association of the three disease statuses with SRH differed by categories of the other independent variables, we used likelihood ratio-tests with p-value for statistical interaction. We tested for multicollinearity between the independent variables, by linear regression.

For peer review only

In an additional analysis, the association between SRH and having had one or more medical consultations during the last year was investigated by logistic regression models, stratified by gender, both in the total study population and after exclusion of persons with diagnoses under study.

All analyses were preformed with IBM SPSS Statistics version 20 for windows.

The study was approved by the Regional Committee for Medical Research. All participants signed written informed consent.

RESULTS

In all age categories, a higher proportion of men than women reported good SRH ($p<0.001$), and in both sexes the proportion reporting good SRH declined by age ($p<0.002$) (Table 1). The proportion reporting good SRH was lower in overweight, obese, and underweight women, than in normal weight women ($p<0.001$). In men the proportion reporting good SRH declined between the normal weight group to overweight-, obese-, and underweight group ($p<0.001$). In previous and current female smokers, a lower proportion reported good SRH compared to non smokers ($p<0.001$). In men, the proportion reporting good SRH was higher among non smokers than among previous and current smokers ($p<0.001$), whereas there was no difference between previous and current smokers. The proportion reporting good SRH was higher in moderate and heavy alcohol consumers than in low consumers, increased with education and was higher for participants in paid work ($p<0.001$). Underweight men and persons with "any long-term impairment" had the lowest proportion reporting good SRH of all groups. The proportion reporting good SRH in the overall HUNT 2 study population differed from the proportions reported in persons without thyroid disease and normotensive persons ($p<0.01$), but not from persons without diabetes mellitus. However, the absolute differences were small.

For peer review only

There was no multicollinearity between the independent variables.

Thyroid function

Thyroid dysfunctions, both known and unknown were more often observed among women than among men (p<0.001) (Table 1). Women with known hypothyroidism had lower odds of reporting good SRH (OR 0.49 (95% CI 0.41 to 0.59) compared to women without thyroid disease in the adjusted analyses, but women with unknown or subclinical hypothyroidism had 84% and 48% higher odds, respectively, of reporting good SRH compared to the odds of women without thyroid disease (Table 2). The association between thyroid function and SRH was basically unchanged after inclusion of confounder variables. Corresponding, but non-significant associations were found among men.

Diabetes mellitus

The prevalence of unknown, probable, and known diabetes was slightly higher in men than in women (p<0.001) (Table 1). Women with known diabetes mellitus had lower odds of good SRH than those without diabetes in the adjusted analyses; OR 0.53 (95% CI 0.41 to 0.69), whereas in women with unknown or possible diabetes mellitus, the odds of good SRH were similar to the odds among persons without diabetes, in the adjusted analyses (Table 2). In the adjusted analyses, the association between unknown and probable diabetes mellitus with poor SRH, found in the crude analyses, disappeared when age was included in the model in women and men, but also by inclusion of working status alone in women. In men, the association of diabetes status with SRH differed by levels of education. Among men without higher education (12 years or less), the odds of good SRH were as in the main-effect model (Table 2). However in men with higher education, the ORs of good SRH were barely significantly lower among men with unknown diabetes (OR 0.29 (95% CI 0.09-1.00)) and among men with

For peer review only

possible diabetes (OR 0.79 (95% CI 0.63-1.00)) compared to men without diabetes. Among men with known diabetes in this stratum, the OR of good SRH was 0.31 (95% CI 0.17-0.54) compared to men without diabetes.

Blood pressure

The prevalence of unknown mild and moderate hypertension was higher in men than in women ($p<0.001$). Unknown severe hypertension and known hypertension were equally distributed between women and men (Table 1). Women with known hypertension had lower odds of reporting good SRH than normotensive women, in the adjusted analyses. The figures were similar in men (Table 2). In contrast; compared to normotensive women, those with unknown severe hypertension had 52 % higher odds of reporting good SRH, with similar figures in men. Persons with unknown mild and moderate hypertension reported good SRH similar to the normotensive ones. Adjusted for age, the association between unknown mild and moderate hypertension and poor SRH disappeared, simultaneously; unknown severe hypertension became associated with good SRH in women. In men, age had to be added along with either education- or working status to achieve the latter association. **Additional analyses**

Women with poor SRH had more than six times the odds of those with good SRH to have had a medical consultation during the last year; OR 6.29 (95% CI 5.47 to 7.22). For men the corresponding OR was 5.53 (95% CI 4.86 to 6.29). After exclusion of persons with diagnosed thyroid disease, diabetes mellitus, known hypertension, and “any long-term disease”, the corresponding OR was 3.71 (95% CI 2.90 to 4.73), with similar figures in men.

DISCUSSION

This large population-based study showed that persons with known thyroid dysfunction, diabetes mellitus, and hypertension were less likely to report good SRH than those without

For peer review only

such conditions. Less expected, persons with unknown and subclinical hypothyroidism were more likely to report good SRH than those without thyroid disease. Similarly, those with unknown severe hypertension were more likely to report good SRH, compared to persons with normal blood pressure. In general, persons with unknown diabetes and unknown mild/moderate hypertension, reported good health, just like the reference group.

In general, of the confounders, age seemed to influence the association between disease statuses and SRH most when adjusted for. Age was found to explain the association of poor SRH with unknown and probable diabetes, and with unknown mild and moderate hypertension. In women, age even contributed to an association between unknown severe hypertension and good SRH. There seemed to be a linear decrease by age categories in the association with good SRH. The way age is known to be related to both disease and SRH makes these findings reasonable.

Although both a qualitative and quantitative measure of association, the estimated ORs in our study will, if interpreted as relative risks, overestimate the case, because the prevalence of good SRH is high in all groups.[41]

The main strengths of this study were the numbers of participants, that the diseases we studied were high-prevalent, and that we could assume representativeness to the general population regarding the variables included. It is known that individuals with high burden of symptoms are less likely to attend surveys, but so far there has been little evidence that non-participation on this basis introduce substantial bias in associational studies.[42] We studied diseases with generally low symptom-burden, and expect selection bias to be negligible.

The main limitation was possibly that we relied on self-reported data on both dependent and independent variables. The validity of self reported measures relevant for this study is

For peer review only

questionable. However, if any, there should be a non-differential misclassification, that only should underestimate the associations found.

There is always a possibility of residual confounding in non-randomized study designs, and due to the observational, cross-sectional design we neither can assume a causal relationship.

Bias due to differential detection of disease in the study population could lead to a type 1 error. Hypothyroidism and diabetes mellitus are often associated with vague symptoms such as tiredness and weakness. These symptoms are strongly associated with reduced SRH.[43] It is likely that presenting such symptoms for the GP would result in measurements of TSH, free T4, and serum glucose, thus reveal any related dysfunction. Low self-perceived health increases the probability to visit a GP.[44] The social security covers most costs related to clinical measurements and blood sampling in Norway, thus we expect GPs to have a low threshold to measure thyroid function, blood pressure or glucose levels. People who do not consult their GP will not have diseases with mild or no symptoms diagnosed, and their personality could be characterized by less worrying and more optimistic attitude to health being reflected in better SRH.[45]

On the other hand, the association between known disease and poor SRH could in fact be explained physiologically due to the pathological effect of disease. Lack of corresponding association between ignored, but prevalent disease and poor SRH is not in line with this hypothesis, rising question of a possible adverse effect of disease labelling. However, confounding by severity of disease cannot be ruled out.

Due to low numbers of persons having unknown hypothyroidism and diabetes mellitus, the results should be interpreted with caution. On the other hand, there were high numbers in the

For peer review only

subclinical and probable groups, and analyses of these groups did not show any associations with poor SRH, also rising question of a possible disease labelling effect.

The fact that the HUNT2 survey was carried out nearly 20 years ago raises a question of generalisability to today's population. Stability of SRH over time has not been investigated in our study population, although investigated among adolescents.[46] We do not expect the association between low burden- or subclinical disease with SRH to be time dependent to the extent that it would change our results considerably. Neither do we expect the changes in prevalence of most explanatory variables to influence the associations found.

Consistent findings regarding unknown, subclinical, and probable disease, versus known disease, could indicate a potential adverse effect of disease labelling. Although early detection of disease is protective on morbidity and mortality for many diseases, low SRH is also shown to be associated morbidity and mortality.[25-27] This is an important aspect in the debate of presymptomatic case-finding.

The BMJ's Too Much Medicine campaign aims to highlight the threat to human health posed by overdiagnosis and the waste of resources on unnecessary care (http://www.bmj.com/too-much-medicine). American data have shown that the majority of all health care includes preference-sensitive and supply-sensitive services. The extent of these services varies greatly without necessarily leading to better health.[47 48] A great deal of activity in such services is based on identifying subclinical disease. The negative health effect this might have, modulated through reduced SRH, can explain why population mortality is not reduced in areas with high frequency of diagnoses.

Both public and academic debates are often characterised by the conviction that all medical treatment is efficient. Wennberg showed that a relatively small proportion of all medical

For peer review only

treatment is indisputably good for health.[49] The concern for unrevealed risk factors and subclinical conditions might lead to unnecessary costly health care interventions, increase supply-sensitive services without positive health effect and have a negative influence on people's general and self-perceived health causing more harm than good.[15]

Of ethical reasons, the possible causal effect of disease labelling on SRH is impossible to assess in a randomized controlled trial. Our study emphasizes the need for more prospective research to investigate potential health effects of disease labelling and early diagnosis.

CONCLUSION

Our data suggest that early identification of disease may imply a negative effect on SRH, and to the extent that SRH has been associated with greater mortality, this may lead to harm. However, as it is also known that diseases such as diabetes and hypertension also lead to increased mortality when detected after cardiovascular and metabolic complications have developed, it remains to be seen if an early identification or a late detection strategy would provide optimal health for the population. **FOOTNOTES**

Copyright The Corresponding Author has the right to grant on behalf of all authors and does grant on behalf of all authors, a worldwide licence to the Publishers and its licensees in perpetuity, in all forms, formats and media (whether known now or created in the future), to i) publish, reproduce, distribute, display and store the Contribution, ii) translate the Contribution into other languages, create adaptations, reprints, include within collections and create summaries, extracts and/or, abstracts of the Contribution, iii) create any other derivative work(s) based on the Contribution, iv) to exploit all subsidiary rights in the Contribution, v) the inclusion of electronic links from the Contribution to third party material where-ever it may be located; and, vi) licence any third party to do any or all of the above.

Competing interests All authors have completed the ICMJE uniform disclosure form at www.icmje.org/coi_disclosure.pdf and declare: no support from any organisation for the submitted work; no financial relationships with any organisations that might have an interest in the submitted work in the previous three years; no other relationships or activities that could appear to have influenced the submitted work.

For peer review only

Contributors All authors meet the four criteria for authorship recommended by the International Committee of Medical Journal Editors. SF, AL, and SK have been active supervisors in study conception, design, conduct, interpretation, and reporting. PJ analyzed the data and drafted the manuscript. Critical revisions were done by all supervisors and all authors approved the final version of the manuscript. SF, AL, SK and PJ are joint guarantors.

Acknowledgements The Nord-Trøndelag Health Study (The HUNT Study) is a collaboration between HUNT Research Centre (Faculty of Medicine, Norwegian University of Science and Technology NTNU), Nord-Trøndelag County Council, Central Norway Health Authority, and the Norwegian Institute of Public Health. The authors would like to thank Bjørn Olav Åsvold for his contribution in planning of the thyroid-part of the study.

Transparency declaration PJ affirms that this manuscript is an honest, accurate, and transparent account of the study being reported; that no important aspects of the study have been omitted; and that there were no discrepancies from the study as planned.

Ethical approval The study was approved by the Regional Committee for Medical Research. All participants signed written informed consent.

Funding No external funding. PJ received a PhD-grant, funded by the Norwegian University of Science and Technology, NTNU.

Data sharing statement No additional data available.

For peer review only

1. Ziemer DC, Miller CD, Rhee MK, et al. Clinical inertia contributes to poor diabetes control in a primary care setting. Diabetes Educ 2005;**31**(4):564-71 doi: 10.1177/0145721705279050[published Online First: Epub Date]|.
2. Borzecki AM, Wong AT, Hickey EC, et al. Hypertension control: how well are we doing? Arch Intern Med 2003;**163**(22):2705-11 doi: 10.1001/archinte.163.22.2705[published Online First: Epub Date]|.
3. Andros V, Egger A, Dua U. Blood pressure goal attainment according to JNC 7 guidelines and utilization of antihypertensive drug therapy in MCO patients with type 1 or type 2 diabetes. J Manag Care Pharm 2006;**12**(4):303-9
4. Liddy C, Singh J, Hogg W, et al. Quality of cardiovascular disease care in Ontario, Canada: missed opportunities for prevention - a cross sectional study. BMC Cardiovasc Disord 2012;**12**:74 doi: 10.1186/1471-2261-12-74[published Online First: Epub Date]|.
5. Huebschmann AG, Mizrahi T, Soenksen A, et al. Reducing clinical inertia in hypertension treatment: a pragmatic randomized controlled trial. J Clin Hypertens (Greenwich) 2012;**14**(5):322-9 doi: 10.1111/j.1751-7176.2012.00607.x[published Online First: Epub Date]|.
6. Alkerwi Aa, Pagny S, Lair M-L, et al. Level of Unawareness and Management of Diabetes, Hypertension, and Dyslipidemia among Adults in Luxembourg: Findings from ORISCAV-LUX Study. PLoS One 2013;**8**(3):e57920 doi: 10.1371/journal.pone.0057920[published Online First: Epub Date]|.
7. Khan N, Chockalingam A, Campbell NR. Lack of control of high blood pressure and treatment recommendations in Canada. Can J Cardiol 2002;**18**(6):657-61
8. Hetlevik I, Holmen J, Kruger O, et al. Fifteen years with clinical guidelines in the treatment of hypertension - still discrepancies between intentions and practice. Scand J Prim Health Care 1997;**15**(3):134-40 doi: Doi 10.3109/02813439709018503[published Online First: Epub Date]|.
9. Hetlevik I, Holmen J, Midthjell K. Treatment of diabetes mellitus - physicians' adherence to clinical guidelines in Norway. Scand J Prim Health Care 1997;**15**(4):193-97 doi: Doi 10.3109/02813439709035027[published Online First: Epub Date]|.
10. Petursson H, Getz L, Sigurdsson JA, et al. Current European guidelines for management of arterial hypertension: Are they adequate for use in primary care? Modelling study based on the Norwegian HUNT 2 population. BMC Fam Pract 2009;**10** doi: Artn 70 Doi 10.1186/1471-2296-10-70[published Online First: Epub Date]|.
11. Petursson H, Getz L, Sigurdsson JA, et al. Can individuals with a significant risk for cardiovascular disease be adequately identified by combination of several risk factors? Modelling study based on the Norwegian HUNT 2 population. J Eval Clin Pract 2009;**15**(1):103-09 doi: DOI 10.1111/j.1365-2753.2008.00962.x[published Online First: Epub Date]|.
12. Getz L, Sigurdsson JA, Hetlevik I, et al. Estimating the high risk group for cardiovascular disease in the Norwegian HUNT 2 population according to the 2003 European guidelines: modelling study. Br Med J 2005;**331**(7516):551-54A doi: DOI 10.1136/bmj.38555.648623.8F[published Online First: Epub Date]|.
13. Cabana MD, Rand CS, Powe NR, et al. Why don't physicians follow clinical practice guidelines?: A framework for improvement. JAMA 1999;**282**(15):1458-65 doi: 10.1001/jama.282.15.1458[published Online First: Epub Date]|.
14. Smith CM. Origin and uses of primum non nocere--above all, do no harm! J Clin Pharmacol 2005;**45**(4):371-7 doi: 10.1177/0091270004273680[published Online First: Epub Date]|.
15. Moynihan R, Doust J, Henry D. Preventing overdiagnosis: how to stop harming the healthy. Br Med J 2012;**344** doi: Artn E3502 Doi 10.1136/Bmj.E3502[published Online First: Epub Date]|.
16. Miilunpalo S, Vuori I, Oja P, et al. Self-rated health status as a health measure: the predictive value of self-reported health status on the use of physician services and on mortality in the

For peer review only

working-age population. J Clin Epidemiol 1997;**50**(5):517-28 doi: S0895-4356(97)00045-0 [pii][published Online First: Epub Date]|.
17. Meurer LN, Layde PM, Guse CE. Self-rated health status: a new vital sign for primary care? WMJ 2001;**100**(7):35-9
18. Bowling A. Just one question: If one question works, why ask several? J Epidemiol Community Health 2005;**59**(5):342-5 doi: 59/5/342 [pii] 10.1136/jech.2004.021204[published Online First: Epub Date]|.
19. Lundberg O, Manderbacka K. Assessing reliability of a measure of self-rated health. Scand J Soc Med 1996;**24**(3):218-24
20. Bopp M, Braun J, Gutzwiller F, et al. Health risk or resource? Gradual and independent association between self-rated health and mortality persists over 30 years. PLoS One 2012;**7**(2):e30795 doi: 10.1371/journal.pone.0030795 PONE-D-11-20499[pii][published Online First: Epub Date]|.
21. Moller L, Kristensen TS, Hollnagel H. Self rated health as a predictor of coronary heart disease in Copenhagen, Denmark. J Epidemiol Community Health 1996;**50**(4):423-8
22. Kaplan GA, Goldberg DE, Everson SA, et al. Perceived health status and morbidity and mortality: Evidence from the Kuopio Ischaemic Heart Disease Risk Factor Study. Int J Epidemiol 1996;**25**(2):259-65 doi: Doi 10.1093/Ije/25.2.259[published Online First: Epub Date]|.
23. Krokstad S, Johnsen R, Westin S. Social determinants of disability pension: a 10-year follow-up of 62 000 people in a Norwegian county population. Int J Epidemiol 2002;**31**(6):1183-91 doi: 10.1093/ije/31.6.1183[published Online First: Epub Date]|.
24. Halford C, Wallman T, Welin L, et al. Effects of self-rated health on sick leave, disability pension, hospital admissions and mortality. A population-based longitudinal study of nearly 15,000 observations among Swedish women and men. BMC Public Health 2012;**12**:1103 doi: 10.1186/1471-2458-12-1103[published Online First: Epub Date]|.
25. Latham K, Peek CW. Self-Rated Health and Morbidity Onset Among Late Midlife US Adults. J Gerontol B-Psychol 2013;**68**(1):107-16 doi: DOI 10.1093/geronb/gbs104[published Online First: Epub Date]|.
26. Benyamini Y, Idler EL. Community studies reporting association between self-rated health and mortality - Additional studies, 1995 to 1998. Res Aging 1999;**21**(3):392-401 doi: Doi 10.1177/0164027599213002[published Online First: Epub Date]|.
27. Nielsen AB, Siersma V, Hiort LC, et al. Self-rated general health among 40-year-old Danes and its association with all-cause mortality at 10-, 20-, and 29 years' follow-up. Scand J Public Health 2008;**36**(1):3-11 doi: 10.1177/1403494807085242[published Online First: Epub Date]|.
28. Schou MB, Krokstad S, Westin S. How is self-rated health associated with mortality? Tidsskr Nor Laegeforen 2006;**126**(20):2644-7
29. Barger SD, Muldoon MF. Hypertension labelling was associated with poorer self-rated health in the Third US National Health and Nutrition Examination Survey. J Hum Hypertens 2006;**20**(2):117-23 doi: 10.1038/sj.jhh.1001950[published Online First: Epub Date]|.
30. Bloom JR, Monterossa S. Hypertension Labeling and Sense of Well-Being. Am J Public Health 1981;**71**(11):1228-32 doi: Doi 10.2105/Ajph.71.11.1228[published Online First: Epub Date]|.
31. Moum T, Naess S, Sorensen T, et al. Hypertension Labeling, Life Events and Psychological Well-Being. Psychol Med 1990;**20**(3):635-46
32. Macdonald LA, Sackett DL, Haynes RB, et al. Labelling in hypertension: A review of the behavioural and psychological consequences. J Chronic Dis 1984;**37**(12):933-42 doi: http://dx.doi.org/10.1016/0021-9681(84)90070-5[published Online First: Epub Date]|.
33. Bianchi GP, Zaccheroni V, Solaroli E, et al. Health-related quality of life in patients with thyroid disorders - A study based on Short-Form 36 and Nottingham Health Profile Questionnaires. Qual Life Res 2004;**13**(1):45-54 doi: Doi 10.1023/B:Qure.0000015315.35184.66[published Online First: Epub Date]|.

For peer review only

**Study population**	**Women (*n*=17,514)**		**Men (*n*=16,220)**

34. Holmen J, Midthjell K, Krüger Ø, et al. The Nord-Trøndelag Health Study 1995-97 (HUNT 2): objectives, methods, and participation. Norsk Epidemiol 2003 **13** (1):19-32
35. Krokstad S, Langhammer A, Hveem K, et al. Cohort Profile: The HUNT Study, Norway. Int J Epidemiol 2012 doi: dys095 [pii] 10.1093/ije/dys095[published Online First: Epub Date]|.
36. Schnittker J, Bacak V. The Increasing Predictive Validity of Self-Rated Health. PLoS One 2014;**9**(1):e84933 doi: 10.1371/journal.pone.0084933[published Online First: Epub Date]|.
37. Idler EL, Benyamini Y. Self-rated health and mortality: A review of twenty-seven community studies. J Health Soc Behav 1997;**38**(1):21-37 doi: Doi 10.2307/2955359[published Online First: Epub Date]|.
38. Manor O, Matthews S, Power C. Dichotomous or categorical response? Analysing self-rated health and lifetime social class. Int J Epidemiol 2000;**29**(1):149-57 doi: Doi 10.1093/Ije/29.1.149[published Online First: Epub Date]|.
39. Bjoro T, Holmen J, Kruger O, et al. Prevalence of thyroid disease, thyroid dysfunction and thyroid peroxidase antibodies in a large, unselected population. The Health Study of Nord-Trondelag (HUNT). Eur J Endocrinol 2000;**143**(5):639-47 doi: 1430639 [pii][published Online First: Epub Date]|.
40. Giron P. Determinants of self-rated health in Spain: differences by age groups for adults. Eur J Public Health 2012;**22**(1):36-40 doi: 10.1093/eurpub/ckq133[published Online First: Epub Date]|.
41. Davies HTO, Crombie IK, Tavakoli M. When can odds ratios mislead? Br Med J 1998;**316**(7136):989-91
42. Langhammer A, Krokstad S, Romundstad P, et al. The HUNT study: participation is associated with survival and depends on socioeconomic status, diseases and symptoms. BMC Med Res Methodol 2012;**12**:143 doi: 10.1186/1471-2288-12-143[published Online First: Epub Date]|.
43. Molarius A, Janson S. Self-rated health, chronic diseases, and symptoms among middle-aged and elderly men and women. J Clin Epidemiol 2002;**55**(4):364-70 doi: http://dx.doi.org/10.1016/S0895-4356(01)00491-7[published Online First: Epub Date]|.
44. Hansen AH, Halvorsen PA, Ringberg U, et al. Socio-economic inequalities in health care utilisation in Norway: a population based cross-sectional survey. BMC Health Serv Res 2012;**12**:336 doi: 10.1186/1472-6963-12-336[published Online First: Epub Date]|.
45. Vingilis E, Wade T, Seeley J. Predictors of adolescent health care utilization. J Adolesc 2007;**30**(5):773-800 doi: http://dx.doi.org/10.1016/j.adolescence.2006.10.001[published Online First: Epub Date]|.
46. Breidablik H-J, Meland E, Lydersen S. Self-rated health during adolescence: stability and predictors of change (Young-HUNT study, Norway). The European Journal of Public Health 2009;**19**(1):73-78 doi: 10.1093/eurpub/ckn111[published Online First: Epub Date]|.
47. Welch HG, Sharp SM, Gottlieb DJ, et al. Geographic variation in diagnosis frequency and risk of death among Medicare beneficiaries. JAMA 2011;**305**(11):1113-8 doi: 10.1001/jama.2011.307[published Online First: Epub Date]|.
48. Fisher ES, Wennberg DE, Stukel TA, et al. The implications of regional variations in Medicare spending. Part 2: health outcomes and satisfaction with care. Ann Intern Med 2003;**138**(4):288-98
49. Wennberg JE. *Tracking medicine. A researcher's quest to understand health care.* Oxford: Oxford University Press, 2010.

For peer review only

	n	%	SRH good (%)		*n*	%	SRH good (%)
Age group							
40-50 years	7058	40.3	77.6		6517	40.2	81.7
51-60 years	5709	32.6	64.4		5328	32.8	72.1
61-70 years	4747	27.1	56.6		4375	27.0	59.6
BMI (kg/m^2)							
<18,5	6821	0.6	58.4		4804	0.2	42.9
18,5-24,9	7026	39.1	72.8		8694	29.7	75.3
25,0-29,9	3489	40.3	68.5		2635	53.8	73.6
>30	104	20.0	56.5		35	16.3	64.8
(0.4% missing)							
Smoking status							
Never smoked daily	6973	40.1	70.3		4849	30.1	80.0
Previous daily smoker	4651	26.7	68.0		6105	37.9	69.8
Daily smoker	5763	33.1	64.3		5172	32.0	69.1
(0.7% missing)							
Alcohol use							
None to low intake	14888	88.7	66.7		11488	73.2	71.0
Moderate intake	1471	8.8	76.8		2839	18.1	78.7
High intake	430	2.6	76.3		1375	8.8	78.3
(3.7% missing)							
Educational level							
< 10 years	8133	48.5	60.5		5711	36.5	63.1
10 – 12 years	5682	33.9	72.8		6615	42.2	76.0
> 12 years	2962	17.7	80.0		3339	21.3	84.7
(3.8% missing)							
Employed							
Yes	11539	67.2	77.1		12309	77.1	79.9
No	5627	32.8	49.0		3665	22.9	49.2
(1.8% missing)							

For peer review only

Any long-term impairment							
No	10348	59.1	85.9		9912	63.0	88.7
Yes	6275	35.8	39.8		5829	37.0	46.0
(4.1% missing)							
Thyroid function							
No thyroid disease	14373	86.2	68.7		7804	94.3	72.1
Unknown hypothyroidism	107	0.6	78.5		16	0.2	68.8
Subclinical hypothyroidism	466	2.8	75.0		150	1.8	66.4
Known hypothyroidism	858	5.1	48.9		124	1.5	58.1
Other thyroid dysfunction	872	5.2	61.4		180	2.2	56.2
(1.3% missing)							
Diabetes mellitus							
No diabetes	11279	64.6	69.4		9196	56.9	74.6
Unknown diabetes	46	0.3	52.2		88	0.5	67.8
Probable diabetes	5728	32.8	65.9		6392	39.6	71.5
Known diabetes	395	2.3	43.1		482	3.0	49.5
(0.4% missing)							
Blood pressure status							
Normotensive	9135	52.6	72.4		6858	42.7	77.5
Unknown mild/moderate hypertension	4473	25.8	67.9		5343	33.3	74.7
Unknown severe hypertension	403	2.4	70.7		349	2.2	73.8
Known hypertension	3327	19.2	54.0		3498	21.8	59.6
(0.9% missing)							
Overall study population, HUNT2	34332	97.3	70.3		30378	98.4	74.9

Table 1. Good self-rated health (SRH) by sex and characteristics of the study population.

Table 2. The association between self-rated health and thyroid function, diabetes mellitus and blood pressure. Odds ratio (OR) of good self-rated health and 95% confidence intervals (95% CI), crude, age adjusted, and adjusted for age, other long-term illness or injury that impairs function in everyday life, smoking habits, alcohol use, educational level, work status and body mass index. Cases with missing data were excluded from the analyses.

	**Women**		**OR (95% CI)**		**Men**		**OR (95% CI)**	
	n	Crude	Age adjusted	Multiple adjusted	*n*	Crude	Age adjusted	Multiple adjusted
**Thyroid function**								
No thyroid dysfunction	12476	1.00	1.00	1.00	7045	1.00	1.00	1.00
Unknown hypothyroidism	92	1.66 (1.05-2.64)	2.00 (1.25-3.19)	1.84 (1.02-3.33)	14	0.85 (0.30-2.45)	0.92 (0.31-2.72)	1.28 (0.35-4.65)
Subclinical hypothyroidism	394	1.37 (1.10-1.69)	1.49 (1.20-1.85)	1.48 (1.13-1.94)	128	0.77 (0.54-1.08)	0.88 (0.62-1.24)	1.13 (0.72-1.76)
Known hypothyroidism	718	0.44 (0.38-0.50)	0.48 (0.41-0.55)	0.49 (0.41-0.59)	107	0.54 (0.37-0.77)	0.63 (0.43-0.91)	0.69 (0.44-1.09)
Other thyroid dysfunction	729	0.72 (0.63-0.83)	0.77 (0.67-0.89)	0.77 (0.65-0.93)	163	0.50 (0.37-0.67)	0.52 (0.38-0.71)	0.58 (0.41-0.84)
**Diabetes mellitus**								
No diabetes	9946	1.00	1.00	1.00	8464	1.00	1.00	1.00
Unknown diabetes	39	0.48 (0.27-0.86)	0.61 (0.34-1.10)	0.66 (0.32-1.37)	82	0.72 (0.46-1.13)	0.88 (0.55-1.39)	1.03 (0.59-1.81)
Probable diabetes	4797	0.85 (0.80-0.91)	0.94 (0.88-1.01)	1.01 (0.92-1.10)	5759	0.86 (0.80-0.92)	0.94 (0.88-1.01)	0.99 (0.91-1.08)
Known diabetes	318	0.33 (0.27-0.41)	0.42 (0.34-0.51)	0.53 (0.41-0.69)	395	0.33 (0.28-0.40)	0.42 (0.35-0.51)	0.55 (0.43-0.70)
**Blood pressure**								
No hypertension	8180	1.00	1.00	1.00	6378	1.00	1.00	1.00
Unknown mild/moderate hypertension	3787	0.81 (0.75-0.87)	1.01 (0.93-1.09)	1.10 (0.99-1.22)	4837	0.86 (0.79-0.93)	1.01 (0.92-1.10)	1.01 (0.92-1.12)
Unknown severe hypertension	325	0.92 (0.74-1.15)	1.34 (1.08-1.68)	1.52 (1.14-2.02)	308	0.85 (0.77-1.08)	1.27 (0.99-1.62)	1.48 (1.09-2.02)
Known hypertension	2745	0.45 (0.41-0.49)	0.59 (0.54-0.65)	0.69 (0.61-0.77)	3118	0.43 (0.39-0.47)	0.54 (0.50-0.60)	0.64 (0.57-0.72)

For peer review only

Disease ignorance and self-rated health, any relationship? The HUNT Study, a cross-sectional survey

Pål Jørgensen, Arnulf Langhammer, Steinar Krokstad, Siri Forsmo

Department of Public Health and General Practice, Norwegian University of Science and Technology, 7489 Trondheim, Norway Pål Jørgensen
PhD Candidate
HUNT Research Centre, Department of Public Health and General Practice, Norwegian University of Science and Technology, 7600 Levanger, Norway Arnulf Langhammer
Professor, Head of HUNT Databank
HUNT Research Centre, Department of Public Health and General Practice, Norwegian University of Science and Technology, Steinar Krokstad
Professor, Head of HUNT Research Center
Department of Public Health and General Practice, Norwegian University of Science and Technology, Siri Forsmo
Professor, Head of Department

Correspondence to: P Jørgensen pal_jorgensen@ntnu.no

Field Code Changed

Keywords: Self-rated health, awareness, hypothyroidism, diabetes, hypertension.

Word count: ~~2995~~ 3359

For peer review only

ABSTRACT

Objective To explore if awareness versus unawareness of thyroid dysfunction, diabetes mellitus or hypertension is associated with self-rated health.

Design Large-scale, cross sectional population based study. The association between thyroid function, diabetes mellitus, and blood pressure and self-rated health was explored by multiple logistic regression analysis.

Setting The second survey of the Nord-Trøndelag Health Study, HUNT2, 1995-97.

Participants 33,734 persons aged 40-70 years.

Primary outcome measures Logistic regression was used to estimate odds ratios for good self-rated health as a function of thyroid status, diabetes mellitus status and blood pressure status.

Results Persons aware of their hypothyroidism, diabetes mellitus, or hypertension reported poorer self-rated health than individuals without such conditions. Women with unknown and subclinical hypothyroidism reported better self-rated health than women with normal thyroid status. Both in women and men, unknown and probable diabetes, as well as unknown mild/moderate hypertension was not associated with poorer health. Further, persons with unknown severe hypertension reported better health than normotensive persons.

Conclusions People with undiagnosed, but prevalent hypothyroidism, diabetes mellitus, and hypertension often have good self-rated health, whilst when aware of their diagnoses, they report reduced self-rated health. Use of screening, more sensitive tests, and widened diagnostic criteria might have a negative effect on perceived health in the population.

For peer review only

STRENGTHS AND LIMITATIONS OF THIS STUDY

Strengths

- Sample from a large-scale, general population
- High-prevalent diseases under study; ensuring statistical power in subgroup analyses

Limitations

- Study mainly based on self-reported data
- Cross-sectional study; susceptibility to confounding and impossibility to assume causal relationships

INTRODUCTION

Guidelines for prevention and treatment have been developed for most high prevalent diseases in western countries aiming for reduction of morbidity and mortality by interventions mainly in primary health care (PHC).

In the society there seems to be an increasing conviction of achievable zero-vision regarding risks and diseases. Part of the strategy is to detect risk factors and pre-diseases in even earlier stages. From a secondary or tertiary health care level, this might seem reasonable since intervention on many individuals with specific risk factors presumably can prevent or delay disease or progression of disease. Further, health authorities and hospital clinicians regularly raise concern of the lack of detection of risk factors, of subclinical conditions and of achieving treatment goals.[1-7] Norwegian studies have shown that guidelines are often difficult to implement and adhere to in PHC.[8 9] According to guidelines, most individuals would be defined as at risk and resources needed to handle this appropriately could destabilize the entire health care system.[10-12] An American review pointed out knowledge, attitude,

For peer review only

and behaviour as barriers of physician's adherence to clinical guidelines.[13] In an already complex and busy PHC-setting, one might expect that resources used for disease prevention and case finding have to compete with resources for handling acknowledged disease. Also, physicians might want to avoid increasing disease related burden for patients, in line with the old wisdom; "primum non nocere".[14] Thus the risk and disease zero-visions in society and among politicians are seldom shared by PHC professionals.

When guidelines, mainly based on research from high risk hospital populations, are applied on low risk populations in PHC, more healthy individuals are identified as being at risk or are given diagnoses. Also the widened inclusion criteria for diagnoses in general and use of more sensitive tests contribute to define more individuals at risk or as unhealthy.[15] Possible undesirable outcomes of such strategies remain unclear.

Self-rated health (SRH) is a valid and widely used measure of general health in epidemiologic research.[16-19] It is associated with several clinical conditions often seen in PHC, with recovery [20-23] and is found to predict morbidity, sick leave, and disability pension,[24 25] as well as mortality.[26-28] The majority of studies describing association between labelling of disease per se and SRH have focused on arterial hypertension.[29-32] However, one study has indicated reduced SRH among individuals labelled with thyroid dysfunctions.[33]

The aim of this study was to investigate whether persons' awareness versus unawareness of thyroid dysfunction, diabetes mellitus, or hypertension was associated with their SRH, as reported in a population-based health study in Norway.

For peer review only

METHODS

Study population

The data sample in this study stem from the second wave of the Nord-Trøndelag Health Study (HUNT2) conducted in 1995-97 in the county of Nord-Trøndelag, Norway. All individuals aged 20 years and older, living in the county, were invited (94,194 individuals). In all, 66.7% of men (n=30,860) and 75.5% of women (n=35,280) participated. The survey consisted of both questionnaires and measurements, and is described previously in detail.[34 35] In our study we included answers from the main questionnaire and the baseline measurements for persons aged 40-70 years. The age span was chosen because thyroid stimulating hormone (TSH) was analysed in all women and in 50% of men at this age, of a rather low disease burden in people younger than 40 years, and of a lower attendance rate under and above this age span. A total of 24,950 individuals had TSH measurements and answered thyroid questions, thus were eligible for analysis on thyroid dysfunction, whilst in the analysis of diabetes mellitus and blood pressure, 33,734 individuals were included in all.

Self-rated health

The first question in the main questionnaire in HUNT2, answered before attending the examination stations, was "How is your health at the moment?", with answer alternatives; "very good", "good", "not so good", and "poor". This short version of SRH measure is shown to be a valid predictor of mortality. [18 28 36 37] We dichotomized the answers into "good" (very good, good) and "poor" (not so good, poor). Dichotomization of multinomial SRH is commonly performed, and has been validated by Manor et. al.[38]

Thyroid function

For peer review only

The participants answered questions on history of hypo- and hyperthyroidism, goitre, other thyroid diseases, and treatment with thyroxin, radio-iodine, surgery, or thyreostatic medication.

Serum TSH and free T4 were analysed at the Hormone laboratory, Aker University Hospital, Norway. The laboratory reference value for TSH, as defined prior to the survey, was 0.2-4.5 mU/L and for free T_4 8.0-20.0 pmol/L. If TSH was <0.2 mU/L or >4.0 mU/L, and/or if the participant reported any thyroid disease, serum free T_4 was also measured.[39]

Individuals reporting no previous thyroid disease and having TSH within reference range were categorized as “no thyroid disease” and chosen as reference category. No previous thyroid disease combined with TSH >4.5 mU/L and free T_4 <8.0 pmol/L was defined as unknown hypothyroidism. No previous thyroid disease combined with TSH >4.5 mU/L and free T_4 8.0 – 20.0 pmol/L was defined as subclinical hypothyroidism. Individuals reporting hypothyroidism and use of thyroxin were classified as having known hypothyroidism, regardless of the TSH and T4 levels. Affirmative answers to other thyroid related questions or measures outside reference range in the remainders were classified as other thyroid dysfunction.

Diabetes mellitus

Diabetes mellitus was assessed through self-report and blood samples. Serum glucose was analysed at Levanger Hospital, Norway. Those reporting no diabetes and having normal glucose levels (<5.5 mmol/L) were classified as “no diabetes” and were chosen as reference category. No self-reported diabetes and non-fasting glucose >11.0 mmol/L was categorized as unknown diabetes, whereas no diabetes and non-fasting glucose 5.5-11.0 mmol/L was

For peer review only

categorized as probable diabetes. Self-reported diabetes was classified as known diabetes regardless of the glucose level.

Blood pressure

In the questionnaire participants were asked about the doctor's advice after the latest blood pressure measurement prior to participation in HUNT. The answer categories were: "no follow-up and no medication necessary", "recommended follow-up examination but not to take medicine", "start or continue taking medicine for high blood pressure", or "never measured". At HUNT2, mean systolic and mean diastolic arterial blood pressure (BP) of measurement 2 and 3 was categorized into normal (systolic (s) BP < 140 mmHg and diastolic (d) BP < 90 mmHg), mild hypertension (sBP 140-159 mmHg and dBP <100 mmHg or sBP <160 mmHg and dBP 90-99 mmHg), moderate hypertension (sBP 160-179 mmHg and dBP <109 mmHg or sBP <180 mmHg and dBP 100-109 mmHg), and severe hypertension (sBP>180 mmHg or dBP>110 mmHg). We constructed a new variable to define normotensive (reference), unknown mild- and moderate hypertensive, unknown severe hypertensive, and known hypertensive persons on the basis of self-report and measures.

Statistical analysis

The descriptive analyses of the study population were stratified by gender, and we used chi-square tests to examine any difference in proportions of SRH between the independent variables. Gender-stratified multiple logistic regression were used to estimate odds ratio (OR) with 95% confidence intervals (CI) for good SRH, as a function of thyroid status, diabetes mellitus status and blood pressure status, in separate unadjusted, age adjusted, and multi adjusted analyses for each condition.

For peer review only

Age, smoking, alcohol consumption, body mass index (BMI), working- and educational status, and self-reported limiting long-term illness or injury are associated with SRH[40] and the diseases under study, but likely not affected by SRH or the diseases. Hence, these variables were included, a priori, as confounders in the models. Age was categorized into age groups; 40-49 years, 50-59 years, and 60-70 years. Smoking status was categorized into never smoked daily, previous daily smoker, and current daily smoker. Alcohol units (AU) were defined as number of glasses of wine, beer or liquor. Those reporting to be teetotalers or to have alcohol intake less than four times a month or less than seven AU per two weeks were categorized as low consumers, those reporting drinking five to eight times a month or 8-14 AU per two weeks as moderate consumers, and those drinking more often than eight times a month or more than 14 AU as heavy consumers. BMI ($kg/m^{2)}$ was calculated of measured height and weight and categorized according to The World Health Organization's (WHO) definition; underweight (18.5 kg/m^2), normal weight (18.5-24.9 kg/m^2), overweight (25.0-29.9 kg/m^2) and obese (>30.0 kg/m^2). People reporting paid- or self-employed work were classified as working, otherwise as not working. Educational level was categorized into <10 years, 10-12 years and >12 years. We chose affirmative answer to the question "Do you suffer from any long-term illness or injury (at least one year) of a physical or psychological nature that impairs your functioning in your everyday life?" to represent all relevant chronic medical conditions that could confound the results.

To examine whether the association of the three disease statuses with SRH differed by categories of the other independent variables, we used likelihood ratio-tests with p-value for statistical interaction. We tested for multicollinearity between the independent variables, by linear regression.

For peer review only

In an additional analysis, the association between SRH and having had one or more medical consultations during the last year was investigated by logistic regression models, stratified by gender, both in the total study population and after exclusion of persons with diagnoses under study.

All analyses were preformed with IBM SPSS Statistics version 20 for windows.

The study was approved by the Regional Committee for Medical Research. All participants signed written informed consent.

RESULTS

In all age categories, a higher proportion of men than women reported good SRH ($p<0.001$), and in both sexes the proportion reporting good SRH declined by age ($p<0.002$) (Table 1). The proportion reporting good SRH was lower in overweight, obese, and underweight women, than in normal weight women ($p<0.001$). In men the proportion reporting good SRH declined between the normal weight group to overweight-, obese-, and underweight group ($p<0.001$). In previous and current female smokers, a lower proportion reported good SRH compared to non smokers ($p<0.001$). In men, the proportion reporting good SRH was higher among non smokers than among previous and current smokers ($p<0.001$), whereas there was no difference between previous and current smokers. The proportion reporting good SRH was higher in moderate and heavy alcohol consumers than in low consumers, increased with education and was higher for participants in paid work ($p<0.001$). Underweight men and persons with "any long-term impairment" had the lowest proportion reporting good SRH of all groups. The proportion reporting good SRH in the overall HUNT 2 study population differed from the proportions reported in persons without thyroid disease and normotensive persons ($p<0.01$), but not from persons without diabetes mellitus. However, the absolute differences were small.

For peer review only

There was no multicollinearity between the independent variables.

Thyroid function

Thyroid dysfunctions, both known and unknown were more often observed among women than among men (p<0.001) (Table 1). Women with known hypothyroidism had lower odds of reporting good SRH (OR 0.49 (95% CI 0.41 to 0.59) compared to women without thyroid disease in the adjusted analyses, but women with unknown or subclinical hypothyroidism had 84% and 48% higher odds, respectively, of reporting good SRH compared to the odds of women without thyroid disease (Table 2). The association between thyroid function and SRH was basically unchanged after inclusion of confounder variables. ~~A c~~Corresponding, but non-significant associations ~~pattern~~ were~~as~~ found among men.

Diabetes mellitus

The prevalence of unknown, probable, and known diabetes was slightly higher in men than in women (p<0.001) (Table 1). Women with known diabetes mellitus had lower odds of good SRH than those without diabetes in the adjusted analyses; OR 0.53 (95% CI 0.41 to 0.69), whereas in women with unknown or possible diabetes mellitus, the odds of good SRH were similar to the odds among persons without diabetes, in the adjusted analyses (Table 2). In the adjusted analyses, the association between unknown and probable diabetes mellitus with poor SRH, found in the crude analyses, disappeared when age was included in the model in women and men, but also by inclusion of working status alone in women. In men, the association of diabetes status with SRH differed by levels of education. Among men without higher education (12 years or less), the odds of good SRH were as in the main-effect model (Table 2). However in men with higher education, the ORs of good SRH were barely significantly lower among men with unknown diabetes (OR 0.29 (95% CI 0.09-1.00)) and among men with

For peer review only

possible diabetes (OR 0.79 (95% CI 0.63-1.00)) compared to men without diabetes. Among men with known diabetes in this stratum, the OR of good SRH was 0.31 (95% CI 0.17-0.54) compared to men without diabetes.

Blood pressure

The prevalence of unknown mild and moderate hypertension was higher in men than in women (p<0.001). Unknown severe hypertension and known hypertension were equally distributed between women and men (Table 1). Women with known hypertension had lower odds of reporting good SRH than normotensive women, in the adjusted analyses. The figures were similar in men (Table 2). In contrast; compared to normotensive women, those with unknown severe hypertension had 52 % higher odds of reporting good SRH, with similar figures in men. Persons with unknown mild and moderate hypertension reported good SRH similar to the normotensive ones. Adjusted for age, the association between unknown mild and moderate hypertension and poor SRH disappeared, simultaneously; unknown severe hypertension became associated with good SRH in women. In men, age had to be added along with either education- or working status to achieve the latter association.

Additional analyses

Women with poor SRH had more than six times the odds of those with good SRH to have had a medical consultation during the last year; OR 6.29 (95% CI 5.47 to 7.22). For men the corresponding OR was 5.53 (95% CI 4.86 to 6.29). After exclusion of persons with diagnosed thyroid disease, diabetes mellitus, known hypertension, and "any long-term disease", the corresponding OR was 3.71 (95% CI 2.90 to 4.73), with similar figures in men.

DISCUSSION

For peer review only

This large population-based study showed that persons with known thyroid dysfunction, diabetes mellitus, and hypertension were less likely to report good SRH than those without such conditions. Less expected, persons with unknown and subclinical hypothyroidism were more likely to report good SRH than those without thyroid disease. Similarly, those with unknown severe hypertension were more likely to report good SRH, compared to persons with normal blood pressure. In general, persons with unknown diabetes and unknown mild/moderate hypertension, reported good health, just like the reference group.

In general, of the confounders, age seemed to influence the association between disease statuses and SRH most when adjusted for. Age was found to explain the association of poor SRH with unknown and probable diabetes, and with unknown mild and moderate hypertension. In women, age even contributed to an association between unknown severe hypertension and good SRH. There seemed to be a linear decrease by age categories in the association with good SRH. The way age is known to be related to both disease and SRH makes these findings reasonable.

Although both a qualitative and quantitative measure of association, the estimated ORs in our study will, if interpreted as relative risks, overestimate the case, because the prevalence of good SRH is high in all groups.[41]

The main strengths of this study were the numbers of participants, that the diseases we studied were high-prevalent, and that we could assume representativeness to the general population regarding the variables included. It is known that individuals with high burden of symptoms are less likely to attend surveys, but so far there has been little evidence that non-participation on this basis introduce substantial bias in associational studies.[42] We studied diseases with generally low symptom-burden, and expect selection bias to be negligible.

For peer review only

The main limitation was possibly that we relied on self-reported data on both dependent and independent variables. The validity of self reported measures relevant for this study is questionable. However, if any, there should be a non-differential misclassification, that only should underestimate the associations found.

There is always a possibility of residual confounding in non-randomized study designs, and due to the observational, cross-sectional design we neither can assume a causal relationship.

Bias due to differential detection of disease in the study population could lead to a type 1 error. Hypothyroidism and diabetes mellitus are often associated with vague symptoms such as tiredness and weakness. These symptoms are strongly associated with reduced SRH.[43] It is likely that presenting such symptoms for the GP would result in measurements of TSH, free T4, and serum glucose, thus reveal any related dysfunction. Low self-perceived health increases the probability to visit a GP.[44] The social security covers most costs related to clinical measurements and blood sampling in Norway, thus we expect GPs to have a low threshold to measure thyroid function, blood pressure or glucose levels. People who do not consult their GP will not have diseases with mild or no symptoms diagnosed, and their personality could be characterized by less worrying and more optimistic attitude to health being reflected in better SRH.[45]

On the other hand, the association between known disease and poor SRH could in fact be explained physiologically due to the pathological effect of disease. Lack of corresponding association between ignored, but prevalent disease and poor SRH is not in line with this hypothesis, rising question of a possible adverse effect of disease labelling. However, confounding by severity of disease cannot be ruled out.

For peer review only

Due to low numbers of persons having unknown hypothyroidism and diabetes mellitus, the results should be interpreted with caution. On the other hand, there were high numbers in the subclinical and probable groups, and analyses of these groups did not show any associations with poor SRH, also rising question of a possible disease labelling effect.

The fact that the HUNT2 survey was carried out nearly 20 years ago raises a question of generalisability to today's population. Stability of SRH over time has not been investigated in our study population, although investigated among adolescents.[46] We do not expect the association between low burden- or subclinical disease with SRH to be time dependent to the extent that it would change our results considerably. Neither do we expect the changes in prevalence of most explanatory variables to influence the associations found.

Consistent findings regarding unknown, subclinical, and probable disease, versus known disease, could indicate a potential adverse effect of disease labelling. Although early detection of disease is protective on morbidity and mortality for many diseases, low SRH is also shown to be associated morbidity and mortality.[25-27] This is an important aspect in the debate of presymptomatic case-finding.

The BMJ's Too Much Medicine campaign aims to highlight the threat to human health posed by overdiagnosis and the waste of resources on unnecessary care (http://www.bmj.com/too-much-medicine). American data have shown that the majority of all health care includes preference-sensitive and supply-sensitive services. The extent of these services varies greatly without necessarily leading to better health.[47 48] A great deal of activity in such services is based on identifying subclinical disease. The negative health effect this might have, modulated through reduced SRH, can explain why population mortality is not reduced in areas with high frequency of diagnoses.

For peer review only

Both public and academic debates are often characterised by the conviction that all medical treatment is efficient. Wennberg showed that a relatively small proportion of all medical treatment is indisputably good for health.[49] The concern for unrevealed risk factors and subclinical conditions might lead to unnecessary costly health care interventions, increase supply-sensitive services without positive health effect and have a negative influence on people's general and self-perceived health causing more harm than good.[15]

Of ethical reasons, the possible causal effect of disease labelling on SRH is impossible to assess in a randomized controlled trial. Our study emphasizes the need for more prospective research to investigate potential health effects of disease labelling and early diagnosis.

CONCLUSION

Our data suggest that early identification of disease may imply a negative effect on SRH, and to the extent that SRH has been associated with greater mortality, this may lead to harm. However, as it is also known that diseases such as diabetes and hypertension also lead to increased mortality when detected after cardiovascular and metabolic complications have developed, it remains to be seen if an early identification or a late detection strategy would provide optimal health for the population. ~~early identification of disease may imply a negative effect on SRH and thereby eventually cause more harm than good. On a population level, such efforts might increase the costs in health care and the proportion of supply-sensitive services without any documented positive health effect. More research is needed concerning the possible adverse effects of disease labelling on SRH and mortality.~~

FOOTNOTES

Copyright The Corresponding Author has the right to grant on behalf of all authors and does grant on behalf of all authors, a worldwide licence to the Publishers and its licensees in perpetuity, in all forms, formats and media (whether known now or created in the future), to i)

Field Code Changed

publish, reproduce, distribute, display and store the Contribution, ii) translate the Contribution into other languages, create adaptations, reprints, include within collections and create summaries, extracts and/or, abstracts of the Contribution, iii) create any other derivative work(s) based on the Contribution, iv) to exploit all subsidiary rights in the Contribution, v) the inclusion of electronic links from the Contribution to third party material where-ever it may be located; and, vi) licence any third party to do any or all of the above.

Competing interests All authors have completed the ICMJE uniform disclosure form at www.icmje.org/coi_disclosure.pdf and declare: no support from any organisation for the submitted work; no financial relationships with any organisations that might have an interest in the submitted work in the previous three years; no other relationships or activities that could appear to have influenced the submitted work.

Field Code Changed

Contributors All authors meet the four criteria for authorship recommended by the International Committee of Medical Journal Editors. SF, AL, and SK have been active supervisors in study conception, design, conduct, interpretation, and reporting. PJ analyzed the data and drafted the manuscript. Critical revisions were done by all supervisors and all authors approved the final version of the manuscript. SF, AL, SK and PJ are joint guarantors.

Acknowledgements The Nord-Trøndelag Health Study (The HUNT Study) is a collaboration between HUNT Research Centre (Faculty of Medicine, Norwegian University of Science and Technology NTNU), Nord-Trøndelag County Council, Central Norway Health Authority, and the Norwegian Institute of Public Health. The authors would like to thank Bjørn Olav Åsvold for his contribution in planning of the thyroid-part of the study.

Transparency declaration PJ affirms that this manuscript is an honest, accurate, and transparent account of the study being reported; that no important aspects of the study have been omitted; and that there were no discrepancies from the study as planned.

Ethical approval The study was approved by the Regional Committee for Medical Research. All participants signed written informed consent.

Funding No external funding. PJ received a PhD-grant, funded by the Norwegian University of Science and Technology, NTNU.

Data sharing statement No additional data available.

For peer review only

1. Ziemer DC, Miller CD, Rhee MK, et al. Clinical inertia contributes to poor diabetes control in a primary care setting. Diabetes Educ 2005;**31**(4):564-71 doi: 10.1177/0145721705279050[published Online First: Epub Date]|.
2. Borzecki AM, Wong AT, Hickey EC, et al. Hypertension control: how well are we doing? Arch Intern Med 2003;**163**(22):2705-11 doi: 10.1001/archinte.163.22.2705[published Online First: Epub Date]|.
3. Andros V, Egger A, Dua U. Blood pressure goal attainment according to JNC 7 guidelines and utilization of antihypertensive drug therapy in MCO patients with type 1 or type 2 diabetes. J Manag Care Pharm 2006;**12**(4):303-9
4. Liddy C, Singh J, Hogg W, et al. Quality of cardiovascular disease care in Ontario, Canada: missed opportunities for prevention - a cross sectional study. BMC Cardiovasc Disord 2012;**12**:74 doi: 10.1186/1471-2261-12-74[published Online First: Epub Date]|.
5. Huebschmann AG, Mizrahi T, Soenksen A, et al. Reducing clinical inertia in hypertension treatment: a pragmatic randomized controlled trial. J Clin Hypertens (Greenwich) 2012;**14**(5):322-9 doi: 10.1111/j.1751-7176.2012.00607.x[published Online First: Epub Date]|.
6. Alkerwi Aa, Pagny S, Lair M-L, et al. Level of Unawareness and Management of Diabetes, Hypertension, and Dyslipidemia among Adults in Luxembourg: Findings from ORISCAV-LUX Study. PLoS One 2013;**8**(3):e57920 doi: 10.1371/journal.pone.0057920[published Online First: Epub Date]|.
7. Khan N, Chockalingam A, Campbell NR. Lack of control of high blood pressure and treatment recommendations in Canada. Can J Cardiol 2002;**18**(6):657-61
8. Hetlevik I, Holmen J, Kruger O, et al. Fifteen years with clinical guidelines in the treatment of hypertension - still discrepancies between intentions and practice. Scand J Prim Health Care 1997;**15**(3):134-40 doi: Doi 10.3109/02813439709018503[published Online First: Epub Date]|.
9. Hetlevik I, Holmen J, Midthjell K. Treatment of diabetes mellitus - physicians' adherence to clinical guidelines in Norway. Scand J Prim Health Care 1997;**15**(4):193-97 doi: Doi 10.3109/02813439709035027[published Online First: Epub Date]|.
10. Petursson H, Getz L, Sigurdsson JA, et al. Current European guidelines for management of arterial hypertension: Are they adequate for use in primary care? Modelling study based on the Norwegian HUNT 2 population. BMC Fam Pract 2009;**10** doi: Artn 70 Doi 10.1186/1471-2296-10-70[published Online First: Epub Date]|.
11. Petursson H, Getz L, Sigurdsson JA, et al. Can individuals with a significant risk for cardiovascular disease be adequately identified by combination of several risk factors? Modelling study based on the Norwegian HUNT 2 population. J Eval Clin Pract 2009;**15**(1):103-09 doi: DOI 10.1111/j.1365-2753.2008.00962.x[published Online First: Epub Date]|.
12. Getz L, Sigurdsson JA, Hetlevik I, et al. Estimating the high risk group for cardiovascular disease in the Norwegian HUNT 2 population according to the 2003 European guidelines: modelling study. Br Med J 2005;**331**(7516):551-54A doi: DOI 10.1136/bmj.38555.648623.8F[published Online First: Epub Date]|.
13. Cabana MD, Rand CS, Powe NR, et al. Why don't physicians follow clinical practice guidelines?: A framework for improvement. JAMA 1999;**282**(15):1458-65 doi: 10.1001/jama.282.15.1458[published Online First: Epub Date]|.
14. Smith CM. Origin and uses of primum non nocere--above all, do no harm! J Clin Pharmacol 2005;**45**(4):371-7 doi: 10.1177/0091270004273680[published Online First: Epub Date]|.
15. Moynihan R, Doust J, Henry D. Preventing overdiagnosis: how to stop harming the healthy. Br Med J 2012;**344** doi: Artn E3502 Doi 10.1136/Bmj.E3502[published Online First: Epub Date]|.
16. Miilunpalo S, Vuori I, Oja P, et al. Self-rated health status as a health measure: the predictive value of self-reported health status on the use of physician services and on mortality in the

Formatted: English (U.S.)

Formatted: English (U.S.)

Formatted: English (U.S.)

Formatted: English (U.S.)

Formatted: English (U.S.)

Formatted: English (U.S.)

For peer review only

working-age population. J Clin Epidemiol 1997;**50**(5):517-28 doi: S0895-4356(97)00045-0 [pii][published Online First: Epub Date]|.
17. Meurer LN, Layde PM, Guse CE. Self-rated health status: a new vital sign for primary care? WMJ 2001;**100**(7):35-9
18. Bowling A. Just one question: If one question works, why ask several? J Epidemiol Community Health 2005;**59**(5):342-5 doi: 59/5/342 [pii] 10.1136/jech.2004.021204[published Online First: Epub Date]|.
19. Lundberg O, Manderbacka K. Assessing reliability of a measure of self-rated health. Scand J Soc Med 1996;**24**(3):218-24
20. Bopp M, Braun J, Gutzwiller F, et al. Health risk or resource? Gradual and independent association between self-rated health and mortality persists over 30 years. PLoS One 2012;**7**(2):e30795 doi: 10.1371/journal.pone.0030795 PONE-D-11-20499[pii][published Online First: Epub Date]|.
21. Moller L, Kristensen TS, Hollnagel H. Self rated health as a predictor of coronary heart disease in Copenhagen, Denmark. J Epidemiol Community Health 1996;**50**(4):423-8
22. Kaplan GA, Goldberg DE, Everson SA, et al. Perceived health status and morbidity and mortality: Evidence from the Kuopio Ischaemic Heart Disease Risk Factor Study. Int J Epidemiol 1996;**25**(2):259-65 doi: Doi 10.1093/Ije/25.2.259[published Online First: Epub Date]|.
23. Krokstad S, Johnsen R, Westin S. Social determinants of disability pension: a 10-year follow-up of 62 000 people in a Norwegian county population. Int J Epidemiol 2002;**31**(6):1183-91 doi: 10.1093/ije/31.6.1183[published Online First: Epub Date]|.
24. Halford C, Wallman T, Welin L, et al. Effects of self-rated health on sick leave, disability pension, hospital admissions and mortality. A population-based longitudinal study of nearly 15,000 observations among Swedish women and men. BMC Public Health 2012;**12**:1103 doi: 10.1186/1471-2458-12-1103[published Online First: Epub Date]|.
25. Latham K, Peek CW. Self-Rated Health and Morbidity Onset Among Late Midlife US Adults. J Gerontol B-Psychol 2013;**68**(1):107-16 doi: DOI 10.1093/geronb/gbs104[published Online First: Epub Date]|.
26. Benyamini Y, Idler EL. Community studies reporting association between self-rated health and mortality - Additional studies, 1995 to 1998. Res Aging 1999;**21**(3):392-401 doi: Doi 10.1177/0164027599213002[published Online First: Epub Date]|.
27. Nielsen AB, Siersma V, Hiort LC, et al. Self-rated general health among 40-year-old Danes and its association with all-cause mortality at 10-, 20-, and 29 years' follow-up. Scand J Public Health 2008;**36**(1):3-11 doi: 10.1177/1403494807085242[published Online First: Epub Date]|.
28. Schou MB, Krokstad S, Westin S. How is self-rated health associated with mortality? Tidsskr Nor Laegeforen 2006;**126**(20):2644-7
29. Barger SD, Muldoon MF. Hypertension labelling was associated with poorer self-rated health in the Third US National Health and Nutrition Examination Survey. J Hum Hypertens 2006;**20**(2):117-23 doi: 10.1038/sj.jhh.1001950[published Online First: Epub Date]|.
30. Bloom JR, Monterossa S. Hypertension Labeling and Sense of Well-Being. Am J Public Health 1981;**71**(11):1228-32 doi: Doi 10.2105/Ajph.71.11.1228[published Online First: Epub Date]|.
31. Moum T, Naess S, Sorensen T, et al. Hypertension Labeling, Life Events and Psychological Well-Being. Psychol Med 1990;**20**(3):635-46
32. Macdonald LA, Sackett DL, Haynes RB, et al. Labelling in hypertension: A review of the behavioural and psychological consequences. J Chronic Dis 1984;**37**(12):933-42 doi: http://dx.doi.org/10.1016/0021-9681(84)90070-5[published Online First: Epub Date]|.
33. Bianchi GP, Zaccheroni V, Solaroli E, et al. Health-related quality of life in patients with thyroid disorders - A study based on Short-Form 36 and Nottingham Health Profile Questionnaires. Qual Life Res 2004;**13**(1):45-54 doi: Doi 10.1023/B:Qure.0000015315.35184.66[published Online First: Epub Date]|.

Formatted: English (U.S.)

Formatted: English (U.S.)

Formatted: English (U.S.)

Field Code Changed

Formatted: English (U.S.)

Formatted: English (U.S.)

For peer review only

34. Holmen J, Midthjell K, Krüger Ø, et al. The Nord-Trøndelag Health Study 1995-97 (HUNT 2): objectives, methods, and participation. Norsk Epidemiol 2003 **13** (1):19-32
35. Krokstad S, Langhammer A, Hveem K, et al. Cohort Profile: The HUNT Study, Norway. Int J Epidemiol 2012 doi: dys095 [pii] 10.1093/ije/dys095[published Online First: Epub Date]|.
36. Schnittker J, Bacak V. The Increasing Predictive Validity of Self-Rated Health. PLoS One 2014;**9**(1):e84933 doi: 10.1371/journal.pone.0084933[published Online First: Epub Date]|.
37. Idler EL, Benyamini Y. Self-rated health and mortality: A review of twenty-seven community studies. J Health Soc Behav 1997;**38**(1):21-37 doi: Doi 10.2307/2955359[published Online First: Epub Date]|.
38. Manor O, Matthews S, Power C. Dichotomous or categorical response? Analysing self-rated health and lifetime social class. Int J Epidemiol 2000;**29**(1):149-57 doi: Doi 10.1093/Ije/29.1.149[published Online First: Epub Date]|.
39. Bjoro T, Holmen J, Kruger O, et al. Prevalence of thyroid disease, thyroid dysfunction and thyroid peroxidase antibodies in a large, unselected population. The Health Study of Nord-Trondelag (HUNT). Eur J Endocrinol 2000;**143**(5):639-47 doi: 1430639 [pii][published Online First: Epub Date]|.
40. Giron P. Determinants of self-rated health in Spain: differences by age groups for adults. Eur J Public Health 2012;**22**(1):36-40 doi: 10.1093/eurpub/ckq133[published Online First: Epub Date]|.
41. Davies HTO, Crombie IK, Tavakoli M. When can odds ratios mislead? Br Med J 1998;**316**(7136):989-91
42. Langhammer A, Krokstad S, Romundstad P, et al. The HUNT study: participation is associated with survival and depends on socioeconomic status, diseases and symptoms. BMC Med Res Methodol 2012;**12**:143 doi: 10.1186/1471-2288-12-143[published Online First: Epub Date]|.
43. Molarius A, Janson S. Self-rated health, chronic diseases, and symptoms among middle-aged and elderly men and women. J Clin Epidemiol 2002;**55**(4):364-70 doi: http://dx.doi.org/10.1016/S0895-4356(01)00491-7[published Online First: Epub Date]|.
44. Hansen AH, Halvorsen PA, Ringberg U, et al. Socio-economic inequalities in health care utilisation in Norway: a population based cross-sectional survey. BMC Health Serv Res 2012;**12**:336 doi: 10.1186/1472-6963-12-336[published Online First: Epub Date]|.
45. Vingilis E, Wade T, Seeley J. Predictors of adolescent health care utilization. J Adolesc 2007;**30**(5):773-800 doi: http://dx.doi.org/10.1016/j.adolescence.2006.10.001[published Online First: Epub Date]|.
46. Breidablik H-J, Meland E, Lydersen S. Self-rated health during adolescence: stability and predictors of change (Young-HUNT study, Norway). The European Journal of Public Health 2009;**19**(1):73-78 doi: 10.1093/eurpub/ckn111[published Online First: Epub Date]|.
47. Welch HG, Sharp SM, Gottlieb DJ, et al. Geographic variation in diagnosis frequency and risk of death among Medicare beneficiaries. JAMA 2011;**305**(11):1113-8 doi: 10.1001/jama.2011.307[published Online First: Epub Date]|.
48. Fisher ES, Wennberg DE, Stukel TA, et al. The implications of regional variations in Medicare spending. Part 2: health outcomes and satisfaction with care. Ann Intern Med 2003;**138**(4):288-98
49. Wennberg JE. *Tracking medicine. A researcher's quest to understand health care.* Oxford: Oxford University Press, 2010.

Formatted: English (U.S.)
Formatted: English (U.S.)
Formatted: English (U.S.)
Formatted: English (U.S.)
Field Code Changed
Formatted: English (U.S.)
Formatted: English (U.S.)
Field Code Changed
Formatted: English (U.S.)
Formatted: English (U.S.)

For peer review only

STROBE 2007 (v4) Statement—Checklist of items that should be included in reports of *cross-sectional studies*

Section/Topic	Item #	Recommendation	Reported on page #
**Title and abstract**	1	(*a*) Indicate the study's design with a commonly used term in the title or the abstract	2
		(*b*) Provide in the abstract an informative and balanced summary of what was done and what was found	2
**Introduction**			
Background/rationale	2	Explain the scientific background and rationale for the investigation being reported	3-4
Objectives	3	State specific objectives, including any prespecified hypotheses	4
**Methods**			
Study design	4	Present key elements of study design early in the paper	2-3
Setting	5	Describe the setting, locations, and relevant dates, including periods of recruitment, exposure, follow-up, and data collection	5
Participants	6	(*a*) Give the eligibility criteria, and the sources and methods of selection of participants	5
Variables	7	Clearly define all outcomes, exposures, predictors, potential confounders, and effect modifiers. Give diagnostic criteria, if applicable	5-8
Data sources/ measurement	8*	For each variable of interest, give sources of data and details of methods of assessment (measurement). Describe comparability of assessment methods if there is more than one group	5-8
Bias	9	Describe any efforts to address potential sources of bias	7-8
Study size	10	Explain how the study size was arrived at	5
Quantitative variables	11	Explain how quantitative variables were handled in the analyses. If applicable, describe which groupings were chosen and why	5-8
Statistical methods	12	(*a*) Describe all statistical methods, including those used to control for confounding	7-8
		(*b*) Describe any methods used to examine subgroups and interactions	8
		(*c*) Explain how missing data were addressed	Table 2
		(*d*) If applicable, describe analytical methods taking account of sampling strategy	-
		(*e*) Describe any sensitivity analyses	-
**Results**			

For peer review only

Participants	13*	(a) Report numbers of individuals at each stage of study—eg numbers potentially eligible, examined for eligibility, confirmed eligible, included in the study, completing follow-up, and analysed	5
		(b) Give reasons for non-participation at each stage	5
		(c) Consider use of a flow diagram	-
Descriptive data	14*	(a) Give characteristics of study participants (eg demographic, clinical, social) and information on exposures and potential confounders	Table 1
		(b) Indicate number of participants with missing data for each variable of interest	Table 1
Outcome data	15*	Report numbers of outcome events or summary measures	Table 1
Main results	16	(*a*) Give unadjusted estimates and, if applicable, confounder-adjusted estimates and their precision (eg, 95% confidence interval). Make clear which confounders were adjusted for and why they were included	Table 2 Page 9-11
		(*b*) Report category boundaries when continuous variables were categorized	5-7
		(*c*) If relevant, consider translating estimates of relative risk into absolute risk for a meaningful time period	-
Other analyses	17	Report other analyses done—eg analyses of subgroups and interactions, and sensitivity analyses	10-11
**Discussion**			
Key results	18	Summarise key results with reference to study objectives	11
Limitations	19	Discuss limitations of the study, taking into account sources of potential bias or imprecision. Discuss both direction and magnitude of any potential bias	11-13
Interpretation	20	Give a cautious overall interpretation of results considering objectives, limitations, multiplicity of analyses, results from similar studies, and other relevant evidence	13-14
Generalisability	21	Discuss the generalisability (external validity) of the study results	11
**Other information**			
Funding	22	Give the source of funding and the role of the funders for the present study and, if applicable, for the original study on which the present article is based	15

*Give information separately for cases and controls in case-control studies and, if applicable, for exposed and unexposed groups in cohort and cross-sectional studies.

Note: An Explanation and Elaboration article discusses each checklist item and gives methodological background and published examples of transparent reporting. The STROBE checklist is best used in conjunction with this article (freely available on the Web sites of PLoS Medicine at http://www.plosmedicine.org/, Annals of Internal Medicine at http://www.annals.org/, and Epidemiology at http://www.epidem.com/). Information on the STROBE Initiative is available at www.strobe-statement.org.

For peer review only

BMJ Open

Is there an association between disease ignorance and self-rated health? The HUNT Study, a cross-sectional survey

Journal:	*BMJ Open*
Manuscript ID:	bmjopen-2014-004962.R2
Article Type:	Research
Date Submitted by the Author:	08-May-2014
Complete List of Authors:	Jørgensen, Pål; Norwegian University of Science and Technology, Department of Public Health and General Practice<br>Langhammer, Arnulf; Norwegian University of Science and Technology, HUNT Research Centre<br>Krokstad, Steinar; Norwegian University of Science and Technology, HUNT Research Centre<br>Forsmo, Siri; Norwegian University of Science and Technology, Department of Public Health and General Practice
**Primary Subject Heading**:	General practice / Family practice
Secondary Subject Heading:	Epidemiology, Public health, Diabetes and endocrinology
Keywords:	Hypertension < CARDIOLOGY, General diabetes < DIABETES & ENDOCRINOLOGY, Thyroid disease < DIABETES & ENDOCRINOLOGY, PRIMARY CARE

SCHOLARONE™ Manuscripts

For peer review only

Is there an association between disease ignorance and self-rated health? The HUNT Study, a cross-sectional survey

Pål Jørgensen, Arnulf Langhammer, Steinar Krokstad, Siri Forsmo

Department of Public Health and General Practice, Norwegian University of Science and Technology, 7489 Trondheim, Norway Pål Jørgensen
PhD Candidate
HUNT Research Centre, Department of Public Health and General Practice, Norwegian University of Science and Technology, 7600 Levanger, Norway Arnulf Langhammer
Professor, Head of HUNT Databank
HUNT Research Centre, Department of Public Health and General Practice, Norwegian University of Science and Technology, Steinar Krokstad
Professor, Head of HUNT Research Center
Department of Public Health and General Practice, Norwegian University of Science and Technology, Siri Forsmo
Professor, Head of Department

Correspondence to: P Jørgensen pal_jorgensen@ntnu.no

Keywords: Self-rated health, awareness, hypothyroidism, diabetes, hypertension.

Word count: 3380

For peer review only

ABSTRACT

Objective To explore if awareness versus unawareness of thyroid dysfunction, diabetes mellitus or hypertension is associated with self-rated health.

Design Large-scale, cross sectional population based study. The association between thyroid function, diabetes mellitus, and blood pressure and self-rated health was explored by multiple logistic regression analysis.

Setting The second survey of the Nord-Trøndelag Health Study, HUNT2, 1995-97.

Participants 33,734 persons aged 40-70 years.

Primary outcome measures Logistic regression was used to estimate odds ratios for good self-rated health as a function of thyroid status, diabetes mellitus status and blood pressure status.

Results Persons aware of their hypothyroidism, diabetes mellitus, or hypertension reported poorer self-rated health than individuals without such conditions. Women with unknown and subclinical hypothyroidism reported better self-rated health than women with normal thyroid status. Both in women and men, unknown and probable diabetes, as well as unknown mild/moderate hypertension was not associated with poorer health. Further, persons with unknown severe hypertension reported better health than normotensive persons.

Conclusions People with undiagnosed, but prevalent hypothyroidism, diabetes mellitus, and hypertension often have good self-rated health, whilst when aware of their diagnoses, they report reduced self-rated health. Use of screening, more sensitive tests, and widened diagnostic criteria might have a negative effect on perceived health in the population.

For peer review only

STRENGTHS AND LIMITATIONS OF THIS STUDY

Strengths

- Sample from a large-scale, general population
- High-prevalent diseases under study; ensuring statistical power in subgroup analyses

Limitations

- Study mainly based on self-reported data
- Cross-sectional study; susceptibility to confounding and impossibility to assume causal relationships

For peer review only

INTRODUCTION

Guidelines for prevention and treatment have been developed for most high prevalent diseases in western countries aiming for reduction of morbidity and mortality by interventions mainly in primary health care (PHC).

In the society there seems to be an increasing conviction of achievable zero-vision regarding risks and diseases. Part of the strategy is to detect risk factors and pre-diseases in even earlier stages. From a secondary or tertiary health care level, this might seem reasonable since intervention on many individuals with specific risk factors presumably can prevent or delay disease or progression of disease. Further, health authorities and hospital clinicians regularly raise concern of the lack of detection of risk factors, of subclinical conditions and of achieving treatment goals.[1-7] Norwegian studies have shown that guidelines are often difficult to implement and adhere to in PHC.[8 9] According to guidelines, most individuals would be defined as at risk and resources needed to handle this appropriately could destabilize the entire health care system.[10-12] An American review pointed out knowledge, attitude, and behaviour as barriers of physician's adherence to clinical guidelines.[13] In an already complex and busy PHC-setting, one might expect that resources used for disease prevention and case finding have to compete with resources for handling acknowledged disease. Also, physicians might want to avoid increasing disease related burden for patients, in line with the old wisdom; "primum non nocere".[14] Thus the risk and disease zero-visions in society and among politicians are seldom shared by PHC professionals.

When guidelines, mainly based on research from high risk hospital populations, are applied on low risk populations in PHC, more healthy individuals are identified as being at risk or are given diagnoses. Also the widened inclusion criteria for diagnoses in general and use of more

For peer review only

sensitive tests contribute to define more individuals at risk or as unhealthy.[15] Possible undesirable outcomes of such strategies remain unclear.

Self-rated health (SRH) is a valid and widely used measure of general health in epidemiologic research.[16-19] It is associated with several clinical conditions often seen in PHC, with recovery [20-23] and is found to predict morbidity, sick leave, and disability pension,[24 25] as well as mortality.[26-28] The majority of studies describing association between labelling of disease per se and SRH have focused on arterial hypertension.[29-32] However, one study has indicated reduced SRH among individuals labelled with thyroid dysfunctions.[33]

The aim of this study was to investigate whether persons' awareness versus unawareness of thyroid dysfunction, diabetes mellitus, or hypertension was associated with their SRH, as reported in a population-based health study in Norway.

For peer review only

METHODS

Study population

The data sample in this study stem from the second wave of the Nord-Trøndelag Health Study (HUNT2) conducted in 1995-97 in the county of Nord-Trøndelag, Norway. All individuals aged 20 years and older, living in the county, were invited (94,194 individuals). In all, 66.7% of men (n=30,860) and 75.5% of women (n=35,280) participated. The survey consisted of both questionnaires and measurements, and is described previously in detail.[34 35] In our study we included answers from the main questionnaire and the baseline measurements for persons aged 40-70 years. The age span was chosen because thyroid stimulating hormone (TSH) was analysed in all women and in 50% of men at this age, of a rather low disease burden in people younger than 40 years, and of a lower attendance rate under and above this age span. A total of 24,950 individuals had TSH measurements and answered thyroid questions, thus were eligible for analysis on thyroid dysfunction, whilst in the analysis of diabetes mellitus and blood pressure, 33,734 individuals were included in all.

Self-rated health

The first question in the main questionnaire in HUNT2, answered before attending the examination stations, was "How is your health at the moment?", with answer alternatives; "very good", "good", "not so good", and "poor". This short version of SRH measure is shown to be a valid predictor of mortality. [18 28 36 37] We dichotomized the answers into "good" (very good, good) and "poor" (not so good, poor). Dichotomization of multinomial SRH is commonly performed, and has been validated by Manor et. al.[38]

Thyroid function

For peer review only

The participants answered questions on history of hypo- and hyperthyroidism, goitre, other thyroid diseases, and treatment with thyroxin, radio-iodine, surgery, or thyreostatic medication.

Serum TSH and free T4 were analysed at the Hormone laboratory, Aker University Hospital, Norway. The laboratory reference value for TSH, as defined prior to the survey, was 0.2-4.5 mU/L and for free T_4 8.0-20.0 pmol/L. If TSH was <0.2 mU/L or >4.0 mU/L, and/or if the participant reported any thyroid disease, serum free T_4 was also measured.[39]

Individuals reporting no previous thyroid disease and having TSH within reference range were categorized as “no thyroid disease” and chosen as reference category. No previous thyroid disease combined with TSH >4.5 mU/L and free T_4 <8.0 pmol/L was defined as unknown hypothyroidism. No previous thyroid disease combined with TSH >4.5 mU/L and free T_4 8.0 – 20.0 pmol/L was defined as subclinical hypothyroidism. Individuals reporting hypothyroidism and use of thyroxin were classified as having known hypothyroidism, regardless of the TSH and T4 levels. Affirmative answers to other thyroid related questions or measures outside reference range in the remainders were classified as other thyroid dysfunction.

Diabetes mellitus

Diabetes mellitus was assessed through self-report and blood samples. Serum glucose was analysed at Levanger Hospital, Norway. Those reporting no diabetes and having normal glucose levels (<5.5 mmol/L) were classified as “no diabetes” and were chosen as reference category. No self-reported diabetes and non-fasting glucose >11.0 mmol/L was categorized as unknown diabetes, whereas no diabetes and non-fasting glucose 5.5-11.0 mmol/L was

For peer review only - http://bmjopen.bmj.com/site/about/guidelines.xhtml

categorized as probable diabetes. Self-reported diabetes was classified as known diabetes regardless of the glucose level.

Blood pressure

In the questionnaire participants were asked about the doctor's advice after the latest blood pressure measurement prior to participation in HUNT. The answer categories were: "no follow-up and no medication necessary", "recommended follow-up examination but not to take medicine", "start or continue taking medicine for high blood pressure", or "never measured". At HUNT2, mean systolic and mean diastolic arterial blood pressure (BP) of measurement 2 and 3 was categorized into normal (systolic (s) BP < 140 mmHg and diastolic (d) BP < 90 mmHg), mild hypertension (sBP 140-159 mmHg and dBP <100 mmHg or sBP <160 mmHg and dBP 90-99 mmHg), moderate hypertension (sBP 160-179 mmHg and dBP <109 mmHg or sBP <180 mmHg and dBP 100-109 mmHg), and severe hypertension (sBP>180 mmHg or dBP>110 mmHg). We constructed a new variable to define normotensive (reference), unknown mild- and moderate hypertensive, unknown severe hypertensive, and known hypertensive persons on the basis of self-report and measures.

Statistical analysis

The descriptive analyses of the study population were stratified by gender, and we used chi-square tests to examine any difference in proportions of SRH between the independent variables. Gender-stratified multiple logistic regression were used to estimate odds ratio (OR) with 95% confidence intervals (CI) for good SRH, as a function of thyroid status, diabetes mellitus status and blood pressure status, in separate unadjusted, age adjusted, and multi adjusted analyses for each condition.

For peer review only

Age, smoking, alcohol consumption, body mass index (BMI), working- and educational status, and self-reported limiting long-term illness or injury are associated with SRH[40] and the diseases under study, but likely not affected by SRH or the diseases. Hence, these variables were included, a priori, as confounders in the models. Age was categorized into age groups; 40-49 years, 50-59 years, and 60-70 years. Smoking status was categorized into never smoked daily, previous daily smoker, and current daily smoker. Alcohol units (AU) were defined as number of glasses of wine, beer or liquor. Those reporting to be teetotalers or to have alcohol intake less than four times a month or less than seven AU per two weeks were categorized as low consumers, those reporting drinking five to eight times a month or 8-14 AU per two weeks as moderate consumers, and those drinking more often than eight times a month or more than 14 AU as heavy consumers. BMI ($kg/m^{2)}$ was calculated of measured height and weight and categorized according to The World Health Organization's (WHO) definition; underweight (18.5 kg/m^2), normal weight (18.5-24.9 kg/m^2), overweight (25.0-29.9 kg/m^2) and obese (>30.0 kg/m^2). People reporting paid- or self-employed work were classified as working, otherwise as not working. Educational level was categorized into <10 years, 10-12 years and >12 years. We chose affirmative answer to the question "Do you suffer from any long-term illness or injury (at least one year) of a physical or psychological nature that impairs your functioning in your everyday life?" to represent all relevant chronic medical conditions that could confound the results.

To examine whether the association of the three disease statuses with SRH differed by categories of the other independent variables, we used likelihood ratio-tests with p-value for statistical interaction. We tested for multicollinearity between the independent variables, by linear regression. Areas under the receiver operating characteristic curves (AUC) were calculated to evaluate the performance of the logistic regression models.

For peer review

In an additional analysis, the association between SRH and having had one or more medical consultations during the last year was investigated by logistic regression models, stratified by gender, both in the total study population and after exclusion of persons with diagnoses under study.

All analyses were preformed with IBM SPSS Statistics version 20 for windows.

The study was approved by the Regional Committee for Medical Research. All participants signed written informed consent.

RESULTS

In all age categories, a higher proportion of men than women reported good SRH ($p<0.001$), and in both sexes the proportion reporting good SRH declined by age ($p<0.002$) (Table 1). The proportion reporting good SRH was lower in overweight, obese, and underweight women, than in normal weight women ($p<0.001$). In men the proportion reporting good SRH declined between the normal weight group to overweight-, obese-, and underweight group ($p<0.001$). In previous and current female smokers, a lower proportion reported good SRH compared to non smokers ($p<0.001$). In men, the proportion reporting good SRH was higher among non smokers than among previous and current smokers ($p<0.001$), whereas there was no difference between previous and current smokers. The proportion reporting good SRH was higher in moderate and heavy alcohol consumers than in low consumers, increased with education and was higher for participants in paid work ($p<0.001$). Underweight men and persons with "any long-term impairment" had the lowest proportion reporting good SRH of all groups. The proportion reporting good SRH in the overall HUNT 2 study population differed from the proportions reported in persons without thyroid disease and normotensive persons ($p<0.01$), but not from persons without diabetes mellitus. However, the absolute differences were small.

For peer review only - http://bmjopen.bmj.com/site/about/guidelines.xhtml

In all fully adjusted regression models, the AUC was 0.80. Unadjusted, the AUC ranged from 0.61 to 0.63.

There was no multicollinearity between the independent variables.

Thyroid function

Thyroid dysfunctions, both known and unknown were more often observed among women than among men ($p<0.001$) (Table 1). Women with known hypothyroidism had lower odds of reporting good SRH than women without thyroid disease in the adjusted analyses.However, women with unknown or subclinical hypothyroidism had 84% and 48% higher odds, respectively, of reporting good SRH compared to the odds of women without thyroid disease (Table 2). The association between thyroid function and SRH was basically unchanged after inclusion of confounder variables. Corresponding, but non-significant associations were found among men.

Diabetes mellitus

The prevalence of unknown, probable, and known diabetes was slightly higher in men than in women ($p<0.001$) (Table 1). Women with known diabetes mellitus had lower odds of good SRH than those without diabetes in the adjusted analyses, whereas in women with unknown or possible diabetes mellitus, the odds of good SRH were similar to the odds among persons without diabetes, in the adjusted analyses (Table 2). In the adjusted analyses, the association between unknown and probable diabetes mellitus with poor SRH, found in the crude analyses, disappeared when age was included in the model in women and men, but also by inclusion of working status alone in women. In men, the association of diabetes status with SRH differed by levels of education. Among men without higher education (12 years or less), the odds of good SRH were as in the main-effect model (Table 2). However in men with higher

For peer review only

education, the ORs of good SRH were barely significantly lower among men with unknown diabetes (OR 0.29 (95% CI 0.09-1.00)) and among men with possible diabetes (OR 0.79 (95% CI 0.63-1.00)) compared to men without diabetes. Among men with known diabetes in this stratum, the OR of good SRH was 0.31 (95% CI 0.17-0.54) compared to men without diabetes.

Blood pressure

The prevalence of unknown mild and moderate hypertension was higher in men than in women (p<0.001). Unknown severe hypertension and known hypertension were equally distributed between women and men (Table 1). Women with known hypertension had lower odds of reporting good SRH than normotensive women, in the adjusted analyses. The figures were similar in men (Table 2). In contrast; compared to normotensive women, those with unknown severe hypertension had 52 % higher odds of reporting good SRH, with similar figures in men. Persons with unknown mild and moderate hypertension reported good SRH similar to the normotensive ones. Adjusted for age, the association between unknown mild and moderate hypertension and poor SRH disappeared, simultaneously; unknown severe hypertension became associated with good SRH in women. In men, age had to be added along with either education- or working status to achieve the latter association.

Additional analyses

Women with poor SRH had more than six times the odds of those with good SRH to have had a medical consultation during the last year; OR 6.29 (95% CI 5.47 to 7.22). For men the corresponding OR was 5.53 (95% CI 4.86 to 6.29). After exclusion of persons with diagnosed thyroid disease, diabetes mellitus, known hypertension, and “any long-term disease”, the corresponding OR was 3.71 (95% CI 2.90 to 4.73), with similar figures in men.

For peer review only

DISCUSSION

This large population-based study showed that persons with known thyroid dysfunction, diabetes mellitus, and hypertension were less likely to report good SRH than those without such conditions. Less expected, persons with unknown and subclinical hypothyroidism were more likely to report good SRH than those without thyroid disease. Similarly, those with unknown severe hypertension were more likely to report good SRH, compared to persons with normal blood pressure. In general, persons with unknown diabetes and unknown mild/moderate hypertension, reported good health, just like the reference group.

In general, of the confounders, age seemed to influence the association between disease statuses and SRH most when adjusted for. Age was found to explain the association of poor SRH with unknown and probable diabetes, and with unknown mild and moderate hypertension. In women, age even contributed to an association between unknown severe hypertension and good SRH. There seemed to be a linear decrease by age categories in the association with good SRH. The way age is known to be related to both disease and SRH makes these findings reasonable.

Although both a qualitative and quantitative measure of association, the estimated ORs in our study will, if interpreted as relative risks, overestimate the case, because the prevalence of good SRH is high in all groups.[41]

The main strengths of this study were the numbers of participants, that the diseases we studied were high-prevalent, and that we could assume representativeness to the general population regarding the variables included. It is known that individuals with high burden of symptoms are less likely to attend surveys, but so far there has been little evidence that non-participation

For peer review only

on this basis introduce substantial bias in associational studies.[42] We studied diseases with generally low symptom-burden, and expect selection bias to be negligible.

The main limitation was possibly that we relied on self-reported data on both dependent and independent variables. The validity of self reported measures relevant for this study is questionable. However, if any, there should be a non-differential misclassification, that only should underestimate the associations found.

There is always a possibility of residual confounding in non-randomized study designs, and due to the observational, cross-sectional design we neither can assume a causal relationship.

Bias due to differential detection of disease in the study population could lead to a type 1 error. Hypothyroidism and diabetes mellitus are often associated with vague symptoms such as tiredness and weakness. These symptoms are strongly associated with reduced SRH.[43] It is likely that presenting such symptoms for the GP would result in measurements of TSH, free T4, and serum glucose, thus reveal any related dysfunction. Low self-perceived health increases the probability to visit a GP.[44] The social security covers most costs related to clinical measurements and blood sampling in Norway, thus we expect GPs to have a low threshold to measure thyroid function, blood pressure or glucose levels. People who do not consult their GP will not have diseases with mild or no symptoms diagnosed, and their personality could be characterized by less worrying and more optimistic attitude to health being reflected in better SRH.[45]

On the other hand, the association between known disease and poor SRH could in fact be explained physiologically due to the pathological effect of disease. Lack of corresponding association between ignored, but prevalent disease and poor SRH is not in line with this

For peer review only

hypothesis, rising question of a possible adverse effect of disease labelling. However, confounding by severity of disease cannot be ruled out.

Due to low numbers of persons having unknown hypothyroidism and diabetes mellitus, the results should be interpreted with caution. On the other hand, there were high numbers in the subclinical and probable groups, and analyses of these groups did not show any associations with poor SRH, also rising question of a possible disease labelling effect.

The fact that the HUNT2 survey was carried out nearly 20 years ago raises a question of generalisability to today's population. Stability of SRH over time has not been investigated in our study population, although investigated among adolescents.[46] We do not expect the association between low burden- or subclinical disease with SRH to be time dependent to the extent that it would change our results considerably. Neither do we expect the changes in prevalence of most explanatory variables to influence the associations found.

Consistent findings regarding unknown, subclinical, and probable disease, versus known disease, could indicate a potential adverse effect of disease labelling. Although early detection of disease is protective on morbidity and mortality for many diseases, low SRH is also shown to be associated morbidity and mortality.[25-27] This is an important aspect in the debate of presymptomatic case-finding.

The BMJ's Too Much Medicine campaign aims to highlight the threat to human health posed by overdiagnosis and the waste of resources on unnecessary care (http://www.bmj.com/too-much-medicine). American data have shown that the majority of all health care includes preference-sensitive and supply-sensitive services. The extent of these services varies greatly without necessarily leading to better health.[47 48] A great deal of activity in such services is based on identifying subclinical disease. The negative health effect this might have,

For peer review only

modulated through reduced SRH, can explain why population mortality is not reduced in areas with high frequency of diagnoses.

Both public and academic debates are often characterised by the conviction that all medical treatment is efficient. Wennberg showed that a relatively small proportion of all medical treatment is indisputably good for health.[49] The concern for unrevealed risk factors and subclinical conditions might lead to unnecessary costly health care interventions, increase supply-sensitive services without positive health effect and have a negative influence on people's general and self-perceived health causing more harm than good.[15]

Of ethical reasons, the possible causal effect of disease labelling on SRH is impossible to assess in a randomized controlled trial. Our study emphasizes the need for more prospective research to investigate potential health effects of disease labelling and early diagnosis.

CONCLUSION

Our data suggest that early identification of disease may imply a negative effect on SRH, and to the extent that SRH has been associated with greater mortality, this may lead to harm. However, as it is also known that diseases such as diabetes and hypertension also lead to increased mortality when detected after cardiovascular and metabolic complications have developed, it remains to be seen if an early identification or a late detection strategy would provide optimal health for the population.

For peer review only

FOOTNOTES

Copyright The Corresponding Author has the right to grant on behalf of all authors and does grant on behalf of all authors, a worldwide licence to the Publishers and its licensees in perpetuity, in all forms, formats and media (whether known now or created in the future), to i) publish, reproduce, distribute, display and store the Contribution, ii) translate the Contribution into other languages, create adaptations, reprints, include within collections and create summaries, extracts and/or, abstracts of the Contribution, iii) create any other derivative work(s) based on the Contribution, iv) to exploit all subsidiary rights in the Contribution, v) the inclusion of electronic links from the Contribution to third party material where-ever it may be located; and, vi) licence any third party to do any or all of the above.

Competing interests All authors have completed the ICMJE uniform disclosure form at www.icmje.org/coi_disclosure.pdf and declare: no support from any organisation for the submitted work; no financial relationships with any organisations that might have an interest in the submitted work in the previous three years; no other relationships or activities that could appear to have influenced the submitted work.

Contributors All authors meet the four criteria for authorship recommended by the International Committee of Medical Journal Editors. SF, AL, and SK have been active supervisors in study conception, design, conduct, interpretation, and reporting. PJ analyzed the data and drafted the manuscript. Critical revisions were done by all supervisors and all authors approved the final version of the manuscript. SF, AL, SK and PJ are joint guarantors.

Acknowledgements The Nord-Trøndelag Health Study (The HUNT Study) is a collaboration between HUNT Research Centre (Faculty of Medicine, Norwegian University of Science and Technology NTNU), Nord-Trøndelag County Council, Central Norway Health Authority, and the Norwegian Institute of Public Health. The authors would like to thank Bjørn Olav Åsvold for his contribution in planning of the thyroid-part of the study.

Transparency declaration PJ affirms that this manuscript is an honest, accurate, and transparent account of the study being reported; that no important aspects of the study have been omitted; and that there were no discrepancies from the study as planned.

Ethical approval The study was approved by the Regional Committee for Medical Research. All participants signed written informed consent.

Funding No external funding. PJ received a PhD-grant, funded by the Norwegian University of Science and Technology, NTNU.

Data sharing statement No additional data available.

For peer review only

1. Ziemer DC, Miller CD, Rhee MK, et al. Clinical inertia contributes to poor diabetes control in a primary care setting. Diabetes Educ 2005;**31**(4):564-71 doi: 10.1177/0145721705279050[published Online First: Epub Date]|.
2. Borzecki AM, Wong AT, Hickey EC, et al. Hypertension control: how well are we doing? Arch Intern Med 2003;**163**(22):2705-11 doi: 10.1001/archinte.163.22.2705[published Online First: Epub Date]|.
3. Andros V, Egger A, Dua U. Blood pressure goal attainment according to JNC 7 guidelines and utilization of antihypertensive drug therapy in MCO patients with type 1 or type 2 diabetes. J Manag Care Pharm 2006;**12**(4):303-9
4. Liddy C, Singh J, Hogg W, et al. Quality of cardiovascular disease care in Ontario, Canada: missed opportunities for prevention - a cross sectional study. BMC Cardiovasc Disord 2012;**12**:74 doi: 10.1186/1471-2261-12-74[published Online First: Epub Date]|.
5. Huebschmann AG, Mizrahi T, Soenksen A, et al. Reducing clinical inertia in hypertension treatment: a pragmatic randomized controlled trial. J Clin Hypertens (Greenwich) 2012;**14**(5):322-9 doi: 10.1111/j.1751-7176.2012.00607.x[published Online First: Epub Date]|.
6. Alkerwi Aa, Pagny S, Lair M-L, et al. Level of Unawareness and Management of Diabetes, Hypertension, and Dyslipidemia among Adults in Luxembourg: Findings from ORISCAV-LUX Study. PLoS One 2013;**8**(3):e57920 doi: 10.1371/journal.pone.0057920[published Online First: Epub Date]|.
7. Khan N, Chockalingam A, Campbell NR. Lack of control of high blood pressure and treatment recommendations in Canada. Can J Cardiol 2002;**18**(6):657-61
8. Hetlevik I, Holmen J, Kruger O, et al. Fifteen years with clinical guidelines in the treatment of hypertension - still discrepancies between intentions and practice. Scand J Prim Health Care 1997;**15**(3):134-40 doi: Doi 10.3109/02813439709018503[published Online First: Epub Date]|.
9. Hetlevik I, Holmen J, Midthjell K. Treatment of diabetes mellitus - physicians' adherence to clinical guidelines in Norway. Scand J Prim Health Care 1997;**15**(4):193-97 doi: Doi 10.3109/02813439709035027[published Online First: Epub Date]|.
10. Petursson H, Getz L, Sigurdsson JA, et al. Current European guidelines for management of arterial hypertension: Are they adequate for use in primary care? Modelling study based on the Norwegian HUNT 2 population. BMC Fam Pract 2009;**10** doi: Artn 70 Doi 10.1186/1471-2296-10-70[published Online First: Epub Date]|.
11. Petursson H, Getz L, Sigurdsson JA, et al. Can individuals with a significant risk for cardiovascular disease be adequately identified by combination of several risk factors? Modelling study based on the Norwegian HUNT 2 population. J Eval Clin Pract 2009;**15**(1):103-09 doi: DOI 10.1111/j.1365-2753.2008.00962.x[published Online First: Epub Date]|.
12. Getz L, Sigurdsson JA, Hetlevik I, et al. Estimating the high risk group for cardiovascular disease in the Norwegian HUNT 2 population according to the 2003 European guidelines: modelling study. Br Med J 2005;**331**(7516):551-54A doi: DOI 10.1136/bmj.38555.648623.8F[published Online First: Epub Date]|.
13. Cabana MD, Rand CS, Powe NR, et al. Why don't physicians follow clinical practice guidelines?: A framework for improvement. JAMA 1999;**282**(15):1458-65 doi: 10.1001/jama.282.15.1458[published Online First: Epub Date]|.
14. Smith CM. Origin and uses of primum non nocere--above all, do no harm! J Clin Pharmacol 2005;**45**(4):371-7 doi: 10.1177/0091270004273680[published Online First: Epub Date]|.
15. Moynihan R, Doust J, Henry D. Preventing overdiagnosis: how to stop harming the healthy. Br Med J 2012;**344** doi: Artn E3502 Doi 10.1136/Bmj.E3502[published Online First: Epub Date]|.
16. Miilunpalo S, Vuori I, Oja P, et al. Self-rated health status as a health measure: the predictive value of self-reported health status on the use of physician services and on mortality in the

For peer review only - http://bmjopen.bmj.com/site/about/guidelines.xhtml

working-age population. J Clin Epidemiol 1997;**50**(5):517-28 doi: S0895-4356(97)00045-0 [pii][published Online First: Epub Date]|.

17. Meurer LN, Layde PM, Guse CE. Self-rated health status: a new vital sign for primary care? WMJ 2001;**100**(7):35-9
18. Bowling A. Just one question: If one question works, why ask several? J Epidemiol Community Health 2005;**59**(5):342-5 doi: 59/5/342 [pii] 10.1136/jech.2004.021204[published Online First: Epub Date]|.
19. Lundberg O, Manderbacka K. Assessing reliability of a measure of self-rated health. Scand J Soc Med 1996;**24**(3):218-24
20. Bopp M, Braun J, Gutzwiller F, et al. Health risk or resource? Gradual and independent association between self-rated health and mortality persists over 30 years. PLoS One 2012;**7**(2):e30795 doi: 10.1371/journal.pone.0030795 PONE-D-11-20499[pii][published Online First: Epub Date]|.
21. Moller L, Kristensen TS, Hollnagel H. Self rated health as a predictor of coronary heart disease in Copenhagen, Denmark. J Epidemiol Community Health 1996;**50**(4):423-8
22. Kaplan GA, Goldberg DE, Everson SA, et al. Perceived health status and morbidity and mortality: Evidence from the Kuopio Ischaemic Heart Disease Risk Factor Study. Int J Epidemiol 1996;**25**(2):259-65 doi: Doi 10.1093/Ije/25.2.259[published Online First: Epub Date]|.
23. Krokstad S, Johnsen R, Westin S. Social determinants of disability pension: a 10-year follow-up of 62 000 people in a Norwegian county population. Int J Epidemiol 2002;**31**(6):1183-91 doi: 10.1093/ije/31.6.1183[published Online First: Epub Date]|.
24. Halford C, Wallman T, Welin L, et al. Effects of self-rated health on sick leave, disability pension, hospital admissions and mortality. A population-based longitudinal study of nearly 15,000 observations among Swedish women and men. BMC Public Health 2012;**12**:1103 doi: 10.1186/1471-2458-12-1103[published Online First: Epub Date]|.
25. Latham K, Peek CW. Self-Rated Health and Morbidity Onset Among Late Midlife US Adults. J Gerontol B-Psychol 2013;**68**(1):107-16 doi: DOI 10.1093/geronb/gbs104[published Online First: Epub Date]|.
26. Benyamini Y, Idler EL. Community studies reporting association between self-rated health and mortality - Additional studies, 1995 to 1998. Res Aging 1999;**21**(3):392-401 doi: Doi 10.1177/0164027599213002[published Online First: Epub Date]|.
27. Nielsen AB, Siersma V, Hiort LC, et al. Self-rated general health among 40-year-old Danes and its association with all-cause mortality at 10-, 20-, and 29 years' follow-up. Scand J Public Health 2008;**36**(1):3-11 doi: 10.1177/1403494807085242[published Online First: Epub Date]|.
28. Schou MB, Krokstad S, Westin S. How is self-rated health associated with mortality? Tidsskr Nor Laegeforen 2006;**126**(20):2644-7
29. Barger SD, Muldoon MF. Hypertension labelling was associated with poorer self-rated health in the Third US National Health and Nutrition Examination Survey. J Hum Hypertens 2006;**20**(2):117-23 doi: 10.1038/sj.jhh.1001950[published Online First: Epub Date]|.
30. Bloom JR, Monterossa S. Hypertension Labeling and Sense of Well-Being. Am J Public Health 1981;**71**(11):1228-32 doi: Doi 10.2105/Ajph.71.11.1228[published Online First: Epub Date]|.
31. Moum T, Naess S, Sorensen T, et al. Hypertension Labeling, Life Events and Psychological Well-Being. Psychol Med 1990;**20**(3):635-46
32. Macdonald LA, Sackett DL, Haynes RB, et al. Labelling in hypertension: A review of the behavioural and psychological consequences. J Chronic Dis 1984;**37**(12):933-42 doi: http://dx.doi.org/10.1016/0021-9681(84)90070-5[published Online First: Epub Date]|.
33. Bianchi GP, Zaccheroni V, Solaroli E, et al. Health-related quality of life in patients with thyroid disorders - A study based on Short-Form 36 and Nottingham Health Profile Questionnaires. Qual Life Res 2004;**13**(1):45-54 doi: Doi 10.1023/B:Qure.0000015315.35184.66[published Online First: Epub Date]|.

For peer review only

34. Holmen J, Midthjell K, Krüger Ø, et al. The Nord-Trøndelag Health Study 1995-97 (HUNT 2): objectives, methods, and participation. Norsk Epidemiol 2003 **13** (1):19-32
35. Krokstad S, Langhammer A, Hveem K, et al. Cohort Profile: The HUNT Study, Norway. Int J Epidemiol 2012 doi: dys095 [pii] 10.1093/ije/dys095[published Online First: Epub Date]|.
36. Schnittker J, Bacak V. The Increasing Predictive Validity of Self-Rated Health. PLoS One 2014;**9**(1):e84933 doi: 10.1371/journal.pone.0084933[published Online First: Epub Date]|.
37. Idler EL, Benyamini Y. Self-rated health and mortality: A review of twenty-seven community studies. J Health Soc Behav 1997;**38**(1):21-37 doi: Doi 10.2307/2955359[published Online First: Epub Date]|.
38. Manor O, Matthews S, Power C. Dichotomous or categorical response? Analysing self-rated health and lifetime social class. Int J Epidemiol 2000;**29**(1):149-57 doi: Doi 10.1093/Ije/29.1.149[published Online First: Epub Date]|.
39. Bjoro T, Holmen J, Kruger O, et al. Prevalence of thyroid disease, thyroid dysfunction and thyroid peroxidase antibodies in a large, unselected population. The Health Study of Nord-Trondelag (HUNT). Eur J Endocrinol 2000;**143**(5):639-47 doi: 1430639 [pii][published Online First: Epub Date]|.
40. Giron P. Determinants of self-rated health in Spain: differences by age groups for adults. Eur J Public Health 2012;**22**(1):36-40 doi: 10.1093/eurpub/ckq133[published Online First: Epub Date]|.
41. Davies HTO, Crombie IK, Tavakoli M. When can odds ratios mislead? Br Med J 1998;**316**(7136):989-91
42. Langhammer A, Krokstad S, Romundstad P, et al. The HUNT study: participation is associated with survival and depends on socioeconomic status, diseases and symptoms. BMC Med Res Methodol 2012;**12**:143 doi: 10.1186/1471-2288-12-143[published Online First: Epub Date]|.
43. Molarius A, Janson S. Self-rated health, chronic diseases, and symptoms among middle-aged and elderly men and women. J Clin Epidemiol 2002;**55**(4):364-70 doi: http://dx.doi.org/10.1016/S0895-4356(01)00491-7[published Online First: Epub Date]|.
44. Hansen AH, Halvorsen PA, Ringberg U, et al. Socio-economic inequalities in health care utilisation in Norway: a population based cross-sectional survey. BMC Health Serv Res 2012;**12**:336 doi: 10.1186/1472-6963-12-336[published Online First: Epub Date]|.
45. Vingilis E, Wade T, Seeley J. Predictors of adolescent health care utilization. J Adolesc 2007;**30**(5):773-800 doi: http://dx.doi.org/10.1016/j.adolescence.2006.10.001[published Online First: Epub Date]|.
46. Breidablik H-J, Meland E, Lydersen S. Self-rated health during adolescence: stability and predictors of change (Young-HUNT study, Norway). The European Journal of Public Health 2009;**19**(1):73-78 doi: 10.1093/eurpub/ckn111[published Online First: Epub Date]|.
47. Welch HG, Sharp SM, Gottlieb DJ, et al. Geographic variation in diagnosis frequency and risk of death among Medicare beneficiaries. JAMA 2011;**305**(11):1113-8 doi: 10.1001/jama.2011.307[published Online First: Epub Date]|.
48. Fisher ES, Wennberg DE, Stukel TA, et al. The implications of regional variations in Medicare spending. Part 2: health outcomes and satisfaction with care. Ann Intern Med 2003;**138**(4):288-98
49. Wennberg JE. *Tracking medicine. A researcher's quest to understand health care.* Oxford: Oxford University Press, 2010.

For peer review only

**Study population (*n*= 33,734)**	**Women (*n*=17,514)**			**Men (*n*=16,220)**		
	n	%	SRH good (%)	*n*	%	SRH good (%)
Age group						
40-50 years	7058	40.3	77.6	6517	40.2	81.7
51-60 years	5709	32.6	64.4	5328	32.8	72.1
61-70 years	4747	27.1	56.6	4375	27.0	59.6
BMI (kg/m^2)						
<18,5	6821	0.6	58.4	4804	0.2	42.9
18,5-24,9	7026	39.1	72.8	8694	29.7	75.3
25,0-29,9	3489	40.3	68.5	2635	53.8	73.6
>30	104	20.0	56.5	35	16.3	64.8
(0.4% missing)						
Smoking status						
Never smoked daily	6973	40.1	70.3	4849	30.1	80.0
Previous daily smoker	4651	26.7	68.0	6105	37.9	69.8
Daily smoker	5763	33.1	64.3	5172	32.0	69.1
(0.7% missing)						
Alcohol use						
None to low intake	14888	88.7	66.7	11488	73.2	71.0
Moderate intake	1471	8.8	76.8	2839	18.1	78.7
High intake	430	2.6	76.3	1375	8.8	78.3
(3.7% missing)						
Educational level						
< 10 years	8133	48.5	60.5	5711	36.5	63.1
10 – 12 years	5682	33.9	72.8	6615	42.2	76.0
> 12 years	2962	17.7	80.0	3339	21.3	84.7
(3.8% missing)						
Employed						
Yes	11539	67.2	77.1	12309	77.1	79.9

For peer review only - http://bmjopen.bmj.com/site/about/guidelines.xhtml

No	5627	32.8	49.0		3665	22.9	49.2
(1.8% missing)							
Any long-term impairment							
No	10348	59.1	85.9		9912	63.0	88.7
Yes	6275	35.8	39.8		5829	37.0	46.0
(4.1% missing)							
Thyroid function							
No thyroid disease	14373	86.2	68.7		7804	94.3	72.1
Unknown hypothyroidism	107	0.6	78.5		16	0.2	68.8
Subclinical hypothyroidism	466	2.8	75.0		150	1.8	66.4
Known hypothyroidism	858	5.1	48.9		124	1.5	58.1
Other thyroid dysfunction	872	5.2	61.4		180	2.2	56.2
(1.3% missing)							
Diabetes mellitus							
No diabetes	11279	64.6	69.4		9196	56.9	74.6
Unknown diabetes	46	0.3	52.2		88	0.5	67.8
Probable diabetes	5728	32.8	65.9		6392	39.6	71.5
Known diabetes	395	2.3	43.1		482	3.0	49.5
(0.4% missing)							
Blood pressure status							
Normotensive	9135	52.6	72.4		6858	42.7	77.5
Unknown mild/moderate hypertension	4473	25.8	67.9		5343	33.3	74.7
Unknown severe hypertension	403	2.4	70.7		349	2.2	73.8
Known hypertension	3327	19.2	54.0		3498	21.8	59.6
(0.9% missing)							
Overall study population, HUNT2	34332	97.3	70.3		30378	98.4	74.9

Table 1. Good self-rated health (SRH) by sex and characteristics of the study population.

For peer review only - http://bmjopen.bmj.com/site/about/guidelines.xhtml

Table 2. The association between self-rated health and thyroid function, diabetes mellitus and blood pressure. Odds ratio (OR) of good self-rated health and 95% confidence intervals (95% CI), crude, age adjusted, and adjusted for age, other long-term illness or injury that impairs function in everyday life, smoking habits, alcohol use, educational level, work status and body mass index. Cases with missing data were excluded from the analyses.

	**Women**			**OR (95% CI)**	**Men**			**OR (95% CI)**
	n	Crude	Age adjusted	Multiple adjusted	*n*	Crude	Age adjusted	Multiple adjusted
**Thyroid function**								
No thyroid dysfunction	12476	1.00	1.00	1.00	7045	1.00	1.00	1.00
Unknown hypothyroidism	92	1.66 (1.05-2.64)	2.00 (1.25-3.19)	1.84 (1.02-3.33)	14	0.85 (0.30-2.45)	0.92 (0.31-2.72)	1.28 (0.35-4.65)
Subclinical hypothyroidism	394	1.37 (1.10-1.69)	1.49 (1.20-1.85)	1.48 (1.13-1.94)	128	0.77 (0.54-1.08)	0.88 (0.62-1.24)	1.13 (0.72-1.76)
Known hypothyroidism	718	0.44 (0.38-0.50)	0.48 (0.41-0.55)	0.49 (0.41-0.59)	107	0.54 (0.37-0.77)	0.63 (0.43-0.91)	0.69 (0.44-1.09)
Other thyroid dysfunction	729	0.72 (0.63-0.83)	0.77 (0.67-0.89)	0.77 (0.65-0.93)	163	0.50 (0.37-0.67)	0.52 (0.38-0.71)	0.58 (0.41-0.84)
**Diabetes mellitus**								
No diabetes	9946	1.00	1.00	1.00	8464	1.00	1.00	1.00
Unknown diabetes	39	0.48 (0.27-0.86)	0.61 (0.34-1.10)	0.66 (0.32-1.37)	82	0.72 (0.46-1.13)	0.88 (0.55-1.39)	1.03 (0.59-1.81)
Probable diabetes	4797	0.85 (0.80-0.91)	0.94 (0.88-1.01)	1.01 (0.92-1.10)	5759	0.86 (0.80-0.92)	0.94 (0.88-1.01)	0.99 (0.91-1.08)
Known diabetes	318	0.33 (0.27-0.41)	0.42 (0.34-0.51)	0.53 (0.41-0.69)	395	0.33 (0.28-0.40)	0.42 (0.35-0.51)	0.55 (0.43-0.70)
**Blood pressure**								
No hypertension	8180	1.00	1.00	1.00	6378	1.00	1.00	1.00
Unknown mild/moderate hypertension	3787	0.81 (0.75-0.87)	1.01 (0.93-1.09)	1.10 (0.99-1.22)	4837	0.86 (0.79-0.93)	1.01 (0.92-1.10)	1.01 (0.92-1.12)
Unknown severe hypertension	325	0.92 (0.74-1.15)	1.34 (1.08-1.68)	1.52 (1.14-2.02)	308	0.85 (0.77-1.08)	1.27 (0.99-1.62)	1.48 (1.09-2.02)
Known hypertension	2745	0.45 (0.41-0.49)	0.59 (0.54-0.65)	0.69 (0.61-0.77)	3118	0.43 (0.39-0.47)	0.54 (0.50-0.60)	0.64 (0.57-0.72)

For peer review only

What is already known on this topic

Awareness of thyroid dysfunction, diabetes mellitus, and hypertension is associated with reduced self-rated health

Screening, increasingly sensitive diagnostic tests, and widened diagnostic criteria will define more healthy people as sick

What this study adds

Unawareness of hypothyroidism, diabetes mellitus, or hypertension is not associated with reduced self-rated health

For peer review only

Is there an association between disease ignorance and self-rated health? The HUNT Study, a cross-sectional survey

Pål Jørgensen, Arnulf Langhammer, Steinar Krokstad, Siri Forsmo

Department of Public Health and General Practice, Norwegian University of Science and Technology, 7489 Trondheim, Norway Pål Jørgensen
PhD Candidate
HUNT Research Centre, Department of Public Health and General Practice, Norwegian University of Science and Technology, 7600 Levanger, Norway Arnulf Langhammer
Professor, Head of HUNT Databank
HUNT Research Centre, Department of Public Health and General Practice, Norwegian University of Science and Technology, Steinar Krokstad
Professor, Head of HUNT Research Center
Department of Public Health and General Practice, Norwegian University of Science and Technology, Siri Forsmo
Professor, Head of Department

Correspondence to: P Jørgensen pal_jorgensen@ntnu.no

Keywords: Self-rated health, awareness, hypothyroidism, diabetes, hypertension.

Word count: ~~3359~~3380

For peer review only

ABSTRACT

Objective To explore if awareness versus unawareness of thyroid dysfunction, diabetes mellitus or hypertension is associated with self-rated health.

Design Large-scale, cross sectional population based study. The association between thyroid function, diabetes mellitus, and blood pressure and self-rated health was explored by multiple logistic regression analysis.

Setting The second survey of the Nord-Trøndelag Health Study, HUNT2, 1995-97.

Participants 33,734 persons aged 40-70 years.

Primary outcome measures Logistic regression was used to estimate odds ratios for good self-rated health as a function of thyroid status, diabetes mellitus status and blood pressure status.

Results Persons aware of their hypothyroidism, diabetes mellitus, or hypertension reported poorer self-rated health than individuals without such conditions. Women with unknown and subclinical hypothyroidism reported better self-rated health than women with normal thyroid status. Both in women and men, unknown and probable diabetes, as well as unknown mild/moderate hypertension was not associated with poorer health. Further, persons with unknown severe hypertension reported better health than normotensive persons.

Conclusions People with undiagnosed, but prevalent hypothyroidism, diabetes mellitus, and hypertension often have good self-rated health, whilst when aware of their diagnoses, they report reduced self-rated health. Use of screening, more sensitive tests, and widened diagnostic criteria might have a negative effect on perceived health in the population.

For peer review only

STRENGTHS AND LIMITATIONS OF THIS STUDY

Strengths

- Sample from a large-scale, general population
- High-prevalent diseases under study; ensuring statistical power in subgroup analyses

Limitations

- Study mainly based on self-reported data
- Cross-sectional study; susceptibility to confounding and impossibility to assume causal relationships

INTRODUCTION

Guidelines for prevention and treatment have been developed for most high prevalent diseases in western countries aiming for reduction of morbidity and mortality by interventions mainly in primary health care (PHC).

In the society there seems to be an increasing conviction of achievable zero-vision regarding risks and diseases. Part of the strategy is to detect risk factors and pre-diseases in even earlier stages. From a secondary or tertiary health care level, this might seem reasonable since intervention on many individuals with specific risk factors presumably can prevent or delay disease or progression of disease. Further, health authorities and hospital clinicians regularly raise concern of the lack of detection of risk factors, of subclinical conditions and of achieving treatment goals.[1-7] Norwegian studies have shown that guidelines are often difficult to implement and adhere to in PHC.[8 9] According to guidelines, most individuals would be defined as at risk and resources needed to handle this appropriately could destabilize the entire health care system.[10-12] An American review pointed out knowledge, attitude,

For peer review only

and behaviour as barriers of physician's adherence to clinical guidelines.[13] In an already complex and busy PHC-setting, one might expect that resources used for disease prevention and case finding have to compete with resources for handling acknowledged disease. Also, physicians might want to avoid increasing disease related burden for patients, in line with the old wisdom; "primum non nocere".[14] Thus the risk and disease zero-visions in society and among politicians are seldom shared by PHC professionals.

When guidelines, mainly based on research from high risk hospital populations, are applied on low risk populations in PHC, more healthy individuals are identified as being at risk or are given diagnoses. Also the widened inclusion criteria for diagnoses in general and use of more sensitive tests contribute to define more individuals at risk or as unhealthy.[15] Possible undesirable outcomes of such strategies remain unclear.

Self-rated health (SRH) is a valid and widely used measure of general health in epidemiologic research.[16-19] It is associated with several clinical conditions often seen in PHC, with recovery [20-23] and is found to predict morbidity, sick leave, and disability pension,[24 25] as well as mortality.[26-28] The majority of studies describing association between labelling of disease per se and SRH have focused on arterial hypertension.[29-32] However, one study has indicated reduced SRH among individuals labelled with thyroid dysfunctions.[33]

The aim of this study was to investigate whether persons' awareness versus unawareness of thyroid dysfunction, diabetes mellitus, or hypertension was associated with their SRH, as reported in a population-based health study in Norway.

For peer review only

METHODS

Study population

The data sample in this study stem from the second wave of the Nord-Trøndelag Health Study (HUNT2) conducted in 1995-97 in the county of Nord-Trøndelag, Norway. All individuals aged 20 years and older, living in the county, were invited (94,194 individuals). In all, 66.7% of men (n=30,860) and 75.5% of women (n=35,280) participated. The survey consisted of both questionnaires and measurements, and is described previously in detail.[34 35] In our study we included answers from the main questionnaire and the baseline measurements for persons aged 40-70 years. The age span was chosen because thyroid stimulating hormone (TSH) was analysed in all women and in 50% of men at this age, of a rather low disease burden in people younger than 40 years, and of a lower attendance rate under and above this age span. A total of 24,950 individuals had TSH measurements and answered thyroid questions, thus were eligible for analysis on thyroid dysfunction, whilst in the analysis of diabetes mellitus and blood pressure, 33,734 individuals were included in all.

Self-rated health

The first question in the main questionnaire in HUNT2, answered before attending the examination stations, was "How is your health at the moment?", with answer alternatives; "very good", "good", "not so good", and "poor". This short version of SRH measure is shown to be a valid predictor of mortality. [18 28 36 37] We dichotomized the answers into "good" (very good, good) and "poor" (not so good, poor). Dichotomization of multinomial SRH is commonly performed, and has been validated by Manor et. al.[38]

Thyroid function

The participants answered questions on history of hypo- and hyperthyroidism, goitre, other thyroid diseases, and treatment with thyroxin, radio-iodine, surgery, or thyreostatic medication.

Serum TSH and free T4 were analysed at the Hormone laboratory, Aker University Hospital, Norway. The laboratory reference value for TSH, as defined prior to the survey, was 0.2-4.5 mU/L and for free T_4 8.0-20.0 pmol/L. If TSH was <0.2 mU/L or >4.0 mU/L, and/or if the participant reported any thyroid disease, serum free T_4 was also measured.[39]

Individuals reporting no previous thyroid disease and having TSH within reference range were categorized as "no thyroid disease" and chosen as reference category. No previous thyroid disease combined with TSH >4.5 mU/L and free T_4 <8.0 pmol/L was defined as unknown hypothyroidism. No previous thyroid disease combined with TSH >4.5 mU/L and free T_4 8.0 – 20.0 pmol/L was defined as subclinical hypothyroidism. Individuals reporting hypothyroidism and use of thyroxin were classified as having known hypothyroidism, regardless of the TSH and T4 levels. Affirmative answers to other thyroid related questions or measures outside reference range in the remainders were classified as other thyroid dysfunction.

Diabetes mellitus

Diabetes mellitus was assessed through self-report and blood samples. Serum glucose was analysed at Levanger Hospital, Norway. Those reporting no diabetes and having normal glucose levels (<5.5 mmol/L) were classified as "no diabetes" and were chosen as reference category. No self-reported diabetes and non-fasting glucose >11.0 mmol/L was categorized as unknown diabetes, whereas no diabetes and non-fasting glucose 5.5-11.0 mmol/L was

For peer review only

categorized as probable diabetes. Self-reported diabetes was classified as known diabetes regardless of the glucose level.

Blood pressure

In the questionnaire participants were asked about the doctor's advice after the latest blood pressure measurement prior to participation in HUNT. The answer categories were: "no follow-up and no medication necessary", "recommended follow-up examination but not to take medicine", "start or continue taking medicine for high blood pressure", or "never measured". At HUNT2, mean systolic and mean diastolic arterial blood pressure (BP) of measurement 2 and 3 was categorized into normal (systolic (s) BP < 140 mmHg and diastolic (d) BP < 90 mmHg), mild hypertension (sBP 140-159 mmHg and dBP <100 mmHg or sBP <160 mmHg and dBP 90-99 mmHg), moderate hypertension (sBP 160-179 mmHg and dBP <109 mmHg or sBP <180 mmHg and dBP 100-109 mmHg), and severe hypertension (sBP>180 mmHg or dBP>110 mmHg). We constructed a new variable to define normotensive (reference), unknown mild- and moderate hypertensive, unknown severe hypertensive, and known hypertensive persons on the basis of self-report and measures.

Statistical analysis

The descriptive analyses of the study population were stratified by gender, and we used chi-square tests to examine any difference in proportions of SRH between the independent variables. Gender-stratified multiple logistic regression were used to estimate odds ratio (OR) with 95% confidence intervals (CI) for good SRH, as a function of thyroid status, diabetes mellitus status and blood pressure status, in separate unadjusted, age adjusted, and multi adjusted analyses for each condition.

For peer review only

Age, smoking, alcohol consumption, body mass index (BMI), working- and educational status, and self-reported limiting long-term illness or injury are associated with SRH[40] and the diseases under study, but likely not affected by SRH or the diseases. Hence, these variables were included, a priori, as confounders in the models. Age was categorized into age groups; 40-49 years, 50-59 years, and 60-70 years. Smoking status was categorized into never smoked daily, previous daily smoker, and current daily smoker. Alcohol units (AU) were defined as number of glasses of wine, beer or liquor. Those reporting to be teetotalers or to have alcohol intake less than four times a month or less than seven AU per two weeks were categorized as low consumers, those reporting drinking five to eight times a month or 8-14 AU per two weeks as moderate consumers, and those drinking more often than eight times a month or more than 14 AU as heavy consumers. BMI ($kg/m^{2)}$ was calculated of measured height and weight and categorized according to The World Health Organization's (WHO) definition; underweight (18.5 kg/m^2), normal weight (18.5-24.9 kg/m^2), overweight (25.0-29.9 kg/m^2) and obese (>30.0 kg/m^2). People reporting paid- or self-employed work were classified as working, otherwise as not working. Educational level was categorized into <10 years, 10-12 years and >12 years. We chose affirmative answer to the question "Do you suffer from any long-term illness or injury (at least one year) of a physical or psychological nature that impairs your functioning in your everyday life?" to represent all relevant chronic medical conditions that could confound the results.

To examine whether the association of the three disease statuses with SRH differed by categories of the other independent variables, we used likelihood ratio-tests with p-value for statistical interaction. We tested for multicollinearity between the independent variables, by linear regression. Areas under the receiver operating characteristic curves (AUC) were calculated to evaluate the performance of the logistic regression models.

For peer review only - http://bmjopen.bmj.com/site/about/guidelines.xhtml

In an additional analysis, the association between SRH and having had one or more medical consultations during the last year was investigated by logistic regression models, stratified by gender, both in the total study population and after exclusion of persons with diagnoses under study.

All analyses were preformed with IBM SPSS Statistics version 20 for windows.

The study was approved by the Regional Committee for Medical Research. All participants signed written informed consent.

RESULTS

In all age categories, a higher proportion of men than women reported good SRH ($p<0.001$), and in both sexes the proportion reporting good SRH declined by age ($p<0.002$) (Table 1). The proportion reporting good SRH was lower in overweight, obese, and underweight women, than in normal weight women ($p<0.001$). In men the proportion reporting good SRH declined between the normal weight group to overweight-, obese-, and underweight group ($p<0.001$). In previous and current female smokers, a lower proportion reported good SRH compared to non smokers ($p<0.001$). In men, the proportion reporting good SRH was higher among non smokers than among previous and current smokers ($p<0.001$), whereas there was no difference between previous and current smokers. The proportion reporting good SRH was higher in moderate and heavy alcohol consumers than in low consumers, increased with education and was higher for participants in paid work ($p<0.001$). Underweight men and persons with “any long-term impairment” had the lowest proportion reporting good SRH of all groups. The proportion reporting good SRH in the overall HUNT 2 study population differed from the proportions reported in persons without thyroid disease and normotensive persons ($p<0.01$), but not from persons without diabetes mellitus. However, the absolute differences were small.

For peer review only

In all fully adjusted regression models, the AUC was 0.80. Unadjusted, the AUC ranged from 0.61 to 0.63.

There was no multicollinearity between the independent variables.

Thyroid function

Thyroid dysfunctions, both known and unknown were more often observed among women than among men (p<0.001) (Table 1). Women with known hypothyroidism had lower odds of reporting good SRH ~~(OR 0.49 (95% CI 0.41 to 0.59) compared to~~than women without thyroid disease in the adjusted analyses.~~, but~~However, women with unknown or subclinical hypothyroidism had 84% and 48% higher odds, respectively, of reporting good SRH compared to the odds of women without thyroid disease (Table 2). The association between thyroid function and SRH was basically unchanged after inclusion of confounder variables. Corresponding, but non-significant associations were found among men.

Diabetes mellitus

The prevalence of unknown, probable, and known diabetes was slightly higher in men than in women (p<0.001) (Table 1). Women with known diabetes mellitus had lower odds of good SRH than those without diabetes in the adjusted analyses~~; OR 0.53 (95% CI 0.41 to 0.69)~~, whereas in women with unknown or possible diabetes mellitus, the odds of good SRH were similar to the odds among persons without diabetes, in the adjusted analyses (Table 2). In the adjusted analyses, the association between unknown and probable diabetes mellitus with poor SRH, found in the crude analyses, disappeared when age was included in the model in women and men, but also by inclusion of working status alone in women. In men, the association of diabetes status with SRH differed by levels of education. Among men without higher education (12 years or less), the odds of good SRH were as in the main-effect model (Table

For peer review only

2). However in men with higher education, the ORs of good SRH were barely significantly lower among men with unknown diabetes (OR 0.29 (95% CI 0.09-1.00)) and among men with possible diabetes (OR 0.79 (95% CI 0.63-1.00)) compared to men without diabetes. Among men with known diabetes in this stratum, the OR of good SRH was 0.31 (95% CI 0.17-0.54) compared to men without diabetes.

Blood pressure

The prevalence of unknown mild and moderate hypertension was higher in men than in women ($p<0.001$). Unknown severe hypertension and known hypertension were equally distributed between women and men (Table 1). Women with known hypertension had lower odds of reporting good SRH than normotensive women, in the adjusted analyses. The figures were similar in men (Table 2). In contrast; compared to normotensive women, those with unknown severe hypertension had 52 % higher odds of reporting good SRH, with similar figures in men. Persons with unknown mild and moderate hypertension reported good SRH similar to the normotensive ones. Adjusted for age, the association between unknown mild and moderate hypertension and poor SRH disappeared, simultaneously; unknown severe hypertension became associated with good SRH in women. In men, age had to be added along with either education- or working status to achieve the latter association.

Additional analyses

Women with poor SRH had more than six times the odds of those with good SRH to have had a medical consultation during the last year; OR 6.29 (95% CI 5.47 to 7.22). For men the corresponding OR was 5.53 (95% CI 4.86 to 6.29). After exclusion of persons with diagnosed thyroid disease, diabetes mellitus, known hypertension, and “any long-term disease”, the corresponding OR was 3.71 (95% CI 2.90 to 4.73), with similar figures in men.

For peer review only

DISCUSSION

This large population-based study showed that persons with known thyroid dysfunction, diabetes mellitus, and hypertension were less likely to report good SRH than those without such conditions. Less expected, persons with unknown and subclinical hypothyroidism were more likely to report good SRH than those without thyroid disease. Similarly, those with unknown severe hypertension were more likely to report good SRH, compared to persons with normal blood pressure. In general, persons with unknown diabetes and unknown mild/moderate hypertension, reported good health, just like the reference group.

In general, of the confounders, age seemed to influence the association between disease statuses and SRH most when adjusted for. Age was found to explain the association of poor SRH with unknown and probable diabetes, and with unknown mild and moderate hypertension. In women, age even contributed to an association between unknown severe hypertension and good SRH. There seemed to be a linear decrease by age categories in the association with good SRH. The way age is known to be related to both disease and SRH makes these findings reasonable.

Although both a qualitative and quantitative measure of association, the estimated ORs in our study will, if interpreted as relative risks, overestimate the case, because the prevalence of good SRH is high in all groups.[41]

The main strengths of this study were the numbers of participants, that the diseases we studied were high-prevalent, and that we could assume representativeness to the general population regarding the variables included. It is known that individuals with high burden of symptoms are less likely to attend surveys, but so far there has been little evidence that non-participation

For peer review only

on this basis introduce substantial bias in associational studies.[42] We studied diseases with generally low symptom-burden, and expect selection bias to be negligible.

The main limitation was possibly that we relied on self-reported data on both dependent and independent variables. The validity of self reported measures relevant for this study is questionable. However, if any, there should be a non-differential misclassification, that only should underestimate the associations found.

There is always a possibility of residual confounding in non-randomized study designs, and due to the observational, cross-sectional design we neither can assume a causal relationship.

Bias due to differential detection of disease in the study population could lead to a type 1 error. Hypothyroidism and diabetes mellitus are often associated with vague symptoms such as tiredness and weakness. These symptoms are strongly associated with reduced SRH.[43] It is likely that presenting such symptoms for the GP would result in measurements of TSH, free T4, and serum glucose, thus reveal any related dysfunction. Low self-perceived health increases the probability to visit a GP.[44] The social security covers most costs related to clinical measurements and blood sampling in Norway, thus we expect GPs to have a low threshold to measure thyroid function, blood pressure or glucose levels. People who do not consult their GP will not have diseases with mild or no symptoms diagnosed, and their personality could be characterized by less worrying and more optimistic attitude to health being reflected in better SRH.[45]

On the other hand, the association between known disease and poor SRH could in fact be explained physiologically due to the pathological effect of disease. Lack of corresponding association between ignored, but prevalent disease and poor SRH is not in line with this

For peer review only

hypothesis, rising question of a possible adverse effect of disease labelling. However, confounding by severity of disease cannot be ruled out.

Due to low numbers of persons having unknown hypothyroidism and diabetes mellitus, the results should be interpreted with caution. On the other hand, there were high numbers in the subclinical and probable groups, and analyses of these groups did not show any associations with poor SRH, also rising question of a possible disease labelling effect.

The fact that the HUNT2 survey was carried out nearly 20 years ago raises a question of generalisability to today's population. Stability of SRH over time has not been investigated in our study population, although investigated among adolescents.[46] We do not expect the association between low burden- or subclinical disease with SRH to be time dependent to the extent that it would change our results considerably. Neither do we expect the changes in prevalence of most explanatory variables to influence the associations found.

Consistent findings regarding unknown, subclinical, and probable disease, versus known disease, could indicate a potential adverse effect of disease labelling. Although early detection of disease is protective on morbidity and mortality for many diseases, low SRH is also shown to be associated morbidity and mortality.[25-27] This is an important aspect in the debate of presymptomatic case-finding.

The BMJ's Too Much Medicine campaign aims to highlight the threat to human health posed by overdiagnosis and the waste of resources on unnecessary care (http://www.bmj.com/too-much-medicine). American data have shown that the majority of all health care includes preference-sensitive and supply-sensitive services. The extent of these services varies greatly without necessarily leading to better health.[47 48] A great deal of activity in such services is based on identifying subclinical disease. The negative health effect this might have,

For peer review only

modulated through reduced SRH, can explain why population mortality is not reduced in areas with high frequency of diagnoses.

Both public and academic debates are often characterised by the conviction that all medical treatment is efficient. Wennberg showed that a relatively small proportion of all medical treatment is indisputably good for health.[49] The concern for unrevealed risk factors and subclinical conditions might lead to unnecessary costly health care interventions, increase supply-sensitive services without positive health effect and have a negative influence on people's general and self-perceived health causing more harm than good.[15]

Of ethical reasons, the possible causal effect of disease labelling on SRH is impossible to assess in a randomized controlled trial. Our study emphasizes the need for more prospective research to investigate potential health effects of disease labelling and early diagnosis.

CONCLUSION

Our data suggest that early identification of disease may imply a negative effect on SRH, and to the extent that SRH has been associated with greater mortality, this may lead to harm. However, as it is also known that diseases such as diabetes and hypertension also lead to increased mortality when detected after cardiovascular and metabolic complications have developed, it remains to be seen if an early identification or a late detection strategy would provide optimal health for the population.

FOOTNOTES

Copyright The Corresponding Author has the right to grant on behalf of all authors and does grant on behalf of all authors, a worldwide licence to the Publishers and its licensees in perpetuity, in all forms, formats and media (whether known now or created in the future), to i) publish, reproduce, distribute, display and store the Contribution, ii) translate the Contribution into other languages, create adaptations, reprints, include within collections and create summaries, extracts and/or, abstracts of the Contribution, iii) create any other derivative

For peer review only

work(s) based on the Contribution, iv) to exploit all subsidiary rights in the Contribution, v) the inclusion of electronic links from the Contribution to third party material where-ever it may be located; and, vi) licence any third party to do any or all of the above.

Competing interests All authors have completed the ICMJE uniform disclosure form at www.icmje.org/coi_disclosure.pdf and declare: no support from any organisation for the submitted work; no financial relationships with any organisations that might have an interest in the submitted work in the previous three years; no other relationships or activities that could appear to have influenced the submitted work.

Contributors All authors meet the four criteria for authorship recommended by the International Committee of Medical Journal Editors. SF, AL, and SK have been active supervisors in study conception, design, conduct, interpretation, and reporting. PJ analyzed the data and drafted the manuscript. Critical revisions were done by all supervisors and all authors approved the final version of the manuscript. SF, AL, SK and PJ are joint guarantors.

Acknowledgements The Nord-Trøndelag Health Study (The HUNT Study) is a collaboration between HUNT Research Centre (Faculty of Medicine, Norwegian University of Science and Technology NTNU), Nord-Trøndelag County Council, Central Norway Health Authority, and the Norwegian Institute of Public Health. The authors would like to thank Bjørn Olav Åsvold for his contribution in planning of the thyroid-part of the study.

Transparency declaration PJ affirms that this manuscript is an honest, accurate, and transparent account of the study being reported; that no important aspects of the study have been omitted; and that there were no discrepancies from the study as planned.

Ethical approval The study was approved by the Regional Committee for Medical Research. All participants signed written informed consent.

Funding No external funding. PJ received a PhD-grant, funded by the Norwegian University of Science and Technology, NTNU.

Data sharing statement No additional data available.

For peer review only

1. Ziemer DC, Miller CD, Rhee MK, et al. Clinical inertia contributes to poor diabetes control in a primary care setting. Diabetes Educ 2005;**31**(4):564-71 doi: 10.1177/0145721705279050[published Online First: Epub Date]|.
2. Borzecki AM, Wong AT, Hickey EC, et al. Hypertension control: how well are we doing? Arch Intern Med 2003;**163**(22):2705-11 doi: 10.1001/archinte.163.22.2705[published Online First: Epub Date]|.
3. Andros V, Egger A, Dua U. Blood pressure goal attainment according to JNC 7 guidelines and utilization of antihypertensive drug therapy in MCO patients with type 1 or type 2 diabetes. J Manag Care Pharm 2006;**12**(4):303-9
4. Liddy C, Singh J, Hogg W, et al. Quality of cardiovascular disease care in Ontario, Canada: missed opportunities for prevention - a cross sectional study. BMC Cardiovasc Disord 2012;**12**:74 doi: 10.1186/1471-2261-12-74[published Online First: Epub Date]|.
5. Huebschmann AG, Mizrahi T, Soenksen A, et al. Reducing clinical inertia in hypertension treatment: a pragmatic randomized controlled trial. J Clin Hypertens (Greenwich) 2012;**14**(5):322-9 doi: 10.1111/j.1751-7176.2012.00607.x[published Online First: Epub Date]|.
6. Alkerwi Aa, Pagny S, Lair M-L, et al. Level of Unawareness and Management of Diabetes, Hypertension, and Dyslipidemia among Adults in Luxembourg: Findings from ORISCAV-LUX Study. PLoS One 2013;**8**(3):e57920 doi: 10.1371/journal.pone.0057920[published Online First: Epub Date]|.
7. Khan N, Chockalingam A, Campbell NR. Lack of control of high blood pressure and treatment recommendations in Canada. Can J Cardiol 2002;**18**(6):657-61
8. Hetlevik I, Holmen J, Kruger O, et al. Fifteen years with clinical guidelines in the treatment of hypertension - still discrepancies between intentions and practice. Scand J Prim Health Care 1997;**15**(3):134-40 doi: Doi 10.3109/02813439709018503[published Online First: Epub Date]|.
9. Hetlevik I, Holmen J, Midthjell K. Treatment of diabetes mellitus - physicians' adherence to clinical guidelines in Norway. Scand J Prim Health Care 1997;**15**(4):193-97 doi: Doi 10.3109/02813439709035027[published Online First: Epub Date]|.
10. Petursson H, Getz L, Sigurdsson JA, et al. Current European guidelines for management of arterial hypertension: Are they adequate for use in primary care? Modelling study based on the Norwegian HUNT 2 population. BMC Fam Pract 2009;**10** doi: Artn 70 Doi 10.1186/1471-2296-10-70[published Online First: Epub Date]|.
11. Petursson H, Getz L, Sigurdsson JA, et al. Can individuals with a significant risk for cardiovascular disease be adequately identified by combination of several risk factors? Modelling study based on the Norwegian HUNT 2 population. J Eval Clin Pract 2009;**15**(1):103-09 doi: DOI 10.1111/j.1365-2753.2008.00962.x[published Online First: Epub Date]|.
12. Getz L, Sigurdsson JA, Hetlevik I, et al. Estimating the high risk group for cardiovascular disease in the Norwegian HUNT 2 population according to the 2003 European guidelines: modelling study. Br Med J 2005;**331**(7516):551-54A doi: DOI 10.1136/bmj.38555.648623.8F[published Online First: Epub Date]|.
13. Cabana MD, Rand CS, Powe NR, et al. Why don't physicians follow clinical practice guidelines?: A framework for improvement. JAMA 1999;**282**(15):1458-65 doi: 10.1001/jama.282.15.1458[published Online First: Epub Date]|.
14. Smith CM. Origin and uses of primum non nocere--above all, do no harm! J Clin Pharmacol 2005;**45**(4):371-7 doi: 10.1177/0091270004273680[published Online First: Epub Date]|.
15. Moynihan R, Doust J, Henry D. Preventing overdiagnosis: how to stop harming the healthy. Br Med J 2012;**344** doi: Artn E3502 Doi 10.1136/Bmj.E3502[published Online First: Epub Date]|.
16. Miilunpalo S, Vuori I, Oja P, et al. Self-rated health status as a health measure: the predictive value of self-reported health status on the use of physician services and on mortality in the

For peer review only

working-age population. J Clin Epidemiol 1997;**50**(5):517-28 doi: S0895-4356(97)00045-0 [pii][published Online First: Epub Date]|.

17. Meurer LN, Layde PM, Guse CE. Self-rated health status: a new vital sign for primary care? WMJ 2001;**100**(7):35-9
18. Bowling A. Just one question: If one question works, why ask several? J Epidemiol Community Health 2005;**59**(5):342-5 doi: 59/5/342 [pii] 10.1136/jech.2004.021204[published Online First: Epub Date]|.
19. Lundberg O, Manderbacka K. Assessing reliability of a measure of self-rated health. Scand J Soc Med 1996;**24**(3):218-24
20. Bopp M, Braun J, Gutzwiller F, et al. Health risk or resource? Gradual and independent association between self-rated health and mortality persists over 30 years. PLoS One 2012;**7**(2):e30795 doi: 10.1371/journal.pone.0030795 PONE-D-11-20499[pii][published Online First: Epub Date]|.
21. Moller L, Kristensen TS, Hollnagel H. Self rated health as a predictor of coronary heart disease in Copenhagen, Denmark. J Epidemiol Community Health 1996;**50**(4):423-8
22. Kaplan GA, Goldberg DE, Everson SA, et al. Perceived health status and morbidity and mortality: Evidence from the Kuopio Ischaemic Heart Disease Risk Factor Study. Int J Epidemiol 1996;**25**(2):259-65 doi: Doi 10.1093/Ije/25.2.259[published Online First: Epub Date]|.
23. Krokstad S, Johnsen R, Westin S. Social determinants of disability pension: a 10-year follow-up of 62 000 people in a Norwegian county population. Int J Epidemiol 2002;**31**(6):1183-91 doi: 10.1093/ije/31.6.1183[published Online First: Epub Date]|.
24. Halford C, Wallman T, Welin L, et al. Effects of self-rated health on sick leave, disability pension, hospital admissions and mortality. A population-based longitudinal study of nearly 15,000 observations among Swedish women and men. BMC Public Health 2012;**12**:1103 doi: 10.1186/1471-2458-12-1103[published Online First: Epub Date]|.
25. Latham K, Peek CW. Self-Rated Health and Morbidity Onset Among Late Midlife US Adults. J Gerontol B-Psychol 2013;**68**(1):107-16 doi: DOI 10.1093/geronb/gbs104[published Online First: Epub Date]|.
26. Benyamini Y, Idler EL. Community studies reporting association between self-rated health and mortality - Additional studies, 1995 to 1998. Res Aging 1999;**21**(3):392-401 doi: Doi 10.1177/0164027599213002[published Online First: Epub Date]|.
27. Nielsen AB, Siersma V, Hiort LC, et al. Self-rated general health among 40-year-old Danes and its association with all-cause mortality at 10-, 20-, and 29 years' follow-up. Scand J Public Health 2008;**36**(1):3-11 doi: 10.1177/1403494807085242[published Online First: Epub Date]|.
28. Schou MB, Krokstad S, Westin S. How is self-rated health associated with mortality? Tidsskr Nor Laegeforen 2006;**126**(20):2644-7
29. Barger SD, Muldoon MF. Hypertension labelling was associated with poorer self-rated health in the Third US National Health and Nutrition Examination Survey. J Hum Hypertens 2006;**20**(2):117-23 doi: 10.1038/sj.jhh.1001950[published Online First: Epub Date]|.
30. Bloom JR, Monterossa S. Hypertension Labeling and Sense of Well-Being. Am J Public Health 1981;**71**(11):1228-32 doi: Doi 10.2105/Ajph.71.11.1228[published Online First: Epub Date]|.
31. Moum T, Naess S, Sorensen T, et al. Hypertension Labeling, Life Events and Psychological Well-Being. Psychol Med 1990;**20**(3):635-46
32. Macdonald LA, Sackett DL, Haynes RB, et al. Labelling in hypertension: A review of the behavioural and psychological consequences. J Chronic Dis 1984;**37**(12):933-42 doi: http://dx.doi.org/10.1016/0021-9681(84)90070-5[published Online First: Epub Date]|.
33. Bianchi GP, Zaccheroni V, Solaroli E, et al. Health-related quality of life in patients with thyroid disorders - A study based on Short-Form 36 and Nottingham Health Profile Questionnaires. Qual Life Res 2004;**13**(1):45-54 doi: Doi 10.1023/B:Qure.0000015315.35184.66[published Online First: Epub Date]|.

For peer review only

**Study population**	**Women (*n*=17,514)**		**Men (*n*=16,220)**

34. Holmen J, Midthjell K, Krüger Ø, et al. The Nord-Trøndelag Health Study 1995-97 (HUNT 2): objectives, methods, and participation. Norsk Epidemiol 2003 **13** (1):19-32
35. Krokstad S, Langhammer A, Hveem K, et al. Cohort Profile: The HUNT Study, Norway. Int J Epidemiol 2012 doi: dys095 [pii] 10.1093/ije/dys095[published Online First: Epub Date]|.
36. Schnittker J, Bacak V. The Increasing Predictive Validity of Self-Rated Health. PLoS One 2014;**9**(1):e84933 doi: 10.1371/journal.pone.0084933[published Online First: Epub Date]|.
37. Idler EL, Benyamini Y. Self-rated health and mortality: A review of twenty-seven community studies. J Health Soc Behav 1997;**38**(1):21-37 doi: Doi 10.2307/2955359[published Online First: Epub Date]|.
38. Manor O, Matthews S, Power C. Dichotomous or categorical response? Analysing self-rated health and lifetime social class. Int J Epidemiol 2000;**29**(1):149-57 doi: Doi 10.1093/Ije/29.1.149[published Online First: Epub Date]|.
39. Bjoro T, Holmen J, Kruger O, et al. Prevalence of thyroid disease, thyroid dysfunction and thyroid peroxidase antibodies in a large, unselected population. The Health Study of Nord-Trondelag (HUNT). Eur J Endocrinol 2000;**143**(5):639-47 doi: 1430639 [pii][published Online First: Epub Date]|.
40. Giron P. Determinants of self-rated health in Spain: differences by age groups for adults. Eur J Public Health 2012;**22**(1):36-40 doi: 10.1093/eurpub/ckq133[published Online First: Epub Date]|.
41. Davies HTO, Crombie IK, Tavakoli M. When can odds ratios mislead? Br Med J 1998;**316**(7136):989-91
42. Langhammer A, Krokstad S, Romundstad P, et al. The HUNT study: participation is associated with survival and depends on socioeconomic status, diseases and symptoms. BMC Med Res Methodol 2012;**12**:143 doi: 10.1186/1471-2288-12-143[published Online First: Epub Date]|.
43. Molarius A, Janson S. Self-rated health, chronic diseases, and symptoms among middle-aged and elderly men and women. J Clin Epidemiol 2002;**55**(4):364-70 doi: http://dx.doi.org/10.1016/S0895-4356(01)00491-7[published Online First: Epub Date]|.
44. Hansen AH, Halvorsen PA, Ringberg U, et al. Socio-economic inequalities in health care utilisation in Norway: a population based cross-sectional survey. BMC Health Serv Res 2012;**12**:336 doi: 10.1186/1472-6963-12-336[published Online First: Epub Date]|.
45. Vingilis E, Wade T, Seeley J. Predictors of adolescent health care utilization. J Adolesc 2007;**30**(5):773-800 doi: http://dx.doi.org/10.1016/j.adolescence.2006.10.001[published Online First: Epub Date]|.
46. Breidablik H-J, Meland E, Lydersen S. Self-rated health during adolescence: stability and predictors of change (Young-HUNT study, Norway). The European Journal of Public Health 2009;**19**(1):73-78 doi: 10.1093/eurpub/ckn111[published Online First: Epub Date]|.
47. Welch HG, Sharp SM, Gottlieb DJ, et al. Geographic variation in diagnosis frequency and risk of death among Medicare beneficiaries. JAMA 2011;**305**(11):1113-8 doi: 10.1001/jama.2011.307[published Online First: Epub Date]|.
48. Fisher ES, Wennberg DE, Stukel TA, et al. The implications of regional variations in Medicare spending. Part 2: health outcomes and satisfaction with care. Ann Intern Med 2003;**138**(4):288-98
49. Wennberg JE. *Tracking medicine. A researcher's quest to understand health care.* Oxford: Oxford University Press, 2010.

For peer review only

	n	%	SRH good (%)		*n*	%	SRH good (%)
Age group							
40-50 years	7058	40.3	77.6		6517	40.2	81.7
51-60 years	5709	32.6	64.4		5328	32.8	72.1
61-70 years	4747	27.1	56.6		4375	27.0	59.6
BMI (kg/m^2)							
<18,5	6821	0.6	58.4		4804	0.2	42.9
18,5-24,9	7026	39.1	72.8		8694	29.7	75.3
25,0-29,9	3489	40.3	68.5		2635	53.8	73.6
>30	104	20.0	56.5		35	16.3	64.8
(0.4% missing)							
Smoking status							
Never smoked daily	6973	40.1	70.3		4849	30.1	80.0
Previous daily smoker	4651	26.7	68.0		6105	37.9	69.8
Daily smoker	5763	33.1	64.3		5172	32.0	69.1
(0.7% missing)							
Alcohol use							
None to low intake	14888	88.7	66.7		11488	73.2	71.0
Moderate intake	1471	8.8	76.8		2839	18.1	78.7
High intake	430	2.6	76.3		1375	8.8	78.3
(3.7% missing)							
Educational level							
< 10 years	8133	48.5	60.5		5711	36.5	63.1
10 – 12 years	5682	33.9	72.8		6615	42.2	76.0
> 12 years	2962	17.7	80.0		3339	21.3	84.7
(3.8% missing)							
Employed							
Yes	11539	67.2	77.1		12309	77.1	79.9
No	5627	32.8	49.0		3665	22.9	49.2
(1.8% missing)							

For peer review only

Any long-term impairment							
No	10348	59.1	85.9		9912	63.0	88.7
Yes	6275	35.8	39.8		5829	37.0	46.0
(4.1% missing)							
Thyroid function							
No thyroid disease	14373	86.2	68.7		7804	94.3	72.1
Unknown hypothyroidism	107	0.6	78.5		16	0.2	68.8
Subclinical hypothyroidism	466	2.8	75.0		150	1.8	66.4
Known hypothyroidism	858	5.1	48.9		124	1.5	58.1
Other thyroid dysfunction	872	5.2	61.4		180	2.2	56.2
(1.3% missing)							
Diabetes mellitus							
No diabetes	11279	64.6	69.4		9196	56.9	74.6
Unknown diabetes	46	0.3	52.2		88	0.5	67.8
Probable diabetes	5728	32.8	65.9		6392	39.6	71.5
Known diabetes	395	2.3	43.1		482	3.0	49.5
(0.4% missing)							
Blood pressure status							
Normotensive	9135	52.6	72.4		6858	42.7	77.5
Unknown mild/moderate hypertension	4473	25.8	67.9		5343	33.3	74.7
Unknown severe hypertension	403	2.4	70.7		349	2.2	73.8
Known hypertension	3327	19.2	54.0		3498	21.8	59.6
(0.9% missing)							
Overall study population, HUNT2	34332	97.3	70.3		30378	98.4	74.9

Table 1. Good self-rated health (SRH) by sex and characteristics of the study population.

For peer review only

Table 2. The association between self-rated health and thyroid function, diabetes mellitus and blood pressure. Odds ratio (OR) of good self-rated health and 95% confidence intervals (95% CI), crude, age adjusted, and adjusted for age, other long-term illness or injury that impairs function in everyday life, smoking habits, alcohol use, educational level, work status and body mass index. Cases with missing data were excluded from the analyses.

	**Women**			**OR (95% CI)**	**Men**			**OR (95% CI)**
	n	Crude	Age adjusted	Multiple adjusted	*n*	Crude	Age adjusted	Multiple adjusted
**Thyroid function**								
No thyroid dysfunction	12476	1.00	1.00	1.00	7045	1.00	1.00	1.00
Unknown hypothyroidism	92	1.66 (1.05-2.64)	2.00 (1.25-3.19)	1.84 (1.02-3.33)	14	0.85 (0.30-2.45)	0.92 (0.31-2.72)	1.28 (0.35-4.65)
Subclinical hypothyroidism	394	1.37 (1.10-1.69)	1.49 (1.20-1.85)	1.48 (1.13-1.94)	128	0.77 (0.54-1.08)	0.88 (0.62-1.24)	1.13 (0.72-1.76)
Known hypothyroidism	718	0.44 (0.38-0.50)	0.48 (0.41-0.55)	0.49 (0.41-0.59)	107	0.54 (0.37-0.77)	0.63 (0.43-0.91)	0.69 (0.44-1.09)
Other thyroid dysfunction	729	0.72 (0.63-0.83)	0.77 (0.67-0.89)	0.77 (0.65-0.93)	163	0.50 (0.37-0.67)	0.52 (0.38-0.71)	0.58 (0.41-0.84)
**Diabetes mellitus**								
No diabetes	9946	1.00	1.00	1.00	8464	1.00	1.00	1.00
Unknown diabetes	39	0.48 (0.27-0.86)	0.61 (0.34-1.10)	0.66 (0.32-1.37)	82	0.72 (0.46-1.13)	0.88 (0.55-1.39)	1.03 (0.59-1.81)
Probable diabetes	4797	0.85 (0.80-0.91)	0.94 (0.88-1.01)	1.01 (0.92-1.10)	5759	0.86 (0.80-0.92)	0.94 (0.88-1.01)	0.99 (0.91-1.08)
Known diabetes	318	0.33 (0.27-0.41)	0.42 (0.34-0.51)	0.53 (0.41-0.69)	395	0.33 (0.28-0.40)	0.42 (0.35-0.51)	0.55 (0.43-0.70)
**Blood pressure**								
No hypertension	8180	1.00	1.00	1.00	6378	1.00	1.00	1.00
Unknown mild/moderate hypertension	3787	0.81 (0.75-0.87)	1.01 (0.93-1.09)	1.10 (0.99-1.22)	4837	0.86 (0.79-0.93)	1.01 (0.92-1.10)	1.01 (0.92-1.12)
Unknown severe hypertension	325	0.92 (0.74-1.15)	1.34 (1.08-1.68)	1.52 (1.14-2.02)	308	0.85 (0.77-1.08)	1.27 (0.99-1.62)	1.48 (1.09-2.02)
Known hypertension	2745	0.45 (0.41-0.49)	0.59 (0.54-0.65)	0.69 (0.61-0.77)	3118	0.43 (0.39-0.47)	0.54 (0.50-0.60)	0.64 (0.57-0.72)

For peer review only

STROBE 2007 (v4) Statement—Checklist of items that should be included in reports of *cross-sectional studies*

Section/Topic	Item #	Recommendation	Reported on page #
**Title and abstract**	1	(*a*) Indicate the study's design with a commonly used term in the title or the abstract	2
		(*b*) Provide in the abstract an informative and balanced summary of what was done and what was found	2
**Introduction**			
Background/rationale	2	Explain the scientific background and rationale for the investigation being reported	3-4
Objectives	3	State specific objectives, including any prespecified hypotheses	4
**Methods**			
Study design	4	Present key elements of study design early in the paper	2-3
Setting	5	Describe the setting, locations, and relevant dates, including periods of recruitment, exposure, follow-up, and data collection	5
Participants	6	(*a*) Give the eligibility criteria, and the sources and methods of selection of participants	5
Variables	7	Clearly define all outcomes, exposures, predictors, potential confounders, and effect modifiers. Give diagnostic criteria, if applicable	5-8
Data sources/ measurement	8*	For each variable of interest, give sources of data and details of methods of assessment (measurement). Describe comparability of assessment methods if there is more than one group	5-8
Bias	9	Describe any efforts to address potential sources of bias	7-8
Study size	10	Explain how the study size was arrived at	5
Quantitative variables	11	Explain how quantitative variables were handled in the analyses. If applicable, describe which groupings were chosen and why	5-8
Statistical methods	12	(*a*) Describe all statistical methods, including those used to control for confounding	7-8
		(*b*) Describe any methods used to examine subgroups and interactions	8
		(*c*) Explain how missing data were addressed	Table 2
		(*d*) If applicable, describe analytical methods taking account of sampling strategy	-
		(*e*) Describe any sensitivity analyses	-
**Results**			

For peer review only

Participants	13*	(a) Report numbers of individuals at each stage of study—eg numbers potentially eligible, examined for eligibility, confirmed eligible, included in the study, completing follow-up, and analysed	5
		(b) Give reasons for non-participation at each stage	5
		(c) Consider use of a flow diagram	-
Descriptive data	14*	(a) Give characteristics of study participants (eg demographic, clinical, social) and information on exposures and potential confounders	Table 1
		(b) Indicate number of participants with missing data for each variable of interest	Table 1
Outcome data	15*	Report numbers of outcome events or summary measures	Table 1
Main results	16	(*a*) Give unadjusted estimates and, if applicable, confounder-adjusted estimates and their precision (eg, 95% confidence interval). Make clear which confounders were adjusted for and why they were included	Table 2 Page 9-11
		(*b*) Report category boundaries when continuous variables were categorized	5-7
		(*c*) If relevant, consider translating estimates of relative risk into absolute risk for a meaningful time period	-
Other analyses	17	Report other analyses done—eg analyses of subgroups and interactions, and sensitivity analyses	10-11
**Discussion**			
Key results	18	Summarise key results with reference to study objectives	11
Limitations	19	Discuss limitations of the study, taking into account sources of potential bias or imprecision. Discuss both direction and magnitude of any potential bias	11-13
Interpretation	20	Give a cautious overall interpretation of results considering objectives, limitations, multiplicity of analyses, results from similar studies, and other relevant evidence	13-14
Generalisability	21	Discuss the generalisability (external validity) of the study results	11
**Other information**			
Funding	22	Give the source of funding and the role of the funders for the present study and, if applicable, for the original study on which the present article is based	15

*Give information separately for cases and controls in case-control studies and, if applicable, for exposed and unexposed groups in cohort and cross-sectional studies.

Note: An Explanation and Elaboration article discusses each checklist item and gives methodological background and published examples of transparent reporting. The STROBE checklist is best used in conjunction with this article (freely available on the Web sites of PLoS Medicine at http://www.plosmedicine.org/, Annals of Internal Medicine at http://www.annals.org/, and Epidemiology at http://www.epidem.com/). Information on the STROBE Initiative is available at www.strobe-statement.org.

For peer review only
